# Synthesis, Structure, and Thermal Properties of Volatile Group 11 Triazenides as Potential Precursors for Vapor Deposition

**DOI:** 10.1021/acs.inorgchem.2c03071

**Published:** 2022-12-14

**Authors:** Rouzbeh Samii, Anton Fransson, Pamburayi Mpofu, Pentti Niiranen, Lars Ojamäe, Vadim Kessler, Nathan J. O’Brien

**Affiliations:** † Department of Physics, Chemistry and Biology, Linköping University, Linköping SE58183, Sweden; ‡ Department of Molecular Sciences, Swedish University of Agricultural Sciences, P.O. Box 7015, Uppsala75007, Sweden

## Abstract

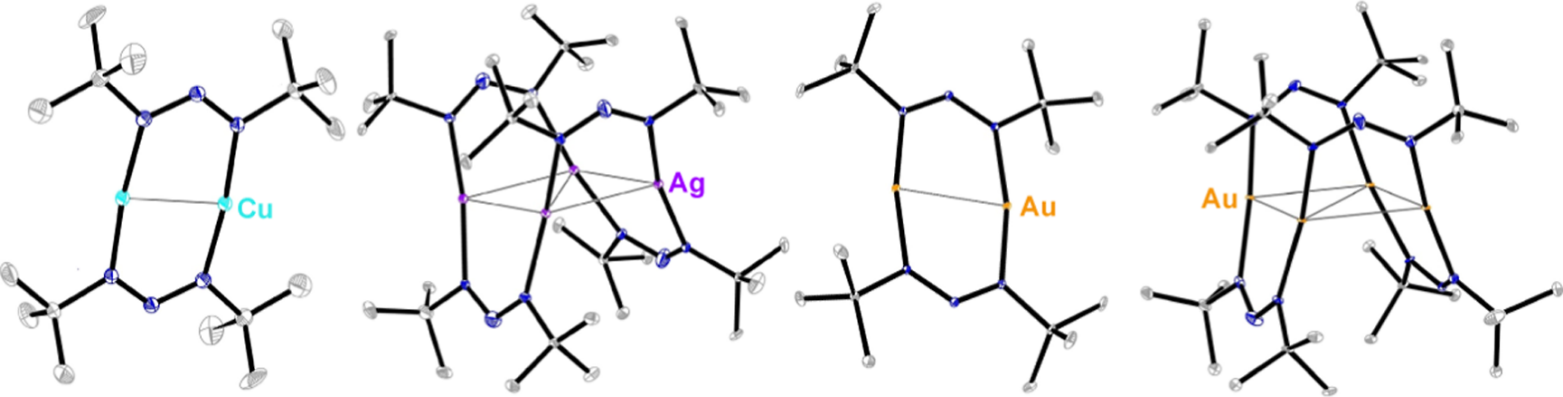

Group 11 thin films are desirable as interconnects in microelectronics. Although many M–N-bonded Cu precursors have been explored for vapor deposition, there is currently a lack of suitable Ag and Au derivatives. Herein, we present monovalent Cu, Ag, and Au 1,3-di-*tert*-butyltriazenides that have potential for use in vapor deposition. Their thermal stability and volatility rival that of current state-of-the-art group 11 precursors with bidentate M–N-bonded ligands. Solution-state thermolysis of these triazenides yielded polycrystalline films of elemental Cu, Ag, and Au. The compounds are therefore highly promising as single-source precursors for vapor deposition of coinage metal films.

## Abstract 2

Group 11 thin films are desirable as interconnects in microelectronics. Although many M−N-bonded Cu precursors have been used for vapor deposition, there is currently a lack of suitable Ag and Au derivatives. In this work, we present monovalent Cu, Ag, and Au triazenides as potential compounds in vapor deposition precursors. Thermal analysis shows that these compounds have thermal stability and volatility that rival current state-of-the-art group 11 precursors with bidentate M−N-bonded ligands.

## Introduction

Thin films of group 11 metals are highly desirable as interconnects in integrated circuits due to their excellent electrical and thermal conductivity and resistance to electron migration.^1^ Furthermore, transparent Ag thin-film electrodes have potential for solar cell applications,^2^ while Au is advantageous for chemical and biological sensors.^3^ Today, Cu, Ag, and Au films are commonly deposited by vapor deposition techniques.^4,5^ Chemical vapor deposition (CVD) and atomic layer deposition (ALD) are two methods currently used to deposit high-quality thin films of group 11 metals. To be successful, both methods require precursors that are sufficiently volatile and thermally stable for transport from the source to the reaction chamber without decomposing. In CVD, the precursors are mixed in the reaction chamber and react, both in the gas phase and on surfaces, to deposit the target material. In ALD, the precursors are added to the system sequentially to allow the process to be governed by self-limiting surface reactions. To date, there are more precursors known for Cu compared to Ag and Au, and thus fewer deposition processes are reported for the latter metals.

**Figure 1:**
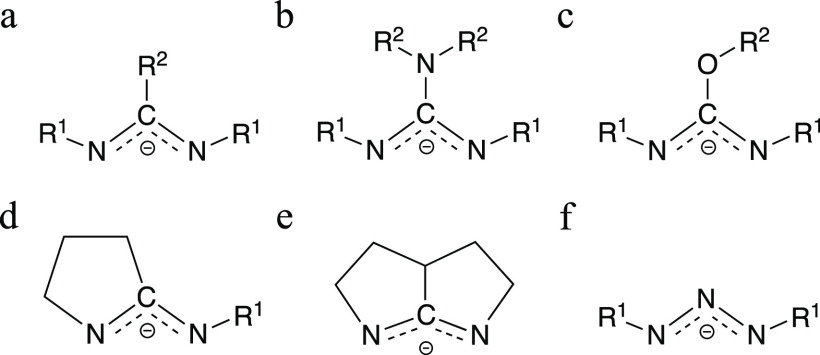
General structure of the bidentate N–M-bonded (a) acyclic amidinate, (b) guanidinate, (c) iso-ureate, (d) monocyclic iminopyrrolidinates, (e) bicyclic amidinate ligands, and (f) triazenide ligand. R_1_ = alkyl, R_2_ = H, alkyl.

The amidinate and guanidinate ligand systems (Figure 1a and 1b, respectively) have been used to produce volatile and thermally stable transition-metal precursors for vapor deposition.^6−9^ A drawback of these precursors is their tendency to decompose via two pathways: β-hydride elimination and carbodiimide (CDI) deinsertion.^10,11^ While β-hydride elimination is easily blocked by having exocyclic *N*-substituents free from β-hydrogens, suppressing CDI deinsertion is more difficult as it involves the endocyclic-carbon substituent. Metallic Cu films have been deposited by ALD using Cu(I) amidinates^6,12−19^ and guanidinates.^20−22^ The Ag(I) and Au(I) amidinates and guanidinates are thermally unstable with respect to CDI deinsertion and therefore have not been successfully used for vapor deposition.^11,23^ A similar ligand to the previous two is the iso-ureate (Figure 1c), which has yielded Cu(I) precursors for CVD of metallic Cu films.^24^ The thermal behavior of these compounds was similar to that of Cu(I) guanidinates and hypothesized to undergo thermolysis via the same mechanism.^21^ The iminopyrrolidinates are monocyclic amidinates, where the R^2^ substituent is bound to the nitrogen forming a pyrrolidine ring (Figure 1d).^10^ Tethered to a nitrogen in the ligand backbone, the R^2^ substituent is difficult to access for CDI deinsertion, making the iminopyrrolidinates more thermally stable than the acyclic amidinates.^11^ Thus, not only have the monovalent group 11 iminopyrrolidinates afforded Cu films by ALD^25^ but also Ag and Au films, with ∼3 at. % carbon, by low-temperature CVD.^5^ Further constrained, bicyclic amidinates (Figure 1e) have been used to yield monovalent group 11 compounds with improved thermal stability over the iminopyrrolidinates.^26,27^ Using these compounds with H_2_ afforded Ag and Au films with ∼6–7 at. % carbon by low-temperature CVD.^27^

An alternative approach to develop new M–N-bonded precursors is to alter the N–C–N ligand backbone. The triazenides differ from the amidinates by having a nitrogen atom in place of the endocyclic carbon (Figure 1f). Replacing the tetravalent carbon with a trivalent nitrogen effectively removes the R^2^ substituent involved in CDI deinsertion and effectively blocking this decomposition pathway. Monovalent group 11 triazenides exist in the literature and all except one possess 1,3-diaryltriazenide ligands.^28−33^ These 1,3-diaryltriazenide examples, however, are most likely not volatile due to their increased intermolecular interactions (e.g., π-stacking) and therefore rendering them unsuitable for vapor deposition. The only 1,3-dialkyl analogue in the literature is a tetranuclear Cu(I) compound with 1,3-dimethyltriazenide ligands, where only melting point (185–186 °C) and structural data have been discussed.^34,35^

Recently, we reported the first examples of volatile group 13 and 14 dialkyltriazenides.^36−40^ The Ga and In triazenides have been used as ALD precursors to afford excellent-quality GaN, InN, InGaN, and In_2_O_3_.^36,37,41−43^ With the success of the triazenide ligand to produce volatile and thermally stable group 13 and 14 compounds, we decided to investigate its reactivity with monovalent coinage metals. Herein, we report the synthesis, structure, and thermal properties of monovalent group 11 triazenides. Their ease to produce, high volatility, and thermal stability make these new precursors highly interesting for use in vapor deposition.

## Results and Discussion

**Scheme 1:**
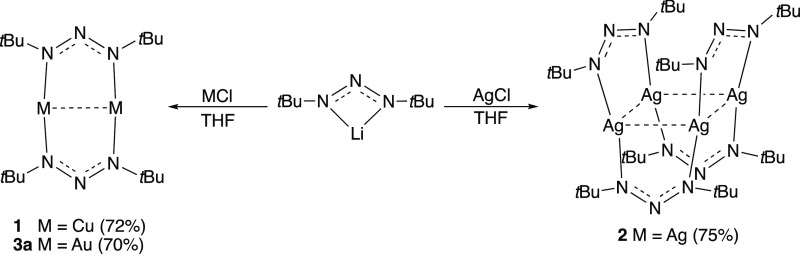
Synthesis of Cu, Ag, and Au Triazenides **1**–**3a**

Compounds **1**, **2**, and **3a** were obtained in good yields by reacting MCl (M = Cu, Ag, Au) with lithium 1,3-di-*tert*-butyltriazenide^39^ in tetrahydrofuran (THF) followed by recrystallization (Scheme 1). The compounds were fully characterized by nuclear magnetic resonance (NMR), elemental analysis, melting or decomposition points, and X-ray crystallography. Crystals of **1–3a** did not degrade when stored for weeks under ambient conditions. When immersed in water for 2 weeks, **1** showed slight green discoloration, while **2** and **3a** remained unchanged.

**Figure 2:**
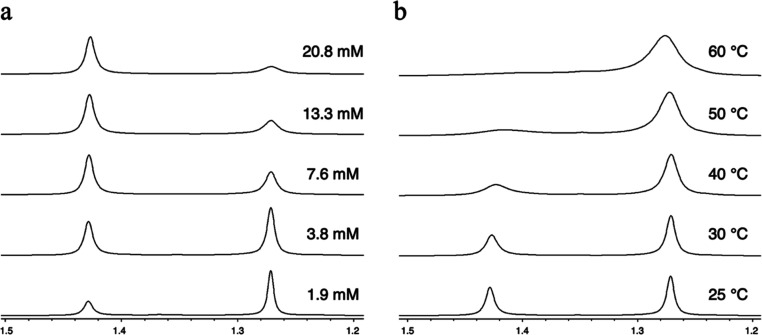
^1^H NMR spectra of **2** in C_6_D_6_ showing the effect of (a) concentration and (b) temperature on the ratio of dinuclear to tetranuclear species (3.8 mM sample).

The ^1^H NMR spectra of **1** and **3a** in C_6_D_6_ showed one singlet (1.27 ppm) suggesting exclusively dinuclear species in solution state. Variable-temperature (VT) NMR showed no line splitting for **1** between −20 and 70 °C. Compound **2** showed two mildly broadened singlets (1.27 and 1.43 ppm) indicative of an equilibrium between di- and tetranuclear forms. Similar di-/tetranuclear and di-/trinuclear equilibria were previously found for Ag formamidinate^44^ and Ag acetamidinate^7^ compounds, respectively. Varying the concentration of **2** changed the di-/tetranuclear ratio. As expected, the relative concentration of the tetranuclear species increased for more concentrated samples of **2** (Figure 2a). Using a coordinating solvent (THF-*d*_8_) did not affect the equilibrium (see the Supporting Information). Using the data, the ambient temperature dissociation constant of **2**, *K*_diss_, was estimated to be 28.7 ± 0.4 mM. VT NMR on a 3.8 mM sample of **2** showed that the equilibrium shifted toward the dinuclear form between 30 and 60 °C (Figure 2b). A van’t Hoff plot of ln[*K*_diss_(*T*)] versus *T*^–1^ gave Δ*H* and Δ*S* of dissociation values of +38.6 kJ mol^−1^ and 98.9 J K^–1^ mol^–1^, respectively (see the Supporting Information).

**Scheme 2:**
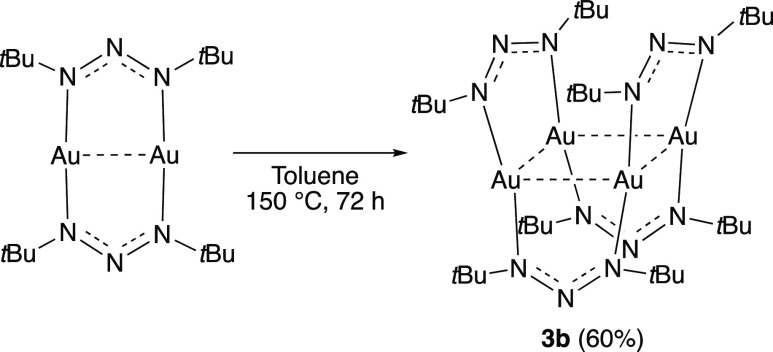
Transformation of **3a** into **3b**

An additional signal emerged at 1.53 ppm for the NMR sample of **3a** when stored at room temperature for months. The new spectra showed two signals, like that of **2**, suggesting a presence of di- and tetranuclear forms of the Au triazenide. No change was observed in the di-/tetranuclear ratio for ^1^H NMR spectra acquired between 5 and 60 °C. The dinuclear form appears to be metastable, and the dinuclear to tetranuclear transformation is slow and, most likely, irreversible. In fact, a pure tetranuclear form, compound **3b**, was obtained by heating a solution of **3a** in toluene to 150 °C for 3 days followed by recrystallization (Scheme 2). Compound **3b** was fully characterized as per compounds **1–3a**.

Diffusion-ordered spectroscopy showed faster diffusion for **1** and **3a** compared to **3b**, which is consistent with **1** and **3a** being dinuclear and **3b** being tetranuclear. Similar results were found for **2**, where the 1.27 ppm species gave faster diffusion than the 1.43 ppm species. However, the 1.27 ppm species showed slower diffusion than **1** and **3a**, while the 1.43 ppm species showed faster diffusion than **3b**, likely an artifact caused by overlap in the chemical shift dimension of the signals in **2**.

**Figure 3:**
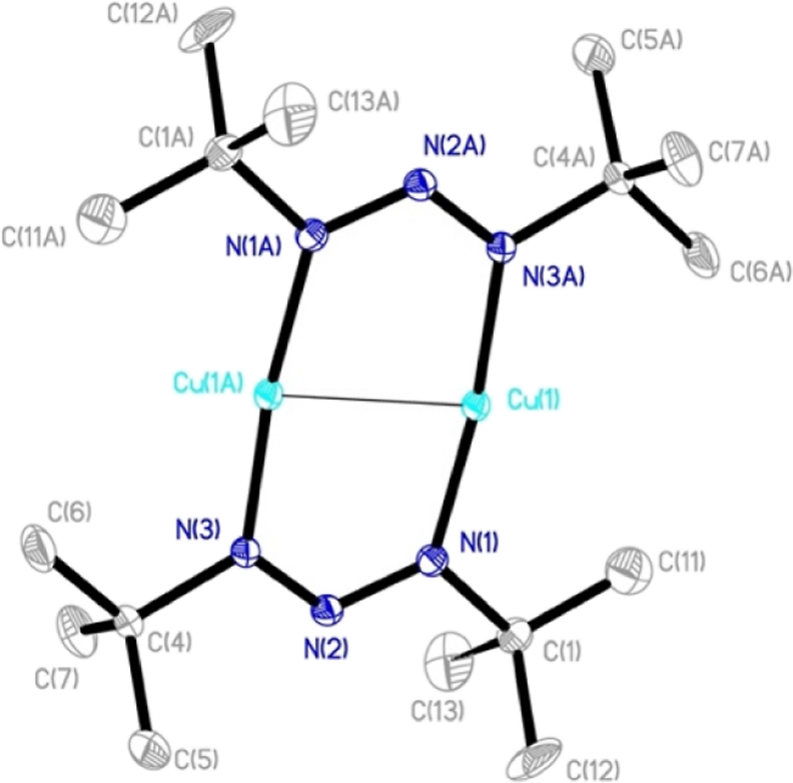
ORTEP drawing of **1**. Thermal ellipsoids at the 50% probability level. All hydrogen atoms were omitted for clarity. Compound **3a** gave an analogous structure molecular geometry analogous dinuclear structure (see the Supporting Information).

X-ray crystallography of **1** and **3a** showed dinuclear structures with two bridging 1,3-di-*tert*-butyltriazenide ligands on opposite sides of the metal centers forming a planar metallacycle (Figure 3). All M–N and N–N bond lengths are equivalent, indicating that the electrons are delocalized over the M and N centers. The structures are consistent with their respective 1,3-bis(2,6-diisopropylphenyltriazenide) (dipp_2_N_3_) analogues, which has the aromatic rings noncoplanar to the metallacycle (Table 1).

**Table 1:** Bond Lengths (Å) and Angles (°) for **1** and **3a**,^32^ the Diphenyl Analogue of **1**, and Di(2,6-diisopropylphenyl)triazenide Analogues of **1** and **3a**(28)

	**1**	dpt Cu	Dipp_2_N_3_ Cu	**3a**	Dipp_2_N_3_ Au
M···M	2.443	2.45	2.446	2.656	2.676
M–N	1.883	1.899, 1.939	1.882	2.050	2.045
N–N	1.292	1.274, 1.316	1.303	1.289	1.302
N–M–N	172.96	171.8	172.63	168.28	168.00
N–N–N	117.73	115.8	115.53	120.50	119.53

**Figure 4:**
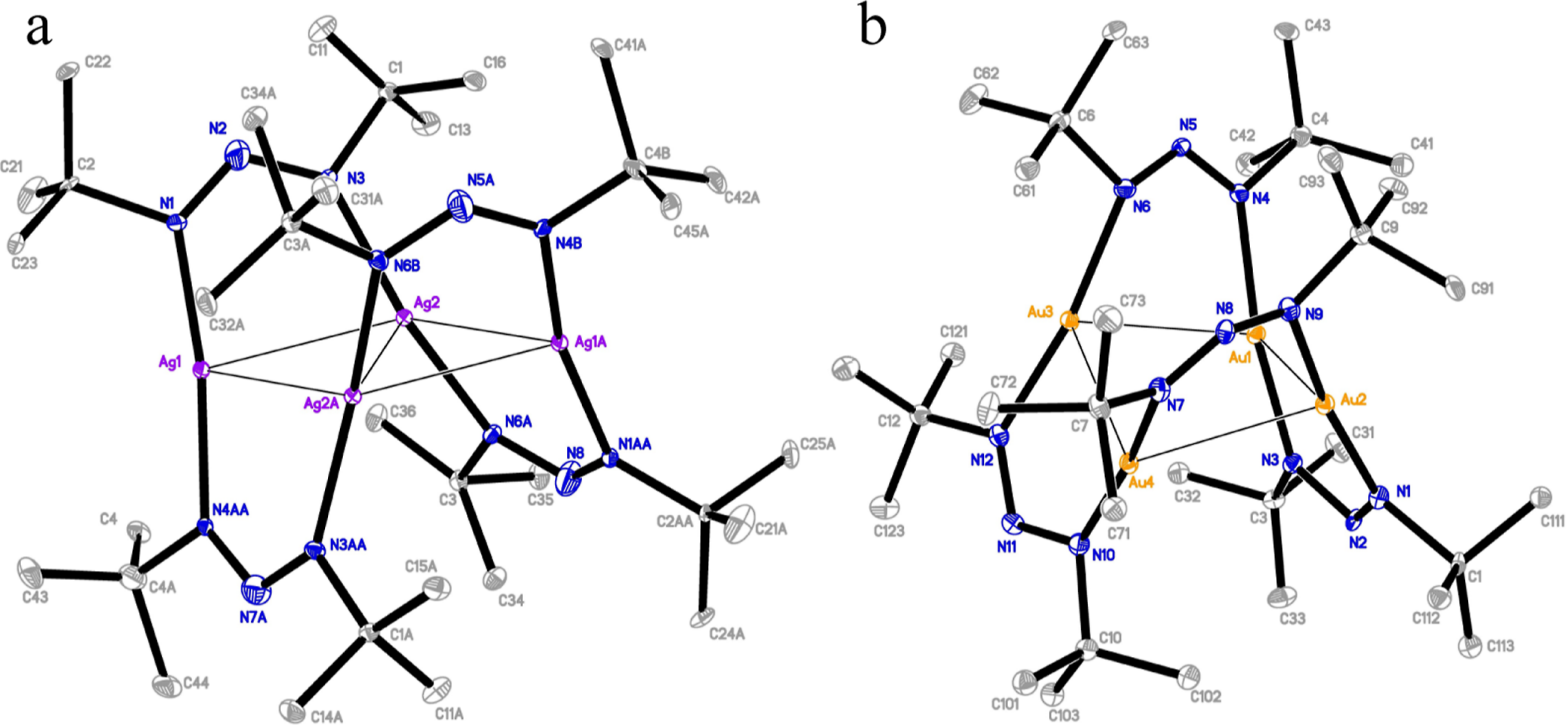
ORTEP drawings of the tetranuclear structures for (a) **2** and (b) the buckled square Au structure **3c** obtained from the residual mother liquor of **3a** after storing for a long period of time. Compound **3b** gave a structure analogous to **2**. Thermal ellipsoids are shown at a 30% probability level. All hydrogen atoms were omitted for clarity.

Both **2** and **3b** showed tetranuclear molecules with the metal centers in rhombus conformations. Four 1,3-di-*tert*-butyltriazenide ligands bridge the metal centers along the perimeter, alternating above and below the plane (Figure 4a). The structures gave similar bond parameters for all but their diagonal M···M distance, and N–M–N and M···M···M angles (Table 2). Most notably, **2** measured a significantly shorter diagonal M···M distance than **3b**. In fact, the diagonal M···M of **2** is only 0.1 Å longer than the average edge M···M, while it is 0.3 Å longer for **3b**. This was accompanied by **2** showing M···M···M angles deviating more from 90° than **3b**. The structures of **2** and **3b** gave similar bond parameters to their respective phenyl analogues, the 4-fluorophenyltriazenide (4F-dpt) Ag (the only other known tetranuclear Ag triazenide in the literature) and dpt Au.^29,33^ However, both **2** and **3b** gave more acute M···M···M angles and therefore shorter diagonal M···M distances.

**Table 2:** Average Bond Lengths (Å) and Angles (°) from X-ray Crystallography for **2** and Its Di(4-fluorophenyl) Analogue,^33^**3b** and the Diphenyl Analogue,^29^ and **3c**

	**2**	4F-dpt Ag	**3b**	dpt Au	**3c**
conformation	rhombic	rhombic	rhombic	rhombic	buckled square
M···M (edge)	2.910	2.821	2.958	2.850	2.921
M···M (diag.)	3.016	3.288	3.254	3.320	4.043
M–N	2.116	2.128	2.066	2.041	2.046
N–N	1.297	1.293	1.289	1.285	1.285^a^
M···M···M (acute)	62.43	71.30	66.75	71.23	87.59
M···M···M (obtuse)	117.57	108.70	113.25	108.62	
N–M–N	162.62	177.7	167.00	176.63	168.52
N–N–N	118.51	118.0	119.09	118.67	119.16
N–M···M–N	2.57	0.43	0.56	3.87^b^	18.0

a One bond length is significantly shorter than the other (1.203 Å). Omitting the shorter bond gives an average *d* = 1.297 Å.

b Two of the ligands are significantly more tilted than the other two (0.74 and 0.34 and 6.26 and 8.14).

Prior to the first structure-determination attempt of **3a**, the crystals were stored for weeks with the residual mother liquor from the recrystallization. As opposed to the dinuclear structure of **3a**, however, a tetranuclear molecule was observed with the Au atoms in a buckled square conformation (Figure 4b). The residual mother liquor must have facilitated the reaction of **3a** into the tetranuclear form, which then crystallized in the buckled square conformation (structure **3c**). Structure **3c** was only observed by X-ray crystallography and differ from **3b** only by adopting a different conformation in the solid state. Thus, any discussion of structure **3c** is limited to the obtained crystal structure.

Structure **3c** showed a significant difference in N–N lengths for two opposing ligands (0.108 and 0.066 Å). A similar, albeit smaller, difference was found in the Au diphenylacetamidinate, which also adopted the buckled square conformation.^45^ The ligands in structure **3c** are twisted relative to the metal centers they bridge, while **3b** showed only minor twisting (N–M···M–N: ∼18 and 2.6°, respectively). Similar buckled square conformation is found in Cu and Ag compounds employing triazenide and bicyclic guanidinate-like ligands, respectively, and are attributed to steric effects.^31,46^

Although serendipitous, it is unclear exactly why the buckled square conformation **3c** was obtained, rather than rhombic seen for **3b**. It may be solvent-dependent; however, the tetranuclear species most likely converts rapidly between the buckled square and rhombic conformation in solution state, which is supported by ^1^H NMR of **2** and **3b** showing only one signal for the tetranuclear species at 25 °C. We made no attempts to replicate these results.

### DFT and NBO Calculations

The DFT geometries of **1**–**3** are in good agreement with their respective crystal structures (see the Supporting Information). Singlet electron configurations gave far better agreement than triplet configurations. Rhombic **2** showed the largest deviation, where the optimized geometry gave Ag···Ag···Ag angles closer to 90° and, consequently, a longer diagonal Ag···Ag distance compared to the crystal structure (see the Supporting Information). Among the functionals used, LC-ωHPBE yielded a geometry with the best match for rhombic **2** and was therefore used for all calculations. Structures were also optimized for dinuclear **2** and **3** and rhombic **1** and **3** to calculate the electronic energy difference (Δ*E*_0_) between the two different structures (eq 1).
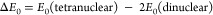
1

Preference for the tetranuclear form increases in the order **1** < **2** < **3** where **1** slightly favored the dinuclear form, while **2** and **3** favored the rhombic (Table 3). The Δ*E*_0_ was negligible between rhombic and buckled square conformations of the tetranuclear Au structures.

**Table 3:** Δ*E*_0_ and Δ*G* (kcal mol^–1^) between One Tetranuclear Rhombic and Two Dinuclear Structures for **1–3**

	**1**	**2**	**3**
Δ*E*_0_	–1.05	–6.51	–15.5
Δ*G*	17.2	14.5	7.94

Due to the decrease in entropy upon combining two dinuclear species into a tetranuclear, the difference in Gibbs free energy (Δ*G*) for **1–3** (calculated analogously to Δ*E*_0_) favored the dinuclear structures in the gas phase and increased in the order **3** < **2** < **1**. Thus, if **1–3** form di-/tetranuclear equilibria, the dinuclear structures are expected to dominate in the gas phase. However, while **2** showed an equilibrium in C_6_D_6_ by NMR, **3** did not and was even isolated in its di- and tetranuclear forms (**3a** and **3b**, respectively).

The optimized structures of **1–3** were investigated by natural bond orbital (NBO) analysis. Natural bond orders for dinuclear **1–3** from natural resonance theory calculations agree with the expected formal bond orders. The M–N bonds showed high ionic character, ∼100, 84, and 74% for dinuclear **1**, **2**, and **3**, respectively. Wiberg bond indices (WBIs) showed significantly greater M–N bond order for the Au structures **3** than that for **1** and **2** (Table 4). The greater M–N WBI for **3** is consistent with NMR experiments of **2** showing a di-/tetranuclear equilibrium while **3** does not. WBI suggests small M···M interactions for **1**–**3**, ranging between 0.04 and 0.06 (see the Supporting Information).

**Table 4:** Average M···M and M–N WBI for Dinuclear and Tetranuclear **1–3**

	dinuclear			rhombic			square
	**1**	**2**	**3**	**1**	**2**	**3**	**3**
M···M (edge)	0.053	0.062	0.053	0.045	0.044	0.035	0.036
M···M (diag.)	N/A	N/A	N/A	0.040	0.024	0.009	N/A
M–N	0.269	0.258	0.372	0.227	0.233	0.361	0.361

The metal center natural charges for **1–3** decreased in the order **1** > **2** ≫ **3** (Table 5), as expected based on electron affinities for Cu–Au. Rhombic **1** gave ∼0.02 au greater metal center charge than the dinuclear structure. In contrast, dinuclear **2** showed greater charges than rhombic **2** but with a smaller difference (∼0.01 au), while **3** showed similar metal center charges between di- and tetranuclear structures (difference < 0.005 au).

**Table 5:** Average Natural Charges (in au) for the Metal Center (M), Triazenide Backbone (N_3_), and Ligands (R-Group) for Di- and Tetranuclear **1–3**

	dinuclear			rhombic			square
	**1**	**2**	**3**	**1**	**2**	**3**	**3**
M	0.655	0.635	0.491	0.678	0.627	0.489	0.487
N_3_	–0.961	–0.931	–0.831	–0.993	–0.937	–0.834	–0.830
R-group	0.153	0.148	0.170	0.158	0.155	0.172	0.172

Second-order perturbation theory analysis (donor–acceptor interactions) from the NBO analysis was used to compare stabilization energies (*E*^(2)^) for donor–acceptor interactions of rhombic **1**–**3** with two of their respective dinuclear structures (eq 2).
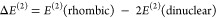
2

Interactions involving valence acceptor orbitals stabilized dinuclear **1–3** more than their respective tetranuclear forms (Δ*E*^(2)^ < 0 kcal mol^–1^) due to more favorable orbital overlap. For example, geminal n1_N_ → n_M_* (which is essentially σ_MN_) greatly favored dinuclear **1–3** over rhombic, and Δ*E*^(2)^ decreased in the order **3** < **2** < **1** (Table 6). In general, the dinuclear forms were favored for interactions involving valence acceptors where the number of interactions remained unchanged between two dinuclear and a rhombic structure (e.g., geminal M–L). The geminal M···M interactions, however, favored the rhombic structures as they compensated for their lower *E*^(2)^ by having more interactions.

**Table 6:** Δ*E*^(2)^ (kcal mol^–1^) for Two Interactions Involving Valence Acceptor Orbitals and Two Involving Rydberg Acceptor Orbitals **1–3**

	N1_N_ → n_M_*	n_M_^sd^ → n_M_*	n_M_^sd^ → RY1_N_	n_M_^sd^ → RY3_N_
**1**	–150	3.81	–2.87	3.43
**2**	–99	34.8	38.9	48.8
**3**	–56	3.35	7.15	39.9

Interactions involving Rydberg acceptor orbitals often favored rhombic **1–3** over dinuclear (Δ*E*^(2)^ > 0 kcal mol^–1^) due to similar orbital overlap or sometimes even larger overlap for the rhombic structures. Vicinal and remote interactions favored the rhombic structures due to their larger number of interactions over two dinuclear. In contrast to **2** and **3**, structures of **1** interacted poorly with Rydberg acceptors and gave modest Δ*E*^(2)^. Therefore, rhombic **1** was not stabilized sufficiently to compensate for the destabilization relative to the dinuclear form (e.g., n1_N_ → n_M_*), while Rydberg acceptors had larger impact on rhombic **2** and **3**. This may explain why the crystal structure of **1** showed a dinuclear structure, while **2** and **3** were tetranuclear. See the Supporting Information for more details on the NBO analysis.

To summarize, in the context of second-order perturbation theory, the rhombic structures must adapt and retain orbital overlap from the dinuclear structure, particularly for the geminal M–N. Rhombic **1–3** showed more M···M interactions than two of their respective dinuclear structures. Strong M···M interactions therefore favor one rhombic structure over two dinuclear. For the same reason, strong vicinal and remote interactions favor the rhombic structures. Last, if accessible, Rydberg acceptor levels are highly impactful, favoring the rhombic structures by having comparable orbital overlap between the dinuclear and rhombic forms.

### Thermal Analysis

**Figure 5:**
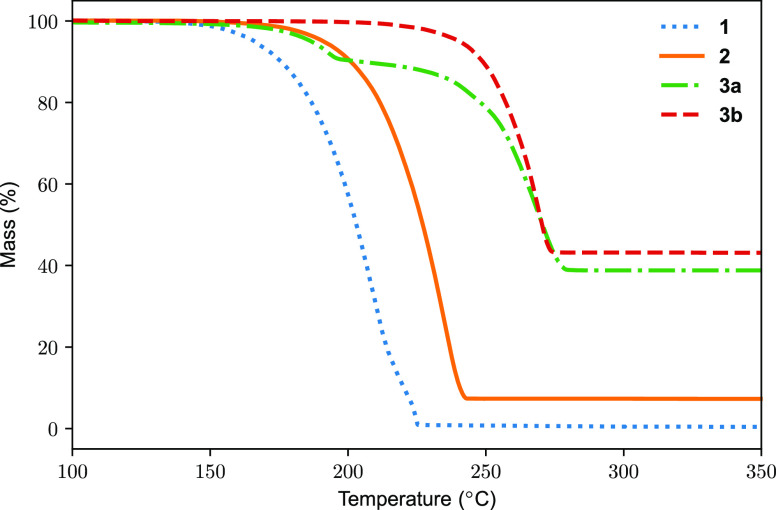
TGA ramp experiments for compounds **1**, **2**, **3a**, and **3b** using 10 mg samples and a heating rate of 10 °C min^–1^. Irregularities are seen for **1** (dotted, blue) at ∼220 °C, possibly due to melting of the sample.

Compounds **1**, **2**, and **3a** sublimed between 120 and 130 °C at 0.5 mbar, while **3b** sublimed between 140 and 160 °C, all quantitatively. The similar sublimation temperatures suggest that **1**, **2**, and **3a** volatilize in their dinuclear forms. Additionally, **3a** and **3b** remained unchanged by NMR after sublimation which suggests that these sublime in dinuclear and tetranuclear forms, respectively. Thermogravimetric analysis (TGA) ramp experiments showed one-step volatilization for **1**, **2**, and **3b** (Figure 5) where **1** and **3b** gave the lowest and highest onset, respectively (Table 7). Compound **2** showed considerably lower onset than **3b** which supports the suggestion that the tetranuclear **2** first forms dinuclear species that then volatilize. A similar two-step process was postulated for the bicyclic Ag amidinate to explain why its volatilization kinetics resembled that of the dinuclear bicyclic Cu amidinate.^27^ Compound **3a** showed two mass loss events. The first and second steps coincide with that of **2** and **3b**, respectively. Most likely, dinuclear molecules are volatilized in the first step. This volatilization is disrupted at ∼160–200 °C to give the first slanting plateau as **3a** is depleted to form **3b**. As the temperature increases further, **3b** gains sufficient heat to volatilize, leading to the second mass-loss event. Compound **1** gave a residual mass <1%. In contrast, **2** gave 7% residual mass, suggesting a small degree of thermolysis at elevated temperatures. Compounds **3a** and **3b** showed more extensive thermolysis (39 and 43% residual mass, respectively). The residual masses of **3a** and **3b** are lower than the mass % Au in their empirical formula (∼56%), indicating that there is some volatilization of Au species.

**Table 7:** Summary of the Thermal Properties of Compounds **1–3**^a^

	onset of volatilization	residual mass (%)	subl. temp.	decomp. temp.
**1**	181	1	120–130	229–230
**2**	209	7	120–130	193–210
**3a**	248^b^	39	120–130	180–185
**3b**	248	43	140–160	185–190

a Temperatures are given in °C.

b The onset of the second step is displayed for **3a** as the first step would give an unfair estimation of the onset compared to the other compounds.

The outlet gas of the TGA was monitored using a mass spectrometer scanning for *m*/*z* 57 and 99 (matching *t*Bu^+^ and *t*BuN_3_^+^, respectively). In all cases, mass-loss steps gave an increased signal for *m*/*z* 57. Additionally, **2** and **3a** showed a strong and weak signal, respectively, for *m*/*z* 99. For **3a**, the second step showed significantly stronger signals for both *m*/*z* 57 and 99 than the first step (see the Supporting Information). It is likely that detection of *t*BuN_3_^+^ occurs during thermolysis of the compounds. Comparing TGA ramp experiments performed under similar conditions showed that **1–3** are more volatile than their corresponding mono- and bicyclic amidinates.^26,47^ The Ag triazenide **2** gave a residual mass similar to the cyclic Ag amidinates (>10% for 10 mg samples).^5,27^ The Au compounds **3a** and **b** gave a comparable residual mass to the cyclic Au amidinates (∼40% for **3a** and **3b**, compared to 40 and 25% for the mono- and bicyclic Au amidinate, respectively).^5,27^

**Figure 6:**
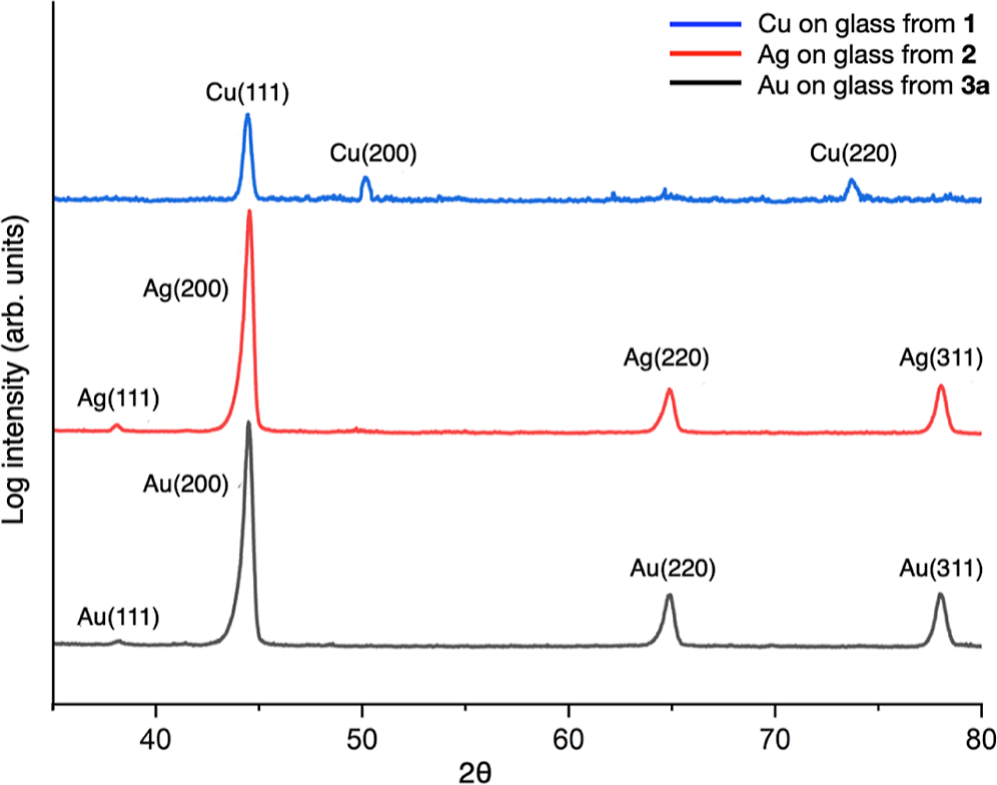
X-ray powder diffractogram of the films deposited on the walls of the NMR tubes after thermolysis of **1**, **2**, and **3a**, respectively.

Solutions of **1–3a** in toluene-*d*_8_ were heated between 100 and 230 °C in flame-sealed heavy-walled NMR tubes and monitored periodically by ^1^H NMR. The change in integrals was tracked using the residual solvent as reference (see the Supporting Information). Compound **1** showed minor changes below 180 °C. The tube walls turned slightly yellow above 140 °C. This discoloration became more pronounced at 190 °C, and additional signals appeared in the base line by NMR. Above 190 °C, these signals became stronger as the signal of **1** diminished more rapidly. However, **1** was still seen after 3 h at 230 °C. Compound **2** started decomposing at 120 °C, and a metallic coating formed on the tube walls. The signals of **2** diminished steadily between 130 and 160 °C to give a few singlets, a broad feature at 1.0–1.3 ppm and a multiplet at 4.69–4.72 ppm. Much of **2** remained at 160 °C; however, lock issues prevented further NMR experiments. Compound **3a** converted into **3b** already at 100 °C (singlet at 1.49 ppm). The solution was held at 120 °C for ∼48 h which transformed most of **3a** into **3b**. A dark brown or black precipitate formed during the transformation; however, only traces of impurity signals appeared as **3b** formed. A golden metallic film was observed on the tube walls at 140 °C. Upon further heating, the trace of **3a** slowly diminished. Meanwhile, **3b** remained steady below 180 °C and then diminished rapidly between 180 and 200 °C to give several high-intensity singlets. These new signals matched those observed for the thermolysis of **1**. After complete thermolysis of **1**, **2**, and **3a**, metallic films had deposited on the interior of the NMR tubes (see the Supporting Information). X-ray powder diffraction revealed the films to be polycrystalline elemental Cu, Ag, and Au (Figure 6). The ability of these precursors to form elemental films upon thermolysis makes them promising candidates as single-source precursors for vapor deposition of coinage metal films. This is substantial as usually a reducing reagent, such as H_2_ gas, is required to reduce the metal center of the precursor to ground state.^48^

Solid samples of **1**, **2**, and **3a** were flame sealed under vacuum and heated to 120 °C. After 7 days, **1** appeared visually unaffected; no solid particles were seen when dissolving in C_6_D_6_, and ^1^H NMR showed no byproducts from thermolysis. In contrast, the crystals of **2** and **3a** had visibly darkened after a few hours of heating. After 7 days, **2** and **3a** had turned black and slightly purple, respectively, yet **2** remained reflective while **3a** did not. Both **2** and **3a** was partially insoluble in C_6_D_6_, and ^1^H NMR showed a few impurity signals. Additionally, for **3a**, the signal of **3b** had emerged; however, the formation of **3b** was slower in solid state than that observed in solution state.

## Conclusions

In conclusion, we have presented the first example of volatile Cu, Ag, and Au triazenides (**1**, **2**, and **3**, respectively). These compounds showed thermal properties on par with the current state-of-the-art ALD and CVD precursors in the same class but were easier to prepare. Compound **1** showed dinuclear structures. Meanwhile, **2** and **3** showed both dinuclear and tetranuclear (rhombic) forms. In solution state, **2** formed a di-/tetranuclear equilibrium while **3** did not. Compound **3** was isolated in both di- and tetranuclear forms (**3a** and **3b**, respectively). Overtime, **3a** transformed into **3b**. In solution state, this transformation happened over many weeks at room temperature or days at 120–150 °C. In solid state, **3a** only transformed at elevated temperature (120 °C). The compounds sublimed quantitatively at reduced pressure, seemingly even the Au analogues. Compounds **1**, **2**, and **3a** sublimed between 120 and 130 °C at 0.5 mbar, and **3b** sublimed between 140 and 160 °C. The sublimation temperatures suggest that **1**, **2**, and **3a** sublime in the dinuclear form, while **3b** sublimes in the tetranuclear form, which is supported by TGA ramp experiments, DFT calculations, and NBO analysis. Additionally, solution-state thermolysis of **1–3** yielded elemental group 11 films by X-ray powder diffraction, suggesting that the ligand acts as a reducing agent. Thus, **1–3** may potentially be used as single-source precursors for CVD of elemental Cu, Ag, and Au films. Overall, these compounds provide an easy-to-produce alternative to the current state-of-the-art amidinates, as potential ALD and CVD precursors. The 1,3-dialkyltriazenide ligand system is easy to derivatize, allowing it to be tuned as desired.

## Experimental Section

### General Comments

*Caution! As catenated nitrogen compounds are known to be associated with explosive hazards,* tert*-butylazide, lithium (1,3-di-tert-butyltriazenide) and compounds****1–3****are possible explosive energetic materials. Although we have not experienced any difficulties or problems in the synthesis, characterization, sublimation, and handling of these compounds, their energetic properties have not been rigorously investigated and are therefore unknown. We therefore highly recommend all appropriate standard safety precautions for handling explosive materials (safety glasses, face shield, blast shield, leather gloves, polymer apron, and ear protection) be always used when working with these compounds.* All reactions and manipulations were carried out under a nitrogen atmosphere on a Schlenk line using Schlenk air-free techniques and in a Glovebox-Systemtechnik dry box. All anhydrous solvents were purchased from Sigma-Aldrich and further dried with 4 Å molecular sieves. CuCl (99.995%), AgCl (99.999%), and Me_2_S·AuCl were purchased from Sigma-Aldrich and used without further purification. Lithium 1,3-di-*tert*-butyltriazenide was synthesized according to the literature procedure.^39^ All NMR spectra were measured with an Oxford Varian and Bruker Avance Neo 500 MHz spectrometers. Solvents peaks were used as an internal standard for the ^1^H NMR and ^13^C{^1^H} NMR spectra. The decomposition points were determined in melting point tubes sealed under N_2_ with a Stuart SMP10 melting point apparatus and are uncorrected. Elemental analysis was performed by Mikroanalytisches Laboratorium Kolbe, Germany.

### Synthesis of Dinuclear (1,3-Di-*tert*-butyltriazenide)copper(I) **1**

A solution of lithium 1,3-di-*tert*-butyltriazenide (4.95 g, 3.03 mmol) in THF (20 mL) was added to a suspension of CuCl (3.00 g, 3.03 mmol) in THF (80 mL), and the reaction mixture was heated to 80 °C for 24 h in a pressure tube. After cooling down to room temperature, the mixture was concentrated under reduced pressure to give a yellow and brown solid residue. The residue was dissolved in *n*-hexane, filtered through a bed of Celite, and concentrated under reduced pressure to give the crude product as a solid. The crude was recrystallized from *n*-hexane at −35 °C to give **1** as a yellow solid (4.82 g, 72%).

**1**: Yellow crystals, mp: 229–230 °C. Sublimation: 120–130 °C (0.5 mbar). ^1^H NMR (500 MHz, C_6_D_6_): δ 1.27 (s, 36H, C*H*_3_). ^1^H NMR (500 MHz, toluene-*d*_8_): δ 1.25 (s, 36H, C*H*_3_). ^13^C{^1^H} NMR (125 MHz, C_6_D_6_): δ 30.8 (s, *C*H_3_) and 58.8 (s, *C*_q_). Anal. Calcd for C_16_H_36_Cu_2_N_6_: C, 43.72%; H, 8.25%; and N, 19.12%. Found: C, 43.70%; H, 8.27%; and N, 19.11%.

### Synthesis of Dinuclear/Tetranuclear (1,3-Di-*tert*-butyltriazenide)silver(I) **2**

A solution of lithium 1,3-di-*tert*-butyltriazenide (8.56 g, 5.25 mmol) in THF (50 mL) was added to an aluminum-foil-wrapped reaction flask containing a solution of AgCl (7.52 g, 5.25 mmol) in THF (100 mL). The reaction mixture was stirred at room temperature for 48 h and then concentrated under reduced pressure to give a solid residue. The residue was purified by vacuum sublimation at 120–130 °C and 0.5 mbar to give **2** as a pale-yellow solid (10.4 g, 75%). For characterization, the sublimed solid of **2** (∼1.0 g) was recrystallized from toluene at −35 °C.

**2**: Pale yellow crystals, decomp. 193–210 °C. Sublimation: 120–130 °C (0.5 mbar). ^1^H NMR (500 MHz, C_6_D_6_, 13.3 mM): δ 1.27 (s, 36H, C*H*_3_, dinuclear, 48%) and 1.43 (s, 72H, *CH*_3_, tetranuclear, 52%). ^1^H NMR (500 MHz, THF-*d*_8_, 13.3 mM): δ 1.21 (s, 36H, C*H*_3_, dinuclear, 51%) and 1.29 (s, 72H, *CH*_3_, tetranuclear, 49%). ^1^H NMR (500 MHz, toluene-*d*_8_): δ 1.25 (s, 36H, C*H*_3_, dinuclear) and δ 1.39 (s, 36H, C*H*_3_, tetranuclear). ^13^C{^1^H} NMR (125 MHz, C_6_D_6_): δ 31.1 (*C*H_3_, dinuclear), 32.0 (*C*H_3_, tetranuclear), 58.3 (s, *C*_q_, dinuclear), and 60.5 (s, *C*_q_, tetranuclear). Anal. Calcd for C_32_H_72_Ag_4_N_12_: C, 36.38%; H, 6.87%; and N, 15.91%. Found: C, 36.25%; H, 6.91%; and N, 15.84%.

### Synthesis of Dinuclear (1,3-Di-*tert*-butyltriazenide)gold(I) **3a**

A solution of lithium 1,3-di-*tert*-butyltriazenide (0.28 g, 1.73 mmol) in THF (30 mL) was added to an aluminum-foil-wrapped reaction flask containing a solution of (Me_2_S)AuCl (0.51 g, 1.73 mmol) in THF (30 mL) at −78 °C. The reaction mixture was stirred at −78 °C for 30 min and at room temperature for 16 h and then concentrated under reduced pressure to give a solid residue. The residue was suspended in *n*-hexane, filtered through a pad of Celite, and concentrated under reduced pressure to give the crude product as a solid. The crude was recrystallized from *n*-hexane to give **3a** as a solid (0.42 g, 70%).

**3a**: Yellow crystals, decomp. 180–185 °C. Sublimation: 120–130 °C (0.5 mbar). ^1^H NMR (500 MHz, C_6_D_6_): δ 1.27 (s, 36H, C*H*_3_). ^1^H NMR (500 MHz, toluene-*d*_8_): δ 1.24 (s, 36H, C*H*_3_). ^13^C{^1^H} NMR (125 MHz, C_6_D_6_): δ 30.5 (s, *C*H_3_) and 62.5 (s, *C*_q_). Anal. Calcd for C_16_H_36_Au_2_N_6_: C, 27.20%; H, 5.14%; and N, 11.90%. Found: C, 27.18%; H, 5.13%; and N, 11.87%.

### Synthesis of Tetranuclear (1,3-Di-*tert*-butyltriazenide)gold(I) **3b**

A solution of **3a** (0.20 g, 0.57 mmol) in toluene (5 mL) was added to an aluminum-foil-wrapped pressure tube and heated to 150 °C for 3 days while stirring. The mixture was filtered through a pad of Celite and concentrated under reduced pressure to give the crude product as a solid. The crude was recrystallized from toluene at −35 °C to give **3b** as a yellow solid (0.12 g, 60%).

**3b**: Yellow crystals, decomp. 185–190 °C. Sublimation: 135–165 °C (0.5 mbar). ^1^H NMR (500 MHz, C_6_D_6_): δ 1.53 (s, 72H, C*H*_3_). ^1^H NMR (500 MHz, toluene-*d*_8_): δ 1.49 (s, 72H, C*H*_3_). ^13^C{^1^H} NMR (125 MHz, C_6_D_6_): δ 31.9 (s, *C*H_3_) and 64.3 (s, *C*_q_). Anal. Calcd for C_32_H_72_Au_4_N_12_: C, 27.20%; H, 5.14%; and N, 11.90%. Found: C, 27.12%; H, 5.11%; and N, 11.81%.

### Variable-Concentration and -Temperature ^1^H NMR Experiments with **2**

For the variable-concentration and -temperature ^1^H NMR experiments, solutions of nine different concentrations (1.9, 3.8, 5.7, 7.6, 9.5, 11.4, 13.3, 17.0, and 20.8 mM) were prepared by dissolving the required amount of **2** in 1.0 mL of C_6_D_6_. A ∼400 μL aliquot of each solution was transferred to an NMR tube and used for ^1^H NMR analysis. The samples were analyzed using 5 °C increments from +25 to +60 °C (see the Supporting Information).

### Diffusion-Ordered Spectroscopy

Diffusion measurements were performed for **1–3b** using bipolar double-stimulated echo pulse sequences with longitudinal eddy-current delay (dstebpgp3s in TopSpin).^49^ The following pulse sequence parameters were used for **1** and **3a**: relaxation delay = 5 s, diffusion time Δ = 50 ms, gradient pulse length δ/2 = 0.700 ms, and eddy-current delay = 5 ms. The same parameters were used for **2** and **3b**, with modifications in Δ (75 and 100 ms, respectively) and δ/2 (1.000 and 1.200 ms, respectively). Measurements were performed at *z*-gradient strengths varied linearly between 1 and 47 G/cm in 16 increments, with 32 scans at each increment. The diffusion coefficients were extracted using Dynamic Center 2.8.1.

### X-ray Crystallographic Analysis

Single crystals were obtained by recrystallization from *n*-hexane at −35 °C for **1**, **3a**, and **3c** and from toluene at −35 °C for **2** and **3b**. The single crystals were used for X-ray diffraction data collection at 163 K for **1**, **2**, and **3a**; 296 K for **3b**, and 153 K for **3c** on a Bruker D8 SMART Apex-II diffractometer using graphite-monochromated Mo-Kα radiation (λ = 0.71073 Å). All data were collected in hemisphere with over 95% completeness to 2θ < 50.05°. The structures were solved by direct methods. The coordinates of metal atoms were determined from the initial solutions and the N and C atoms by subsequent differential Fourier syntheses. All nonhydrogen atoms were refined first in isotropic and then in anisotropic approximation using Bruker SHELXTL software. Selected crystal data are summarized below.

**1**: C_16_H_36_Cu_2_N_6_, *M* = 439.59, orthorhombic, space group *Pbca*, *a* = 11.172(2), *b* = 10.683(2), *c* = 19.277(4) Å, *V* = 2300.7 Å^3^, *Z* = 4, *D*_c_ = 1.269 cm^–3^, μ = 1.858 mm^–1^, *T* = 163 *K*, 2079 unique reflections measured, 1740 observed [*I* > 2σ(*l*)], final *R*1 = 0.0355, w*R*2 (all data) = 0.1052, GOF = 0.921.

**2**: C_32_H_72_Ag_4_N_12_, *M* = 1056.48, monoclinic, space group *P*2_1_/*n*, *a* = 11.726(6), *b* = 11.201(5), *c* = 17.369(8) Å, α = 90, β = 105.396(5), γ = 90°, *V* = 2199.5(2) Å^3^, *Z* = 2, *D*_c_ = 1.595 cm^–3^, μ = 1.790 mm^–1^, *T* = 163 K, 3869 unique reflections measured, 3347 observed final [*I* > 2σ(*l*)], *R*1 = 0.0510, w*R*2 (all data) = 0.1465, GOF = 1.025.

**3a**: C_16_H_36_Au_2_N_6_, *M* = 706.45, orthorhombic, space group *Pbca*, *a* = 11.5117(4), *b* = 10.7482(3), *c* = 18.1941(5) Å, *V* = 2251.16(12) Å^3^, *Z* = 4, *D*_c_ = 2.084 cm^–3^, μ = 13.029 mm^–1^, *T* = 163 K, 1971 unique reflections measured, 1909 observed [*I* > 2σ(*l*)], final *R*1 = 0.0370, w*R*2 (all data) = 0.1090, GOF = 1.220.

**3b**: C_32_H_72_Au_4_N_12_, *M* = 1412.88, monoclinic, space group *P*2_1_/*n*, *a* = 11.6588(10), *b* = 11.2592(9), *c* = 17.3240(14) Å, *V* = 2190.6(3) Å^3^, *Z* = 2, *D*_c_ = 2.114 cm^–3^, μ = 13.389 mm^–1^, *T* = 296 K, 3169 unique reflections measured, 3050 observed [*I* > 2σ(*l*)], final *R*1 = 0.062, w*R*2 (all data) = 0.1878, GOF = 1.177.

**3c**: C_32_H_72_Au_4_N_12_, *M* = 1412.88, orthorhombic, space group *P*2_1_2_1_2_1_, *a* = 10.445(4), *b* = 10.462(4), *c* = 41.456(14) Å, *V* = 4530(3) Å^3^, *Z* = 4, *D*_c_ = 2.071 cm^–3^, μ = 12.948 mm^–1^, *T* = 153 K, 7925 unique reflections measured, 6320 observed [*I* > 2σ(*l*)], final *R*1 = 0.0921, w*R*2 (all data) = 0.2242, GOF = 0.996.

CCDC 2152400 for **1**, 2152401 for **2**, 2189288 for **3a**, 2189289 for **3b** (rhombic conformation), and 2152402 for **3b** (buckled square conformation) contain supplementary crystallographic data for this paper. These data can be obtained free of charge from the Cambridge Crystallographic Data Centre.

### Thermogravimetric Analysis-Mass Spectrometry

TGA was performed on alumina pans with a TA instrument Discovery TGA 55. The pans were rinsed with ethanol and then heated by a propane torch until white hot. All TGA experiments were performed under a flow of ultrapure argon (99.999%, 100 sccm). Samples were heated to 500 °C at a rate of 10 °C min^–1^. The onset of volatilization was defined as the intersection between the tangent line of the plateau and slope. The TGA was connected to a Pfeiffer ThermoStar mass spectrometer.

### Solution-State Thermolysis

Solutions of **1–3a** in toluene-*d*_8_ (40 g L^–1^ for **1** and **3a**, and 20 g L^–1^ for **2**) were added to heavy-walled NMR tubes and degassed by freeze–thaw–pump cycles until no more gas bubbles formed upon thawing. The tubes were then flame sealed and wrapped in Al foil to avoid light exposure. The flame-sealed tubes were heated between 100 and 220 °C in 10 °C increments and monitored by ^1^H NMR experiments on every hour of heating if not otherwise stated.

### X-ray Powder Diffraction

The X-ray powder diffractograms were obtained using a Malvern Panalytical Empyrean diffractometer in a symmetric θ/2θ configuration, operating at a voltage of 45 kV and current of 40 mA, and using a Cu Kα X-ray source (λ = 1.5406 Å). Ni foil was used to filter the Kβ radiation.

### Quantum Chemical Computations

All quantum chemical calculations were preformed using Gaussian 16.^50^ Geometry optimizations and harmonic normal-mode vibrational calculations were performed using the long-range corrected hybrid DFT method LC-ωHPBE^51,52^ and def2TZVP^53,54^ basis set. Solvation was accounted for using the SMD continuum solvation model.^55^ Minima were confirmed to have no imaginary frequencies. NBO analysis was performed on the minimized structures using NBO 7.1^56^ interfaced from Gaussian 16.

## Supporting Information Available

Characterization of the compounds and computational calculation details (PDF)
