# Supplementary material for: Synthesis, Structure, and Thermal Properties of Volatile Group 11 Triazenides as Potential Precursors for Vapor Deposition
Source: Inorg Chem. 2022 Dec 14;61(51):20804–13. doi: 10.1021/acs.inorgchem.2c03071 (PMC9795554; doi:10.1021/acs.inorgchem.2c03071)
Supplement: Supplementary file 1 — ic2c03071_si_001.pdf [file ic2c03071_si_001.pdf]

Synthesis, Structure and Thermal Properties of Volatile Group 11 Triazenides as Potential Precursors for Vapor Deposition

Rouzbeh Samii,[1] Anton Fransson,[1] Pamburayi Mpofu,[1] Pentti Niiranen,[1] Lars Ojamäe,[1] Vadim Kessler,[2] and Nathan J. O'Brien[1,*]

[1]Department of Physics, Chemistry and Biology, Linköping University, Linköping SE-58183,

[2]Department of Molecular Sciences, Swedish University of Agricultural Sciences, P.O. Box 7015, 75007 Uppsala, Sweden

*E-mail: nathan.o.brien@liu.se

Table of contents

NMR Spectral Charts	S2–7
K_{diss} and van't Hoff Plots	S8–9
Solution-State Thermolysis	S9–12
Diffusion-Ordered Spectroscopy	S12
Thermogravimetric Analysis-Mass Spectrometry	S13–14
X-ray Crystallography	S15
Computational Details	S16–61
References	S62

NMR Spectral Charts

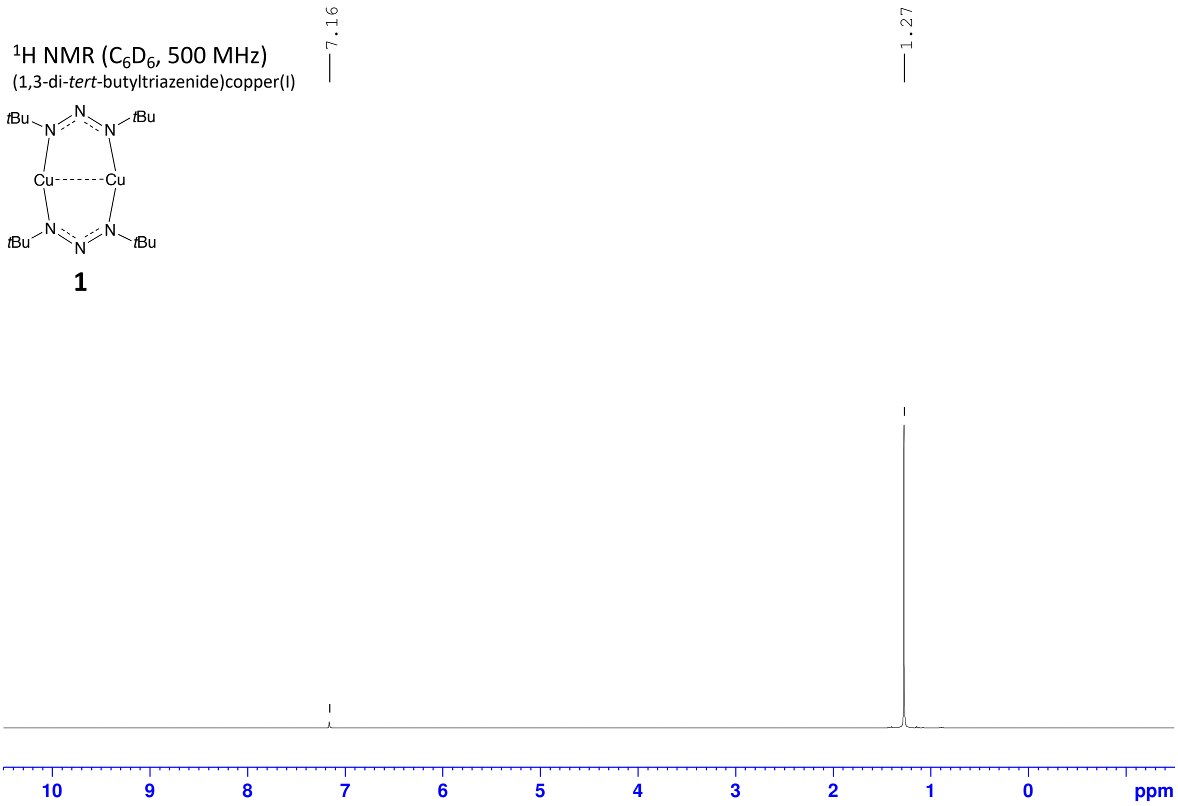

Figure S1: ^{1}H NMR spectrum of **1** in C_6D_6 at 25 °C.

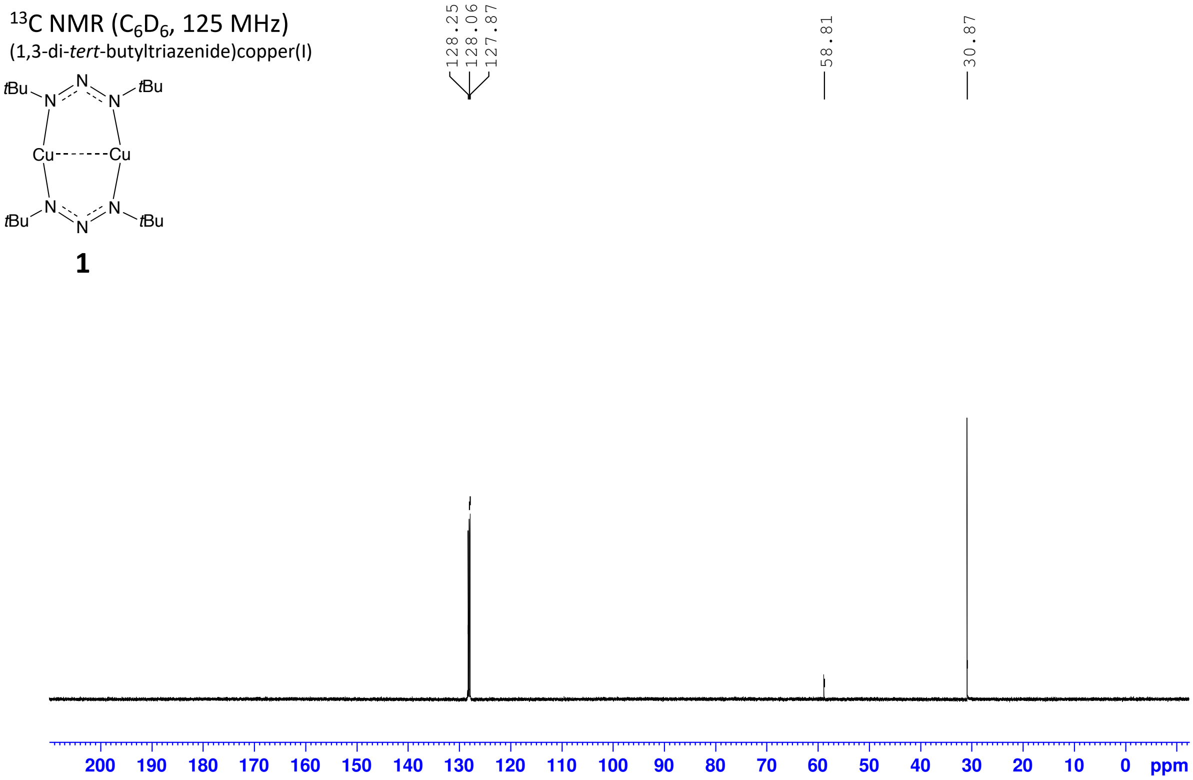

Figure S2: The ^{13}C NMR spectrum of **1** in C_6D_6 at 25 °C.

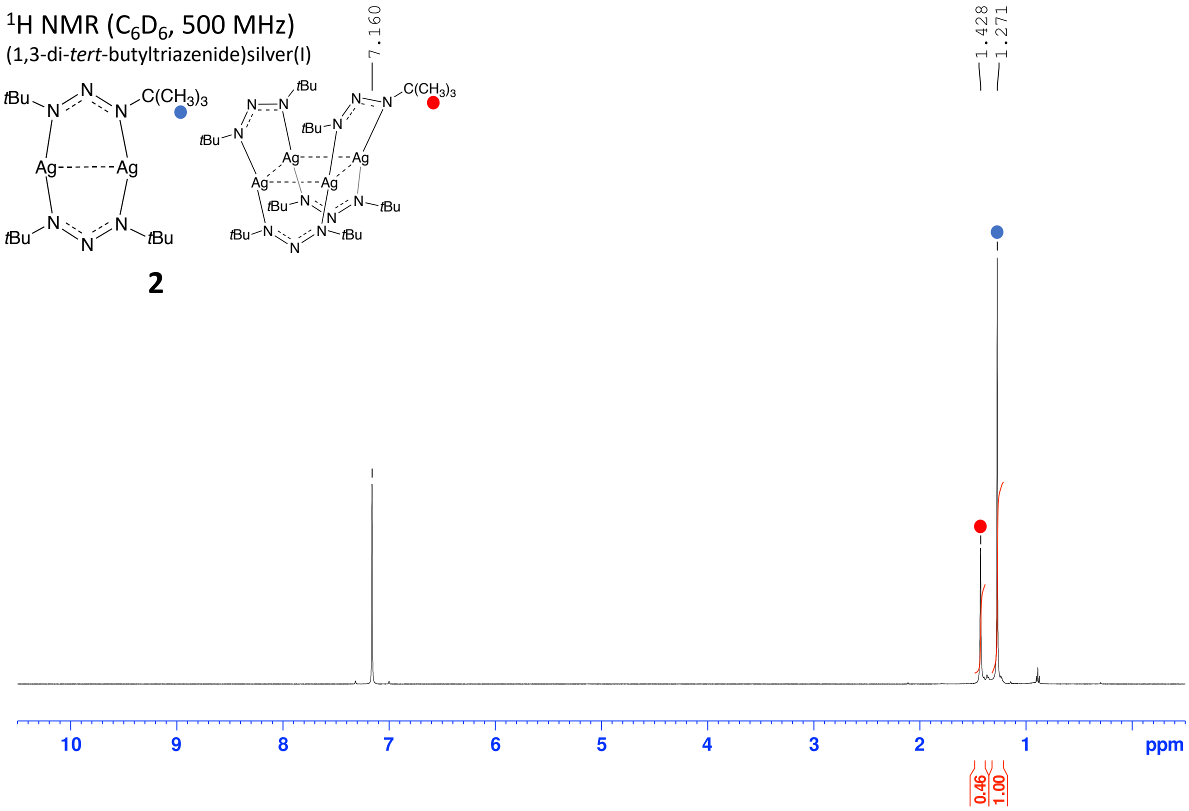

Figure S3: The ^{1}H NMR spectrum of **2** (1.9 mM) in C_6D_6 at 25 °C.

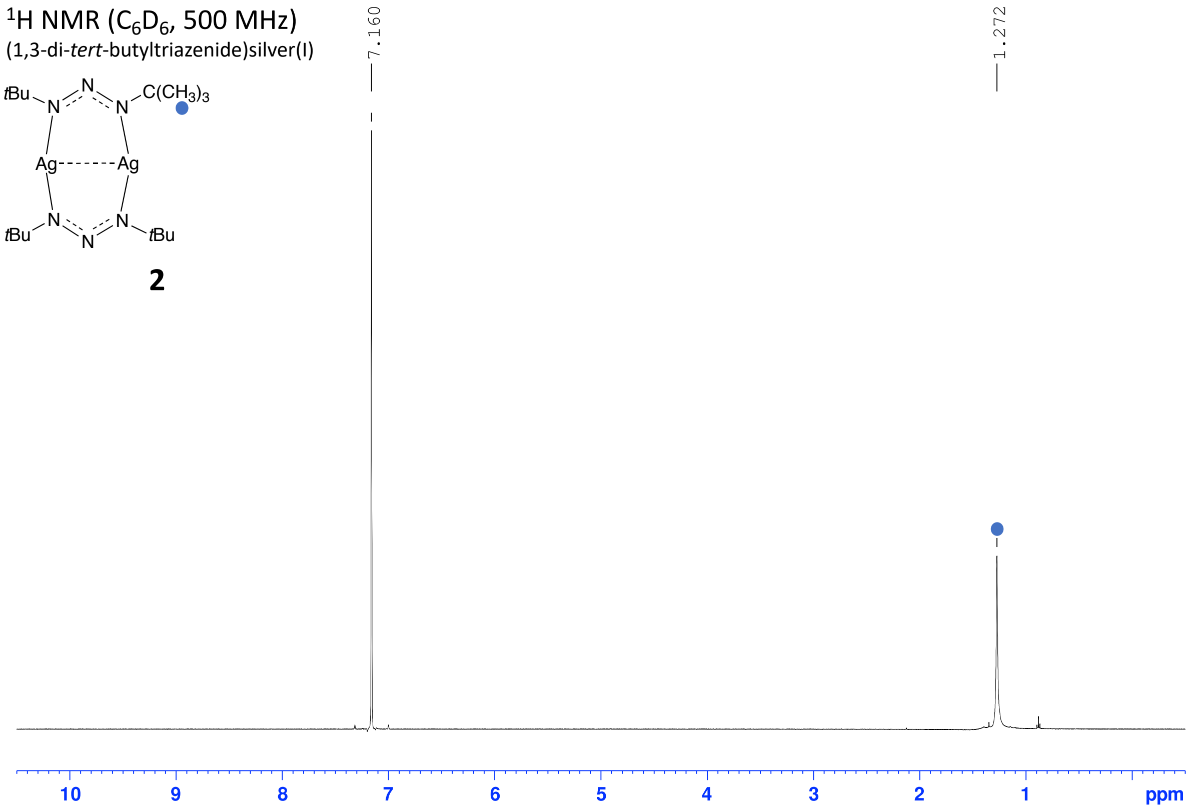

Figure S4: The ^{1}H NMR spectrum of **2** (1.9 mM) in C_6D_6 at 60 °C.

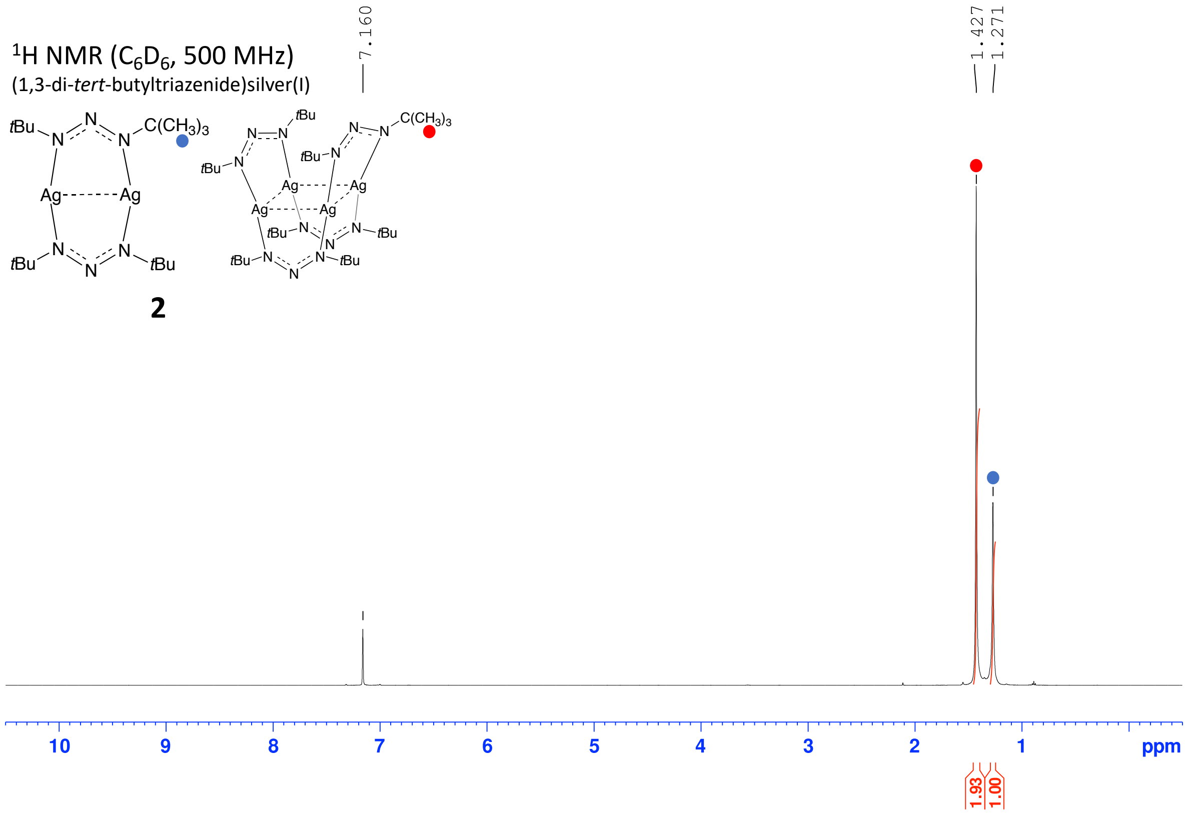

Figure S5: The ^{1}H NMR spectrum of **2** (13.3 mM) in C_6D_6 at 25 °C.

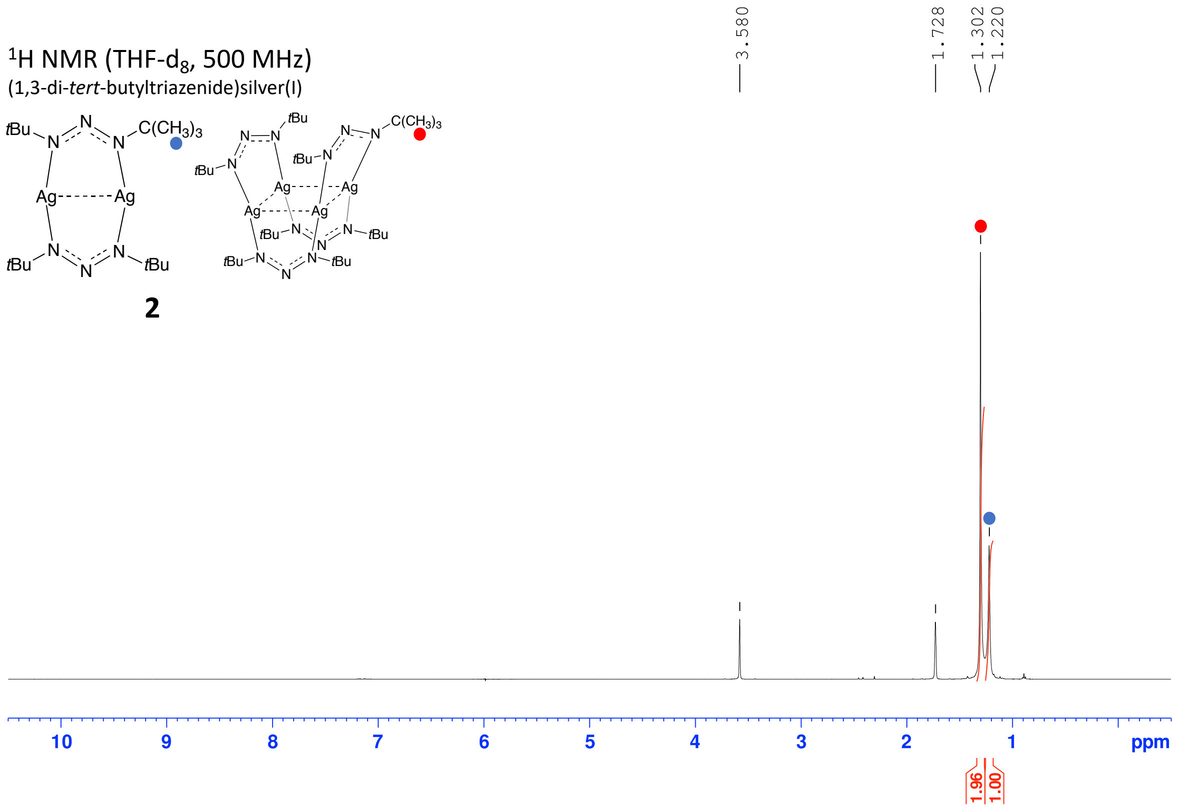

Figure S6: The ^{1}H NMR spectrum of **2** (13.3 mM) in THF-d_8 at 25 °C.

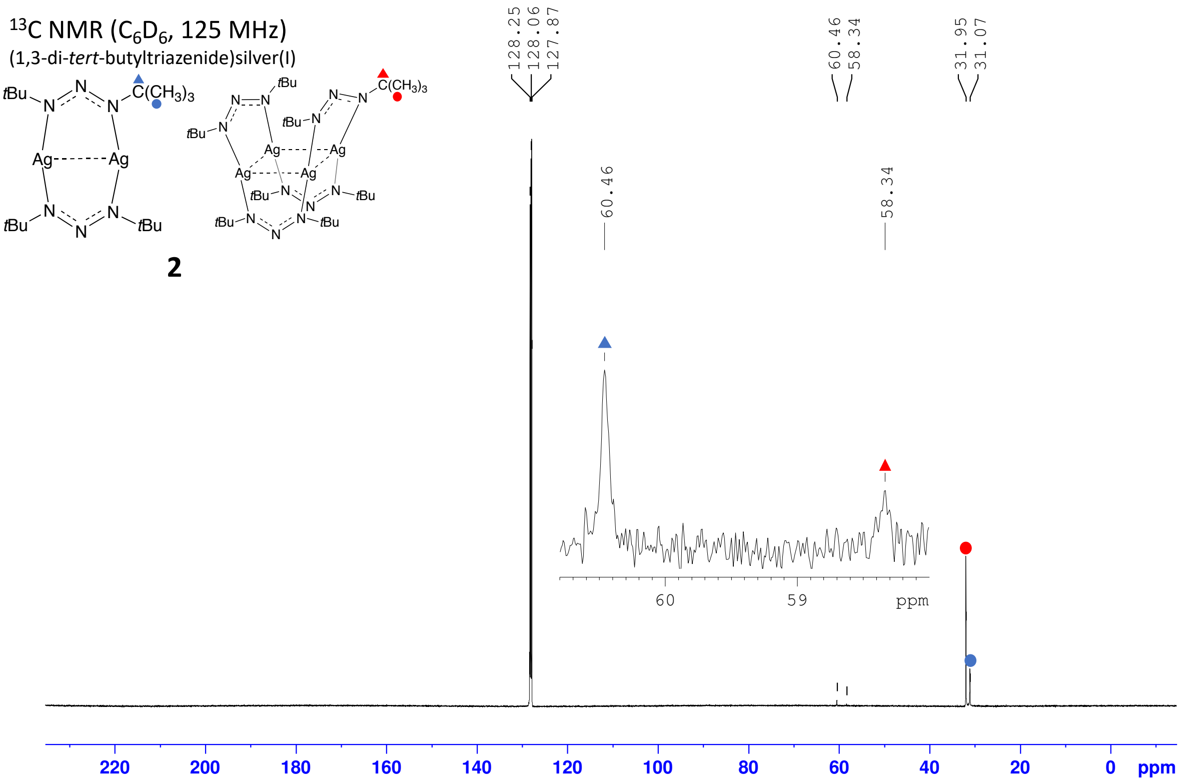

Figure S7: The ^{13}C NMR spectrum of **2** (20.8 mM) in C_6D_6 at 25 °C.

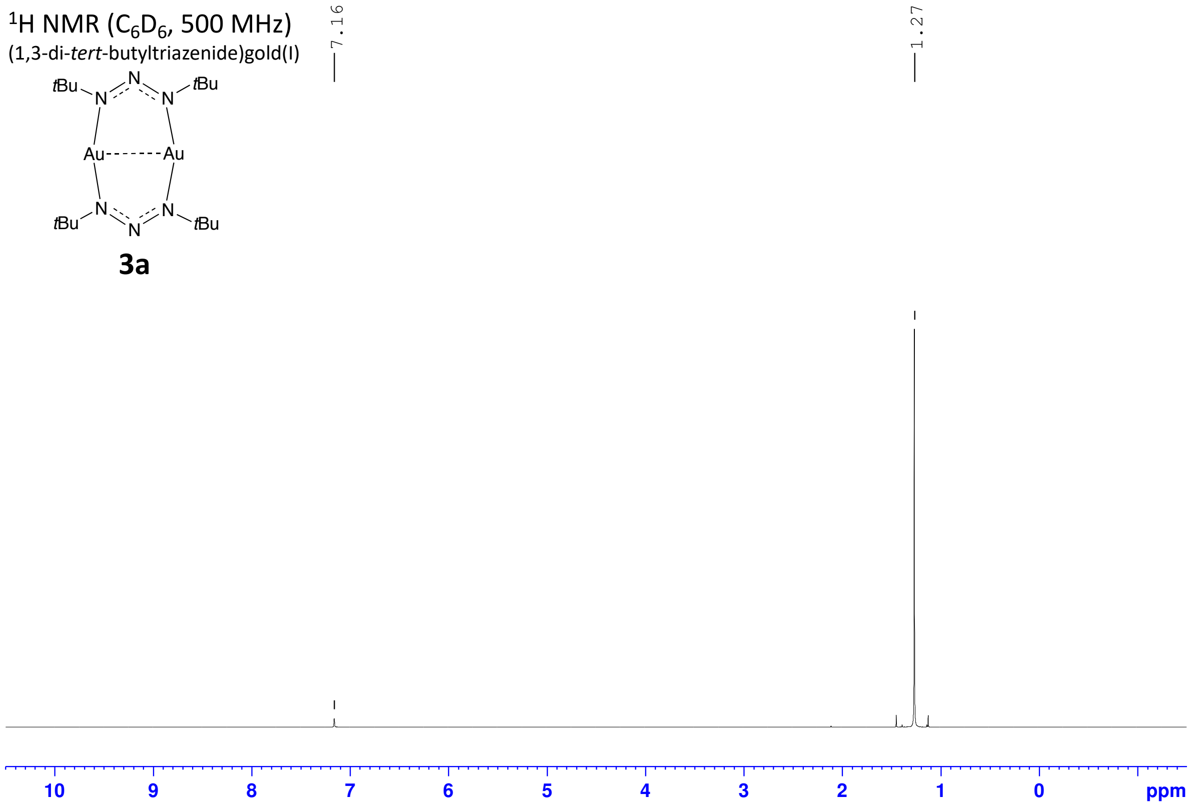

Figure S8: The ^{1}H NMR spectrum of **3a** in C_6D_6 at 25 °C.

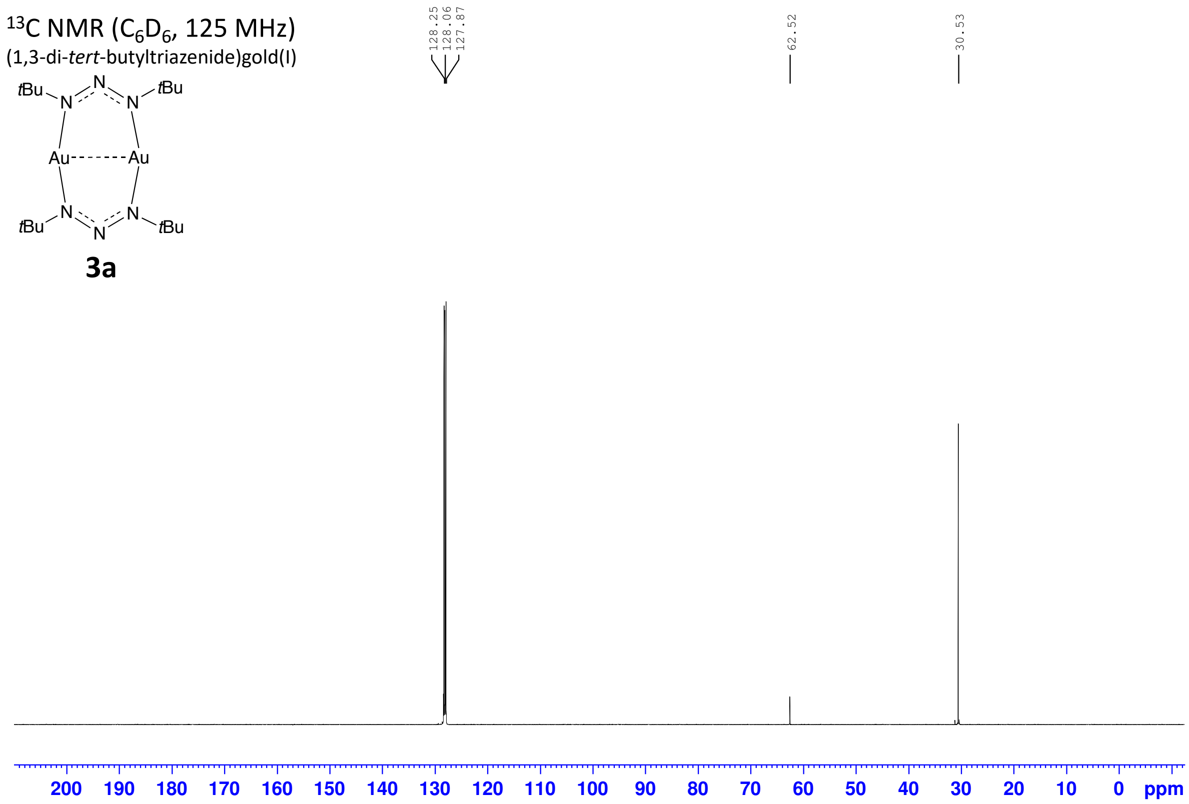

Figure S9: The ^{13}C NMR spectrum of **3a** in C_6D_6 at 25 °C.

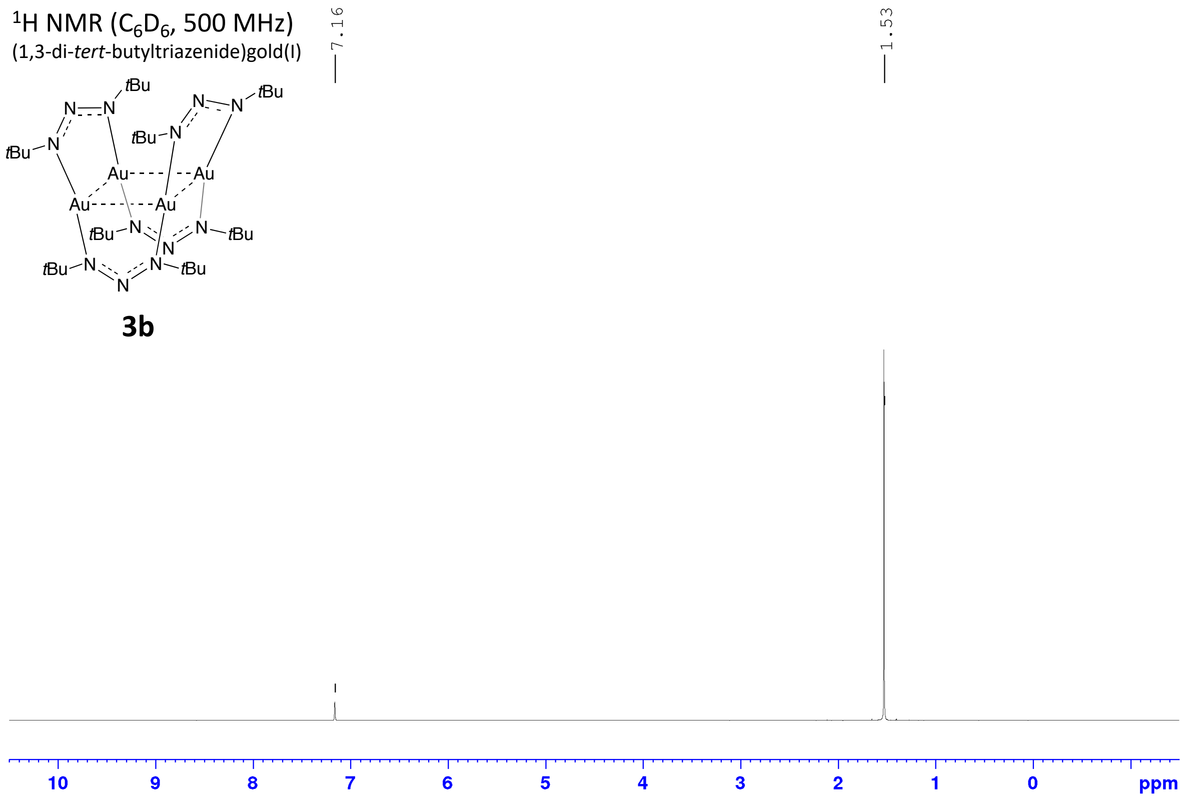

Figure S10: The ^{1}H NMR spectrum of **3b** in C_6D_6 at 25 °C.

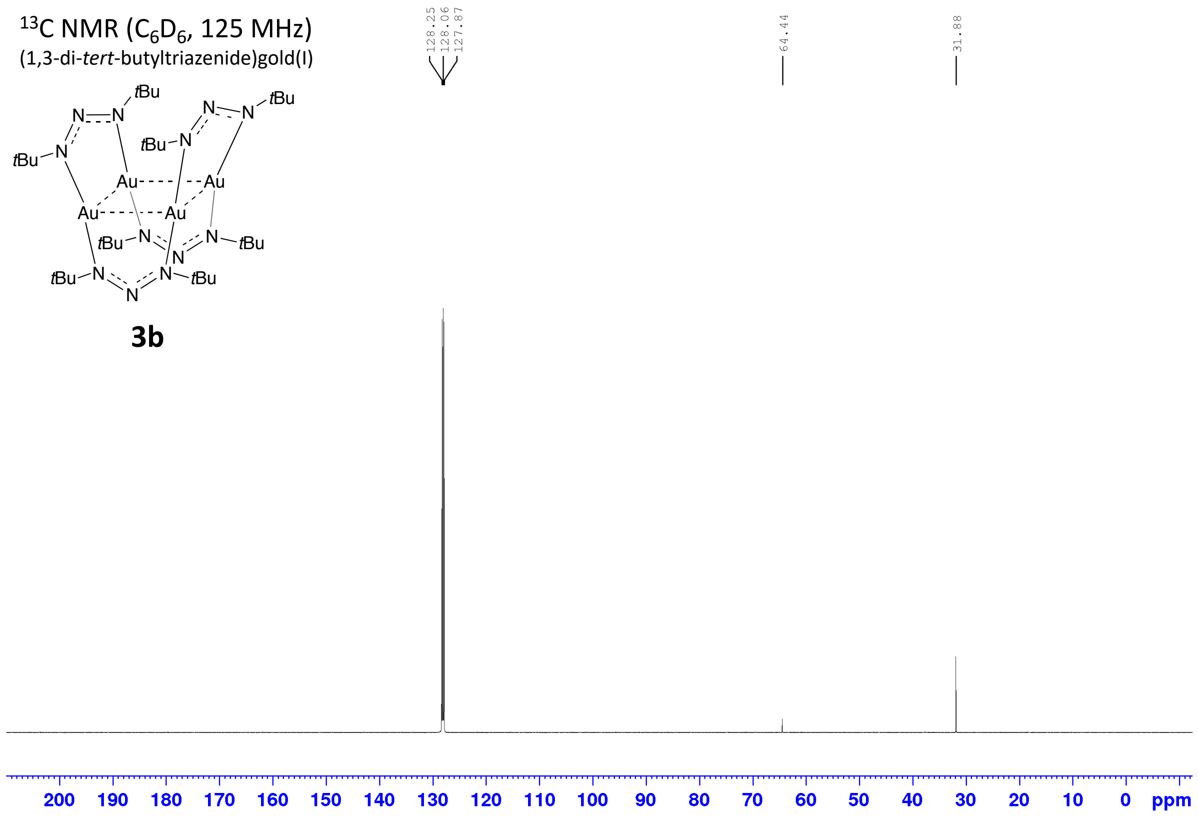

Figure S11: The ^{13}C NMR spectrum of **3b** in C_6D_6 at 25 °C.

K_{diss} and van't Hoff Plots

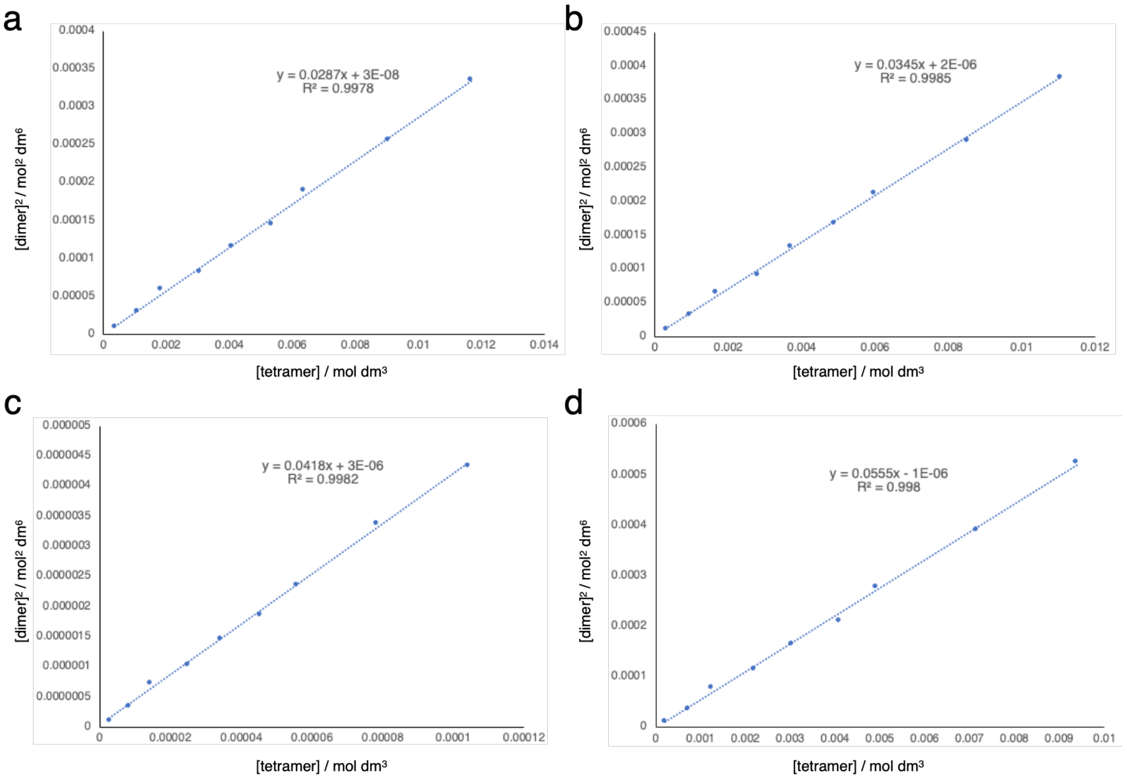

Figure S12: The plot of dinuclear vs. tetranuclear concentration of **2** at a) 25, b) 30, c) 35 and d) 40 °C.

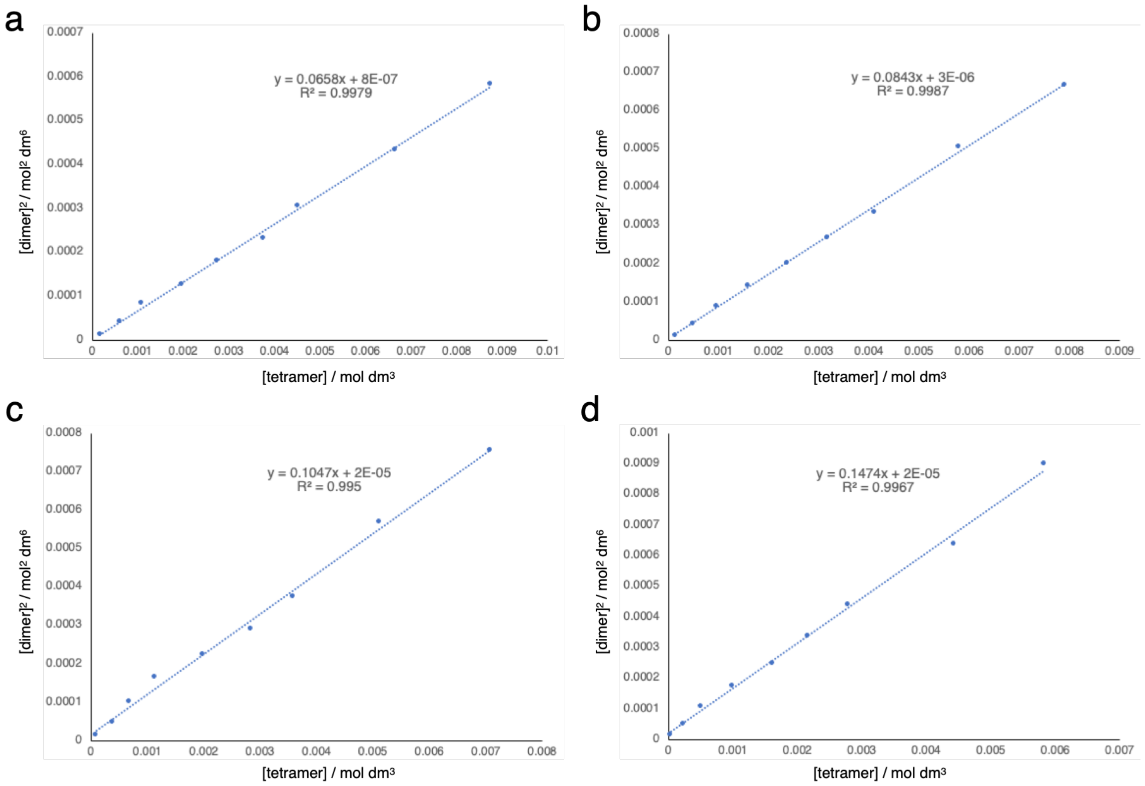

Figure S13: The plot of dinuclear vs. tetranuclear concentration of **2** at a) 45, b) 50, c) 55 and d) 60 °C.

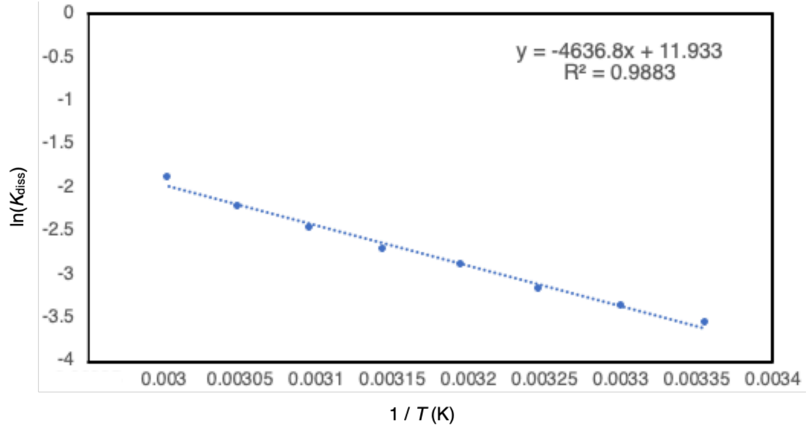

Figure S14: The van't Hoff plot showing the variation of K_{diss} with temperature for the endothermic dissociation process.

Solution-State Thermolysis in Toluene-d_8

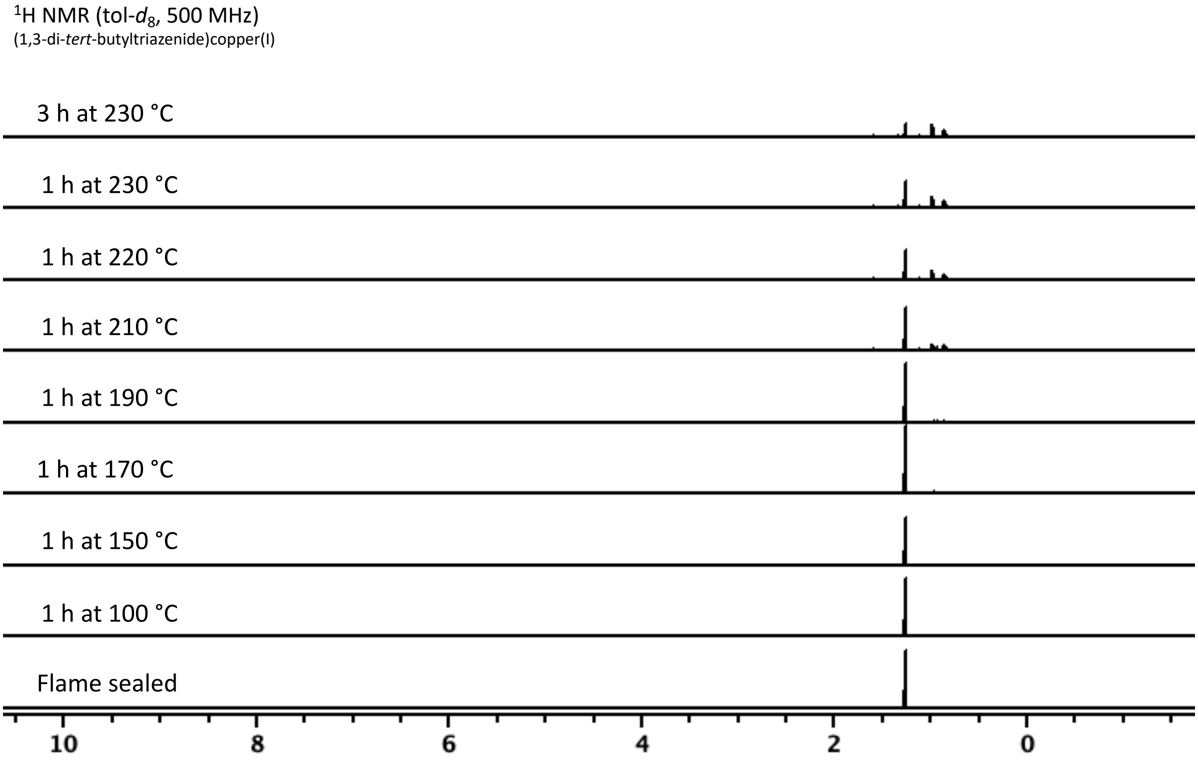

Figure S15: The ^{1}H NMR spectra from solution-state thermolysis of **1** in toluene-d_8.

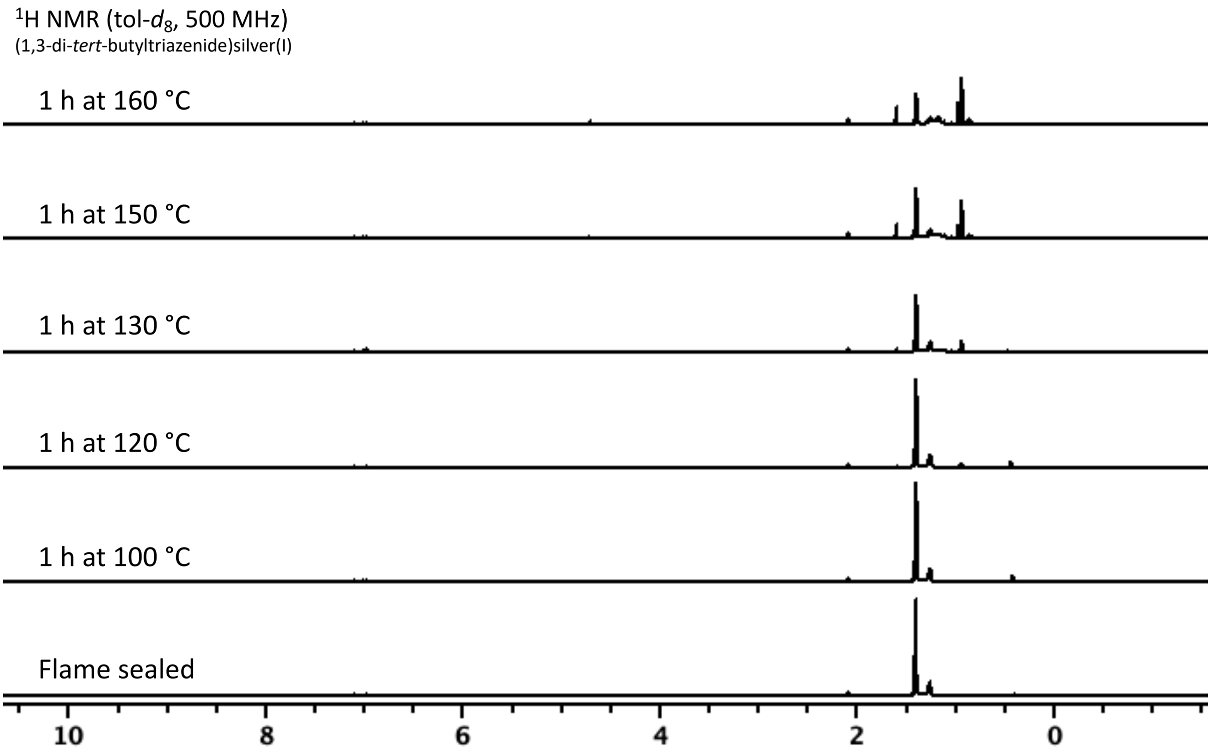

Figure S16: The ^{1}H NMR spectra from solution-state thermolysis of **2** in toluene-d_8.

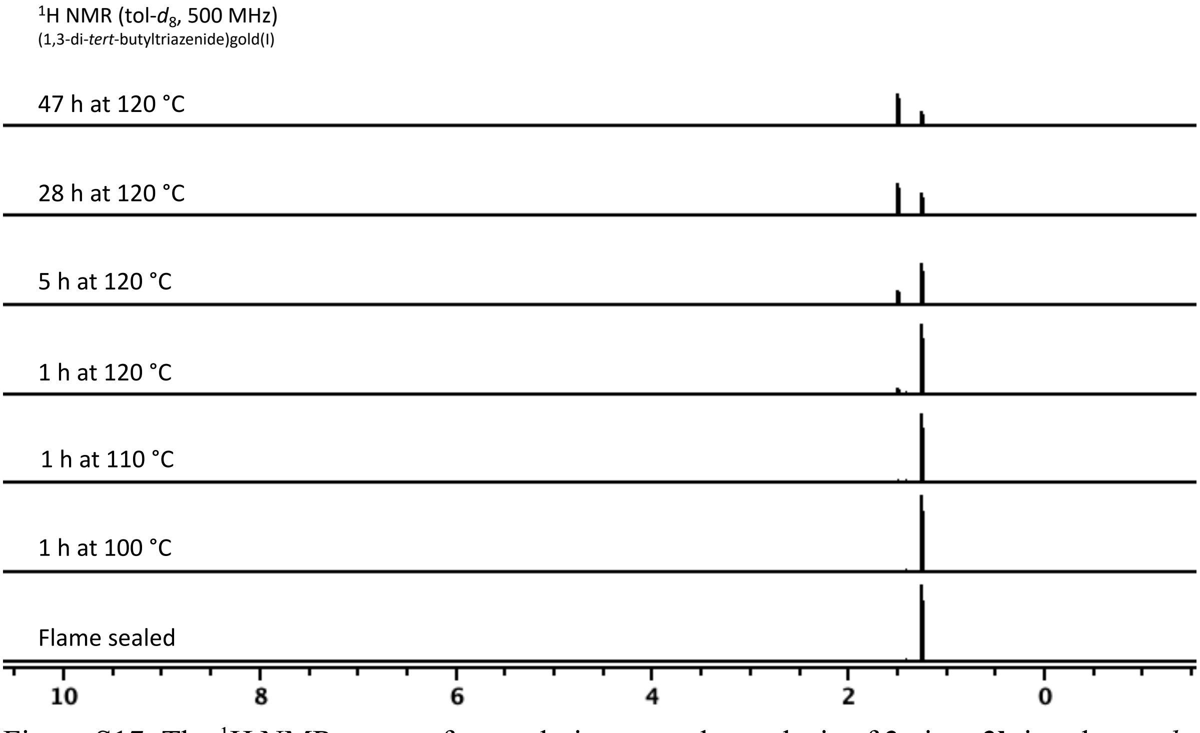

Figure S17: The ^{1}H NMR spectra from solution-state thermolysis of **3a** into **3b** in toluene-d_8.

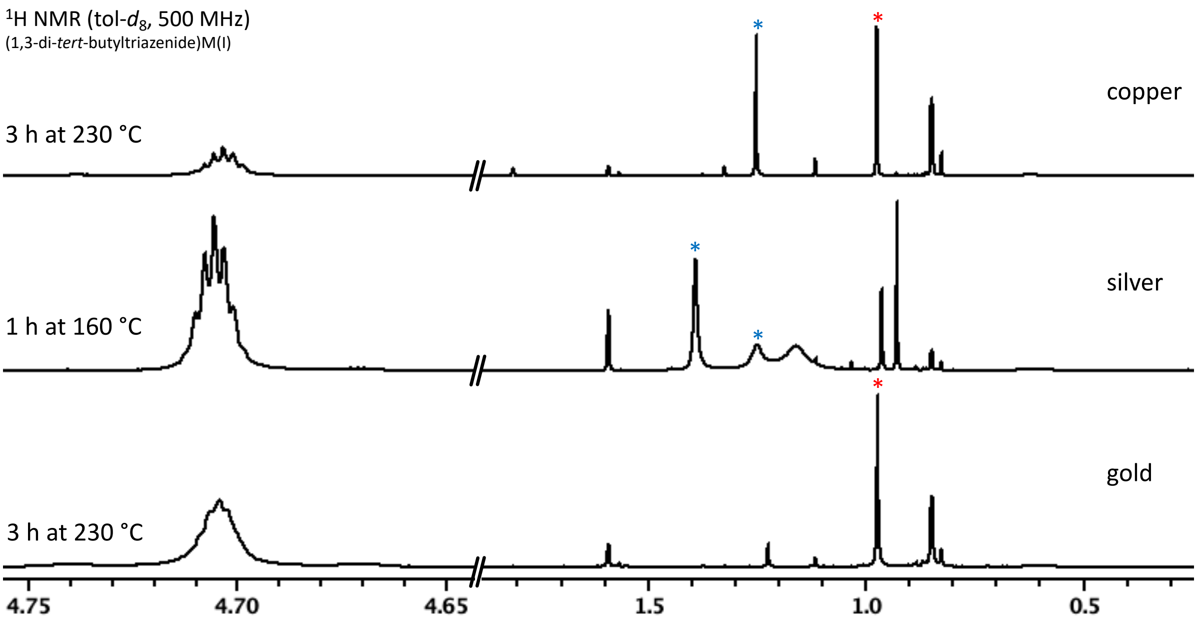

Figure S18: The last ^{1}H NMR spectra recorded (at 25 °C) for **1**, **2** and **3b** during solution-state thermolysis in toluene-d_8. The signals marked by a red asterisk overlap with *tert*-butylamine while the blue asterisks mark the signals of intact **1** and **2**.

Table S1: Integral for solution-state thermolysis experiments of **1**–**3** in reference to the normalized toluene-d_8 peak at 2.08 ppm. The signal for tetranuclear **2** at 1.39 ppm is displayed due to signal overlap of dinuclear **2** at 1.25 ppm and by-products forming above 120 °C.

Temp. (°C)	**1**	**2**	**3a**	**3b**
25	90	34	67	0
100	91	36	66	<1
110	90	35	64	2
120	88	32	60, 15[1]	5, 39[1]
130	88	19	14	40
140	88	23	13	39
150	87	11	11	39
160	88	12	7	41
170	87	—	2	39
180	84	—	2	33
190	82	—	2	20
200	60	—	0	4
210	48	—	0	0
210	34	—	—	—

[1]Compound **3a** was held at 120 °C for 47 hours. The first and second value represents the integral after 1 and 37 hours, respectively.

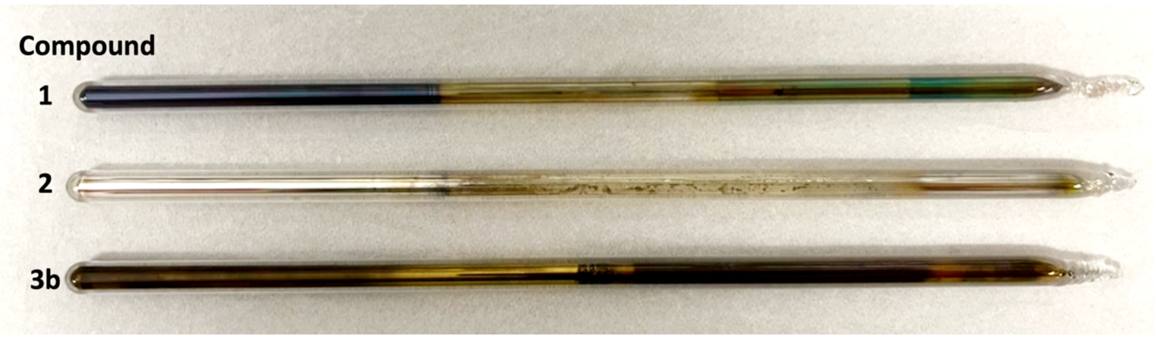

Figure S19: The NMR tubes from the solution-state thermolysis studies of **1-3a**, showing the formation of metallic films inside the tube after decomposition.

Diffusion-Ordered Spectroscopy

Table S2: Diffusion coefficients (in $m^2 s^{-1}$) for the NMR signals of **1**–**3** and the residual solvent signal of benzene.

	Dinuclear	Tetranuclear	Benzene
**1**	7.80E-10	N/A	1.85E-9
**2**	7.54E-10	6.62E-10	1.88E-9
**3a**	7.70E-10	N/A	1.86E-9
**3b**	N/A	5.94E-10	1.84E-9

The diffusion coefficients obtained for residual benzene signals are smaller than found in the literature ($2.1E^{-9}$).[1] The anomalies are most likely not caused by sample convection as convection normally overestimates diffusion coefficients.[2]

Thermogravimetric Analysis-Mass Spectrometry

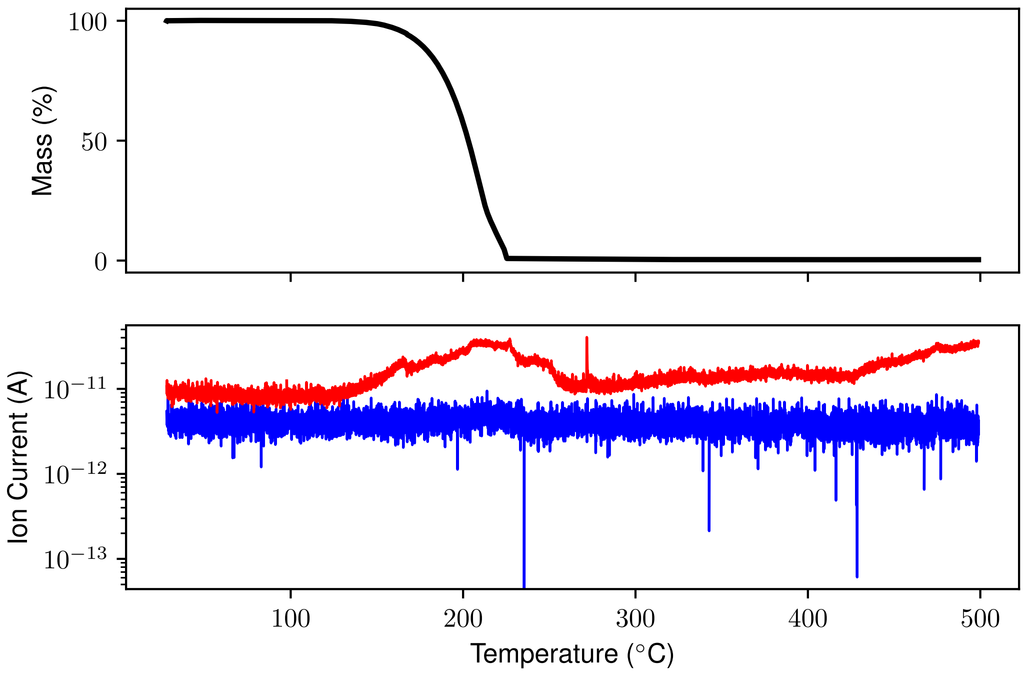

Figure S20: TGA-MS of **1** showing the TGA and MS in the top and bottom graphs, respectively. The MS was monitoring m/z 57 and 99 and are displayed as red and blue line, respectively.

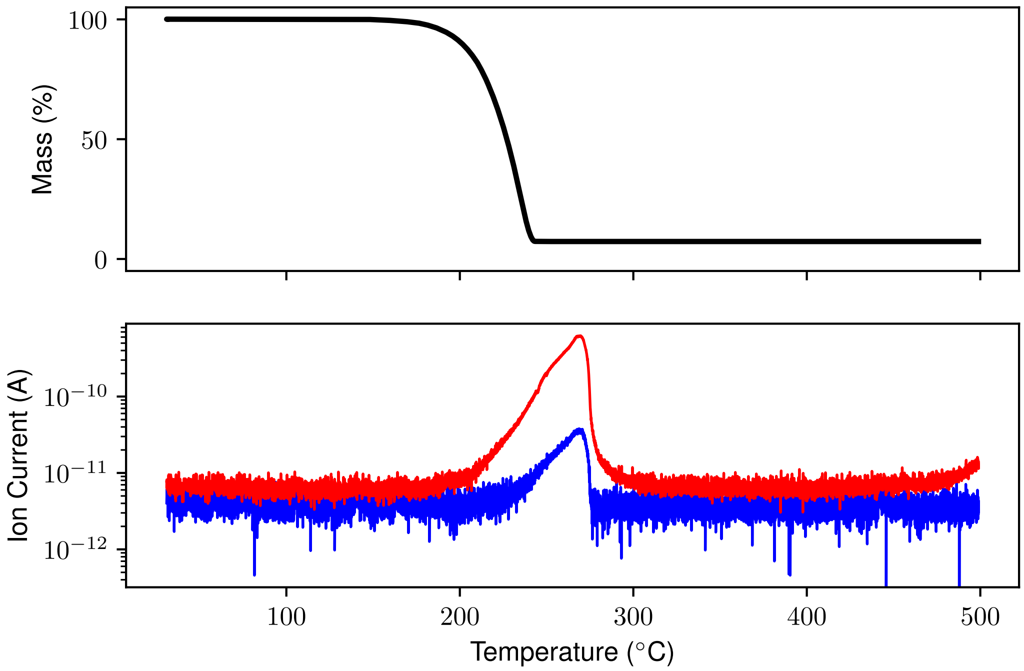

Figure S21: TGA-MS of **2** showing the TGA and MS in the top and bottom graphs, respectively. The MS was monitoring m/z 57 and 99 and are displayed as red and blue line, respectively.

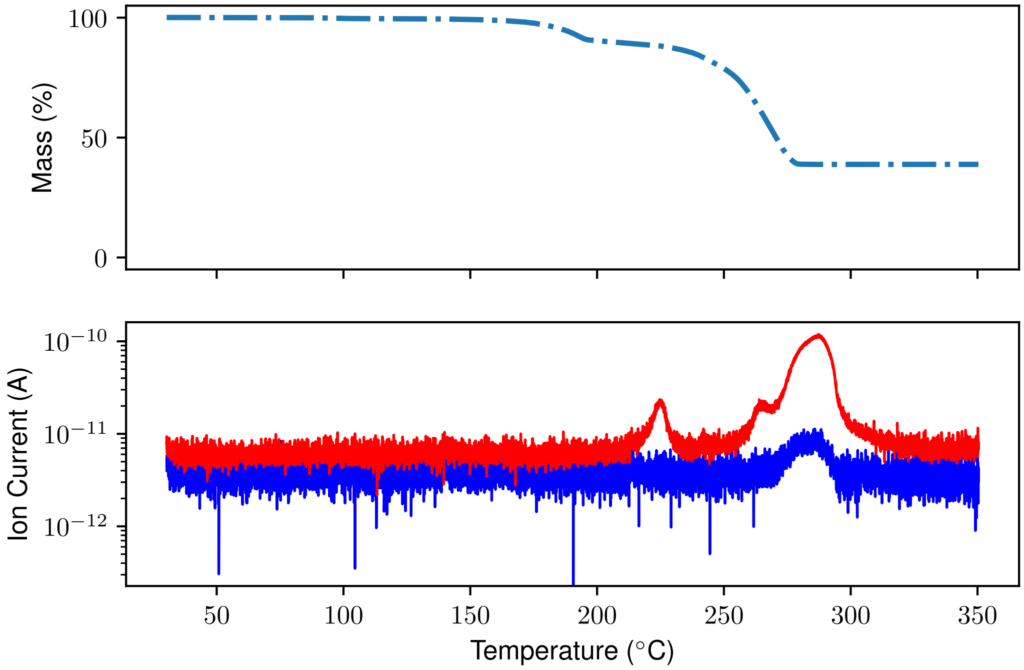

Figure S22: TGA-MS of **2** showing the TGA and MS in the top and bottom graphs, respectively. The MS was monitoring m/z 57 and 99 and are displayed as red and blue line, respectively.

X-ray Crystallography

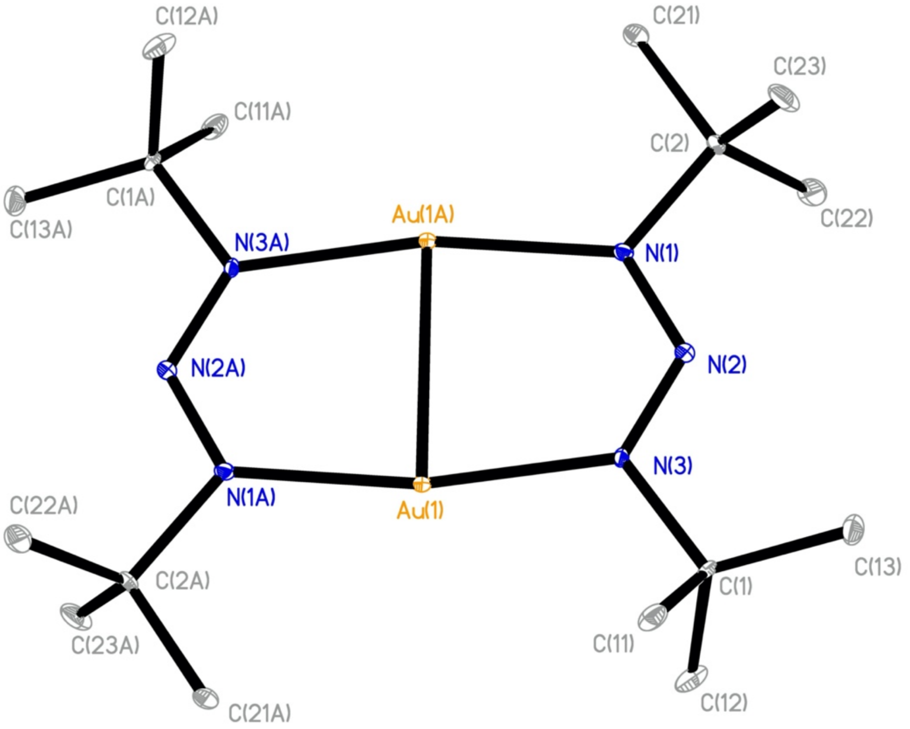

Figure S23: X-ray crystal structure of **3a** which is analogous to the structure **1**.

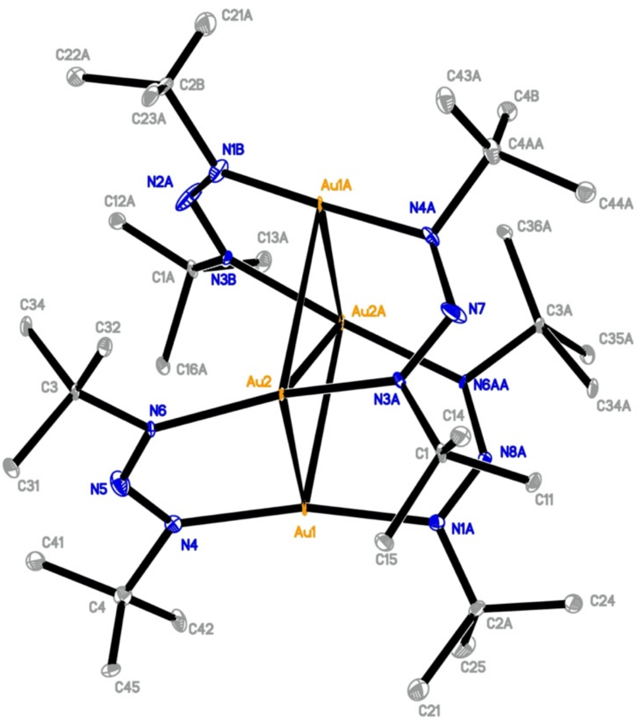

Figure S24: X-ray crystal structure of **3a** which is analogous to the structure of **2**.

Computational Details

Average bond lengths and bond angles for optimized structures of **1–3**

Cartesian coordinates for the dinuclear and tetranuclear structures of **1**–**3** are displayed in Table S18–S44. These structures are visualized for dinuclear and the two tetranuclear structures of **3** in Figure S25–S21. The atom labels presented in the Figure S25–S21 (and tables of cartesian coordinates) are used in visualizing the different resonance structures used in the natural analyses.

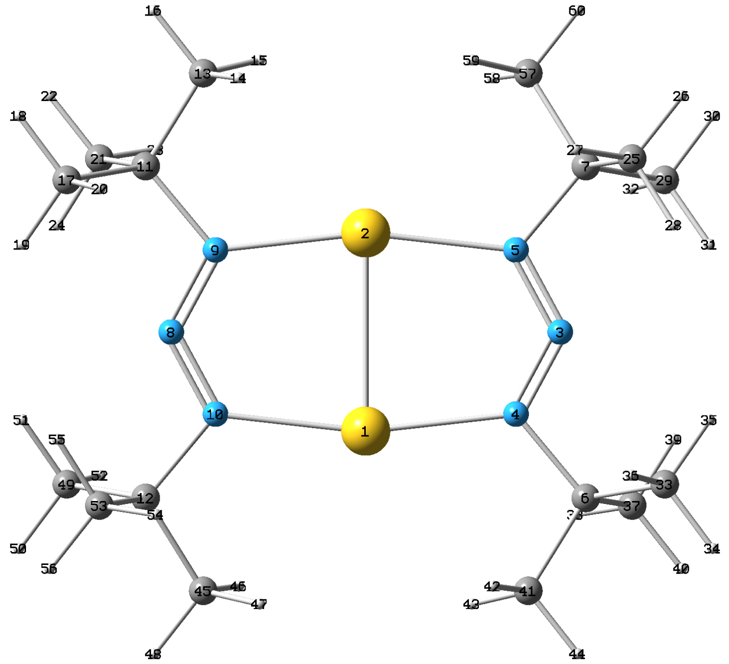

Figure S25: DFT geometry of **3a**.

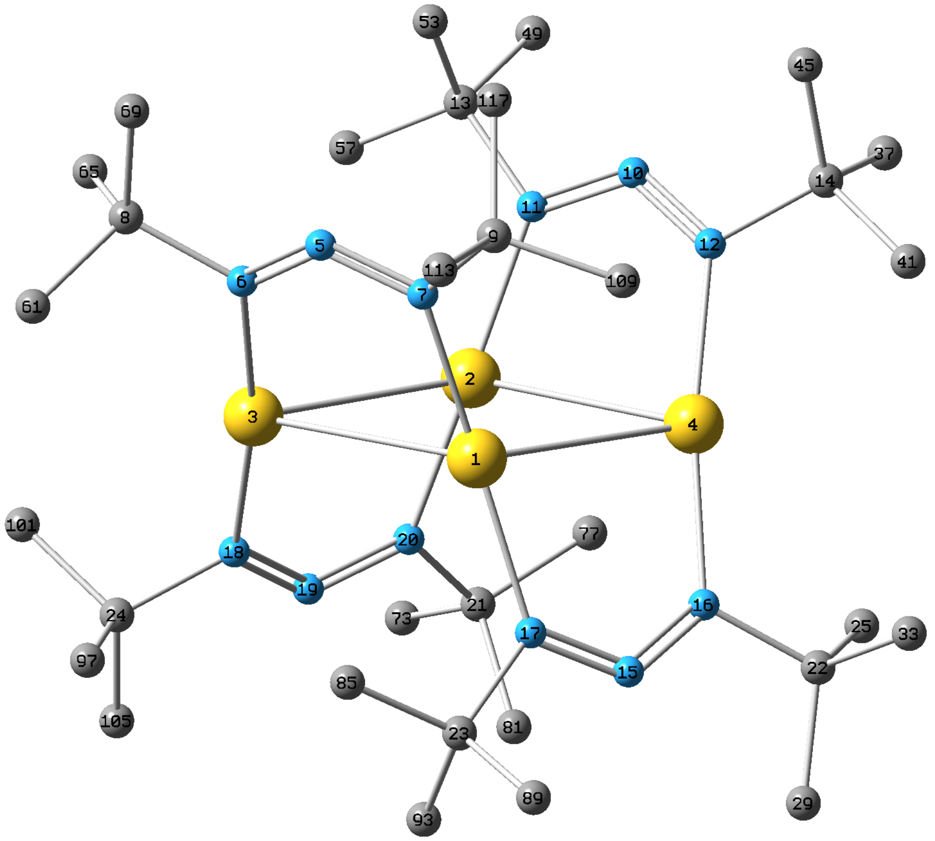

Figure S26: Geometry of rhombic **3b**.

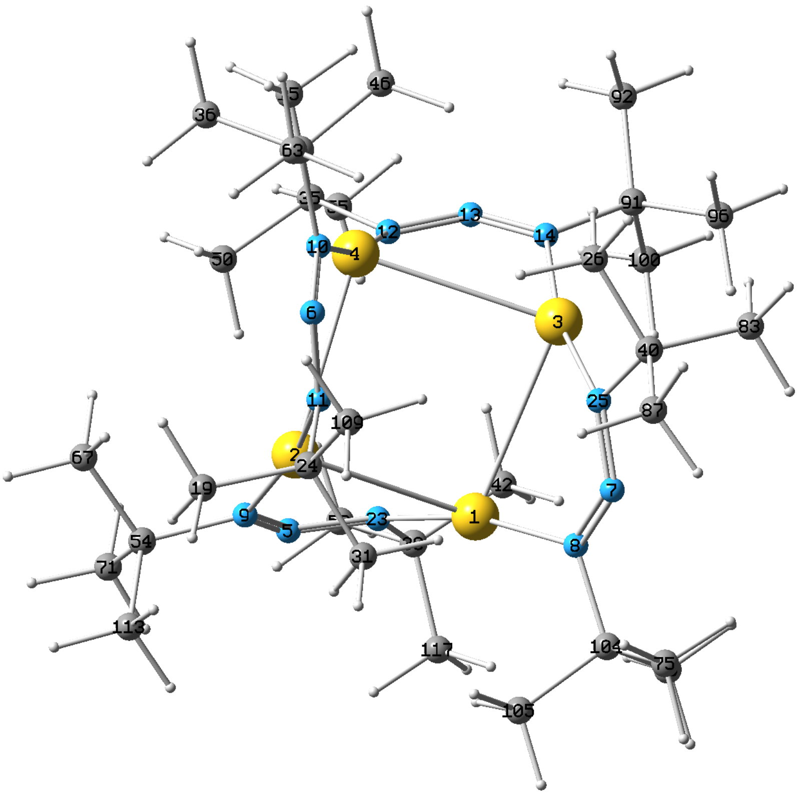

Figure S27: Geometry of buckled square **3b**.

Table S3: Average bond lengths (Å) and angles (°) from DFT geometries of dinuclear **1**–**3**, and the crystal structures of **1** and **3a**.

	**1**		2	**3**	
	DFT	XRC	DFT	DFT	XRC
M···M	2.474	2.443	2.703	2.698	2.656
M–N	1.903	1.883	2.120	2.059	2.050
N–N	1.272	1.292	1.269	1.271	1.289
N–C	1.480		1.480	1.486	
N–M–N	171.71	172.96	166.63	167.10	168.28
N–N–N	119.63	117.73	121.08	123.43	120.50

Table S4: Average bond lengths (Å) and angles (°) from DFT geometries of rhombic **1**–**3** and buckled square **3** with crystal structures for comparison.

	Rhombic					Buckled square	
	**1** DFT	**2** DFT	**2** XRC	**3** DFT	**3** XRC	**3** DFT	**3** XRC
(edge) M···M	2.764	2.950	2.910	3.001	2.958	3.013	2.921
(diag.) M···M	2.894	3.254	3.016	3.452	3.254	4.123	4.043
M–N	1.908	2.117	2.116	2.054	2.066	2.055	2.046
N–N	1.268	1.265	1.297	1.265	1.289	1.265	1.285[1]
N–C	1.485	1.484	—	1.491	—	1.487	—
(acute) M–M–M	63.11	66.94	62.43	70.22	66.75	86.36	87.59
(obtuse) M–M–M	116.84	113.06	117.57	109.77	113.25	88.45	
(acute M) N–M–N	167.79	169.47	162.62	171.16	167.00	168.02	168.52
(obtuse M) N–M–N	156.03	162.15		167.81		168.72	
N–N–N	120.11	121.25	118.51	122.80	119.09	121.77	119.16

Electronic and thermal data for optimized structures of **1**–**3**

Table S5: Electronic- and thermochemical data (Ha) from energy calculations and vibrational analysis at 298.15 K for dinuclear and tetranuclear **1**–**3** optimized in the gas-phase.

SCF	E	ZPE	H	G
Dinuclear **1**	-4240.335986	0.528105	0.559553	0.467187
Rhombic **1**	-8480.673650	1.058806	1.123571	0.963428
Dinuclear **2**	-1253.684087	0.526535	0.559587	0.460154
Rhombic **2**	-2507.378544	1.055703	1.122026	0.953746
Dinuclear **3**	-1231.173459	0.527947	0.560621	0.461098

Rhombic **3**	-2462.371606	1.059212	1.124418	0.959534
Buckled square **3**	-2462.370780	1.058415	1.124121	0.957219

Table S6: Electronic- and thermochemical data (Ha) from energy calculations and vibrational analysis at 298.15 K for dinuclear and tetranuclear **1**–**3** optimized using a polarizable continuum model of benzene.

SCRF-SMD	E	ZPE	H	G
Dinuclear **1**	-4240.353635	0.527462	0.559619	0.466336
Rhombic **1**	-8480.706216	1.060503	1.123533	0.970650
Dinuclear **2**	-1253.698061	0.525455	0.557818	0.459998
Rhombic **2**	-2507.403220	1.054320	1.120795	0.951701
Dinuclear **3**	-1231.187189	0.527570	0.560129	0.462040
Rhombic **3**	-2462.394044	1.057380	1.122874	0.957193
Buckled square **3**	-2462.393932	1.057127	1.122834	0.956584

Table S7: The energy difference, E(rhombic) – 2E(dinuclear) (kJ/mol), for **1**–**3** calculated from data presented in Table S5 and Table S6.

	Gas-phase			SCRF-SMD		
	**1**	**2**	**3**	**1**	**2**	**3**
ΔE	-4.41	-27.2	-64.8	2.77	-18.6	-51.6
ΔZPE	6.82	6.91	8.71	14.6	8.95	5.88
ΔH	11.7	7.49	8.34	11.3	13.5	6.87
ΔG	76.3	87.8	98.0	99.7	83.2	86.9

Natural Bond Orbital Analysis

Dinuclear **1**–**3** gave M–N BOs only slightly higher than 0.50. Dinuclear **1** gave N–N BO slightly below 1.5 while **2** and **3** was slightly above. The N–C BO are below 1.0 and decrease in the order **1** ~ **2** > **3** (Table S8). The NRT calculated M–M BO was 0.

Table S8: Natural resonance theory bond orders for dinuclear **1**–**3**.

	**1**	**2**	**3**
M–N	0.530	0.535	0.532
N–N	1.491	1.503	1.516
N–C	0.962	0.960	0.952

Table S9: Average NBO charges (au) of atoms and groups of atoms for the optimized structures of **1**–**3**.

	Dinuclear			Rhombic			Square
	**1**	**2**	**3**	**1**	**2**	**3**	**3**
M	0.655	0.491	0.627	0.635	0.678	0.489	0.487
N_central	0.016	0.014	0.012	0.008	0.017	0.013	0.014
N_terminal	-0.488	-0.423	-0.475	-0.469	-0.505	-0.424	-0.422
C_tert	0.097	0.103	0.096	0.094	0.100	0.107	0.105
C_prim	-0.630	-0.632	-0.630	-0.630	-0.631	-0.633	-0.631
R-groups	0.153	0.170	0.155	0.148	0.158	0.172	0.172
N_3	-0.961	-0.831	-0.937	-0.931	-0.993	-0.834	-0.830
Ligand	-0.655	-0.491	-0.627	-0.635	-0.678	-0.489	-0.487

The heavier metal centers of **3** differ in s and d natural atomic orbital (NAO) occupancy from **1** and **2** due to more pronounced relativistic effects.[6] Relativistic d orbital expansion and s orbital contraction in **3** redistributes electrons from valence d- to valence s-orbitals (Table S10 and Table S11). Furthermore, the size difference of the valence s- and d orbitals increase in the order **3** < **2** < **1** (Figure S28).

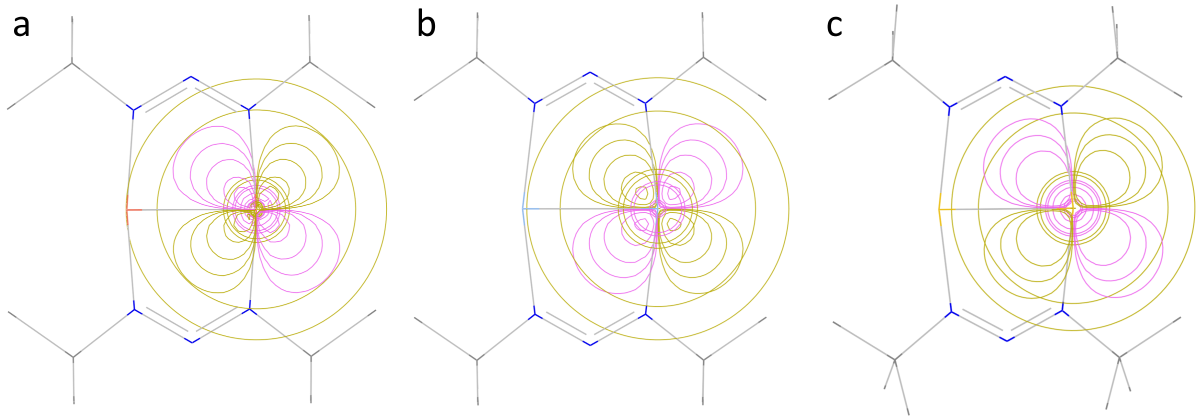

Figure S28: Contour plots of pre-orthogonalized metal center valence s- and d-natural atomic orbitals of dinuclear **1** (a), **2** (b), and **3** (c).

The better size-match allows **3** to form sd-hybrids with a more even mixing of s and d NAOs than **1** and **2** when forming NBOs. In fact, from the NBO perspective, structures of **3** mainly deviate in s and d NAO occupancy from **1** and **2** due to hybridization of a metal-center lone pair, n^{sd}_M. The n^{sd}_M has ~17% s character for **3**, and ~7% for **1** and **2**. More valence d electrons are therefore promoted to the valence s orbitals to form these NBOs for **3** than for **1** and **2**. The larger electron transfer in **3** explains their lower and higher valence d and s occupancies, respectively.

Table S10: The metal center NAO occupancy (in number of electrons) for dinuclear and both conformations of tetranuclear **1**–**3**.

Dinuclear	Rhombic	Square

	1	2	3	1	2	3	3
s	0.527	0.513	0.831	0.498	0.516	0.837	0.840
p	0.007	0.008	0.009	0.007	0.015	0.012	0.010
d	9.805	9.841	9.662	9.805	9.838	9.655	9.657

Table S11: Average natural atomic orbital occupancies for optimized structures of **1**–**3**. For the p-orbitals, the average is on the sum of the valence and Rydberg orbitals.

Orbital	Dinuclear **1**	Rhombic **1**	Dinuclear **2**	Rhombic **2**	Dinuclear **3**	Rhombic **3**	Buckled square **3**
val s	0.5272	0.4976	0.5128	0.5164	0.8311	0.8375	0.8401
val+ryd p	0.0068	0.0071	0.0081	0.0146	0.0087	0.0119	0.0103
val dxy	1.9881	1.9701	1.9892	1.9747	1.9793	1.9473	1.9823
val dxz	1.9786	1.9936	1.9863	1.9951	1.9712	1.9917	1.9080
val dyz	1.9987	1.9536	1.9988	1.9587	1.9981	1.9106	1.9096
val dx2y2	1.8775	1.9164	1.9009	1.9322	1.7872	1.8542	1.9895
val dz2	1.9616	1.9714	1.9654	1.9771	1.9264	1.9508	1.8675

Natural Lewis Structures

Four candidate natural Lewis structures were considered for dinuclear **1**–**3** to study the NBOs and second order perturbation theory analysis. The Lewis structures were of similar quality and differed only in their M–N bonding. However, NLS-1 and 4 gave σ_{MN} without contribution from the metal-center NAH. NLS-2 was therefore used for comparing the σ_{MN} and σ^*_{MN} of dinuclear **1**–**3**. NLS-0 was used, together with its rhombic analog (NLS-R0), to compare bonding interactions between dinuclear and rhombic **1**–**3** through second order perturbation theory analysis.

NLS-2 showed highly polarized σ_{MN} with ~9% contribution from the metal center natural atomic hybrids (NAH) for dinuclear **1** and **2**, and ~15% for **3**. The M NAHs for **1** and **2** had ~90% s-character, and ~80% s-character for **3**. Furthermore, second-order perturbation theory analysis (donor-acceptor interactions) indicated the presence of 3-center 4-electron N–M–N bonds (3c/4e). The 3c/4e bonds appeared as highly stabilizing $n_N \rightarrow \sigma^*_{MN}$ donor-acceptor interactions, (~77, 80, and 150 kcal/mol for **1**, **2**, and **3**, respectively). The N NAH p-character was slightly higher for **3** than for **1** and **2** which is consistent with Bent's rule.[7] The σ^*_{MN} occupancies increased in the order **1** < **2** << **3**, where the difference between **1** and **2** was ~0.01 and **3** having ~0.08 e more than **1** and **2**.

The difference between NLS-2 and NLS-0 can roughly be understood as a reconstruction of σ_{MN} and σ^*_{MN} of NLS-2 into an occupied and a vacant lone pair on N ($n1_N$) and M (n^*_M),

respectively. These lone pairs are essentially constructed from the NAH of σ_{MN}. Consequently, the differences in hybridization in the NAHs of σ_{MN} in NLS-2 are also seen in the hybridization of $n1_N$ and n^*_M in NLS-0. For example, in NLS-0, the M–N interactions appear as highly stabilizing $n1_N \rightarrow n^*_M$ donor-acceptor interactions, where $n1_N$ and n^*_M are the reconstructed σ_{MN} and σ^*_{MN}.

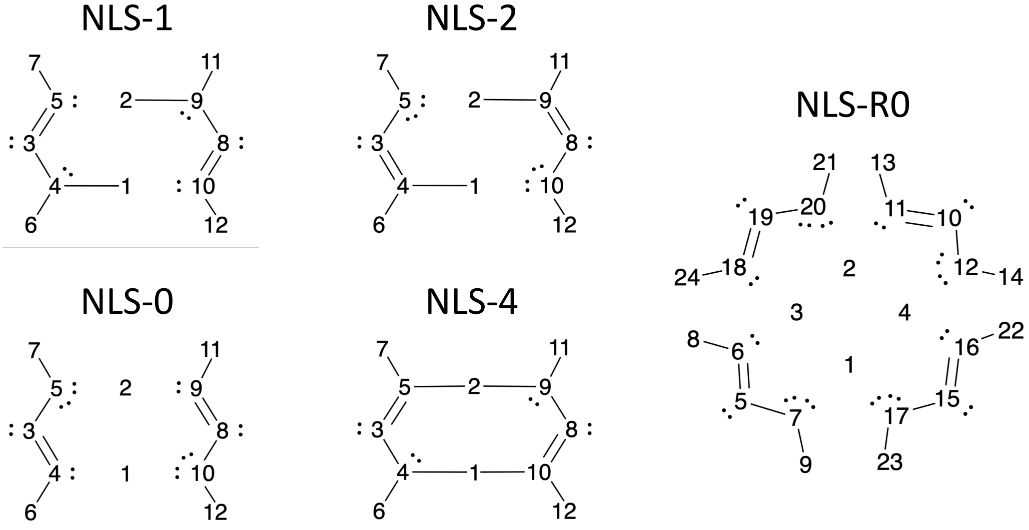

Figure S29: Lewis diagrams of the natural Lewis structures used for **1**–**3** in constructing NBOs. The numbers refer to the center number of the structures and can be found in Table S18–Table S31. Only the N–C bonds are drawn for the *tert*-butyl groups as they are constructed as expected (the tertiary C with one C–N- and three C–C bonds, and each primary C with one C–C- and three C–H bonds).

Table S12: Lewis and non-Lewis contribution (%) of the total electron density for dinuclear and rhombic **1**–**3** constructed from different natural Lewis structures.

		Dinuclear **1**			Rhombic **1**
	NLS-0	NLS-1	NLS-2	NLS-3	NLS-0
Lewis_Val	98.10	98.30	98.31	98.31	98.07
Lewis_tot	98.75	98.88	98.89	98.89	98.73
Non-Lewis_Val	1.13	1.00	0.99	0.99	1.13
Non-Lewis_Ryd	0.12	0.12	0.12	0.12	0.14
Non-Lewis_tot	1.25	1.12	1.11	1.11	1.27
		Dinuclear **2**			Rhombic **2**
	NLS-0	NLS-1	NLS-2	NLS-3	NLS-0
Lewis_Val	98.08	98.09	98.26	98.29	98.04
Lewis_tot	98.91	98.91	99.01	99.03	98.89
Non-Lewis_Val	0.98	0.68	0.88	0.87	1.00
Non-Lewis_Ryd	0.11	0.40	0.11	0.11	0.11
Non-Lewis_tot	1.09	1.09	0.99	0.97	1.11
		Dinuclear **3**			Rhombic **3**

	NLS-0	NLS-1	NLS-2	NLS-3	NLS-0
Lewis_Val	97.83	97.83	98.14	98.16	97.79
Lewis_tot	99.01	99.01	99.15	99.16	98.99
Non-Lewis_Val	0.91	0.68	0.76	0.76	0.92
Non-Lewis_Ryd	0.09	0.31	0.09	0.09	0.09
Non-Lewis_tot	0.99	0.99	0.85	0.85	1.01

Table S13: Average NBO energies (Ha) for dinuclear and rhombic **1**–**3** in NLS-0.

	Dinuclear **1**	Rhombic **1**	Dinuclear **2**	Rhombic **2**	Dinuclear **3**	Rhombic **3**
$n1_M$	-0.37646	-0.37893	-0.43222	-0.43618	-0.40328	-0.40244
$n2_M$	-0.37571	-0.37846	-0.43315	-0.43621	-0.40610	-0.40157
$n3_M$	-0.37594	-0.37684	-0.43153	-0.43377	-0.41442	-0.40822
$n4_M$	-0.37152	-0.37448	-0.42813	-0.43176	-0.38021	-0.37592
$n^{sd}{}_M$	-0.36659	-0.36535	-0.41193	-0.40170	-0.40901	-0.40368
$n*_M$	0.08724	0.10453	0.17111	0.23730	0.07498	0.05451
$n1_{N\_term}$	-0.49002	-0.49346	-0.47106	-0.47325	-0.46568	-0.46379
$n2_{N\_term}$	-0.27439	-0.27205	-0.26811	-0.26737	-0.28652	-0.28185
$n1_{N\_cent}$	-0.46710	-0.45975	-0.45974	-0.45544	-0.46356	-0.45738
σ_{NN}	-0.95068	-0.95121	-0.95037	-0.95277	-0.96607	-0.96495
π_{NN}	-0.47580	-0.47826	-0.47327	-0.47749	-0.48823	-0.48909
σ_{NC}	-0.73945	-0.73663	-0.73511	-0.73489	-0.74435	-0.74056
$\sigma*_{NN}$	0.63499	0.63925	0.64422	0.64876	0.63375	0.64465
$\pi*_{NN}$	0.00751	0.01255	0.01309	0.01672	-0.00127	0.00656
$\sigma*_{NC}$	0.43268	0.42789	0.43585	0.43193	0.42100	0.41840

Table S14: Average NBO occupancies (e) for dinuclear and rhombic **1**–**3** in NLS-0.

	Dinuclear **1**	Rhombic **1**	Dinuclear **2**	Rhombic **2**	Dinuclear **3**	Rhombic **3**
$n1_M$	1.999	1.998	1.999	1.998	1.999	1.998
$n2_M$	1.999	1.997	1.999	1.998	1.998	1.996
$n3_M$	1.988	1.986	1.989	1.989	1.979	1.978
$n4_M$	1.979	1.978	1.986	1.985	1.972	1.973
$n^{sd}{}_M$	1.980	1.964	1.982	1.973	1.979	1.968
$n*_M$	0.390	0.383	0.401	0.416	0.570	0.583
$n1_{N\_term}$	1.808	1.826	1.809	1.812	1.725	1.726
$n2_{N\_term}$	1.488	1.490	1.482	1.487	1.490	1.493
$n1_{N\_cent}$	1.930	1.918	1.918	1.907	1.916	1.909
σ_{NN}	1.988	1.988	1.988	1.988	1.989	1.988
π_{NN}	1.975	1.973	1.975	1.974	1.976	1.975
σ_{NC}	1.978	1.978	1.977	1.977	1.977	1.977
$\sigma*_{NN}$	0.018	0.020	0.018	0.019	0.020	0.023
$\pi*_{NN}$	0.508	0.507	0.506	0.502	0.515	0.513

σ^*_{NC}	0.045	0.045	0.043	0.044	0.048	0.048

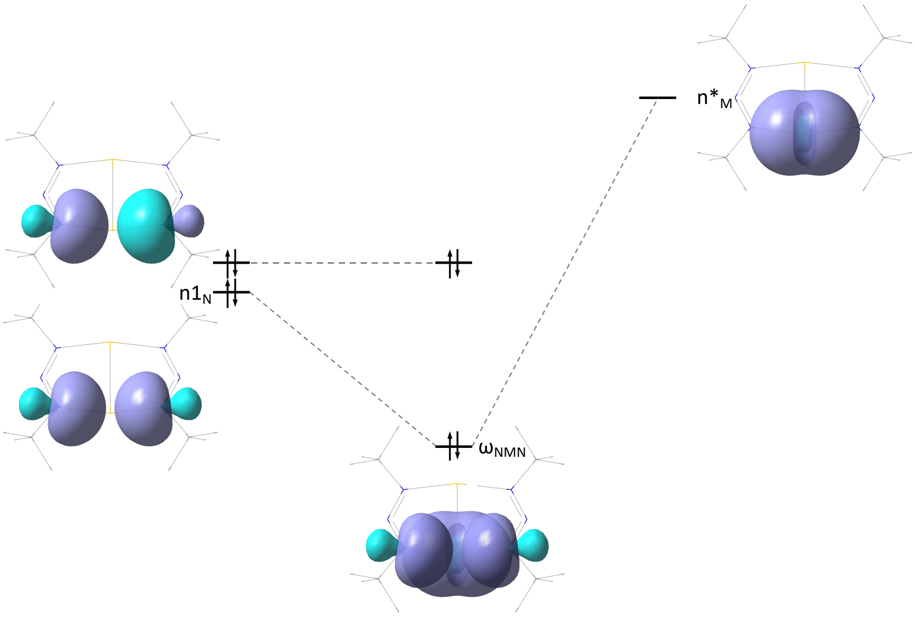

Figure S30: Conceptual illustration of the donor-acceptor interactions making up the 3c/4e bond (ω_{NMN}) in dinuclear **3,** constructed from two symmetry adapted linear combinations of $n1_N$ NBOs and a n^*_M NBOs in NLS-0.

Second Order Perturbation Theory Analysis

The donor-acceptor interactions of dinuclear and rhombic **1**–**3** fall in four categories: metal-metal (M–M), ligand to metal (L→M), metal to ligand (M→L) and ligand-ligand (L–L). Most donor-acceptor interactions of dinuclear **1**–**3** have higher stabilization energies ($E^{(2)}$) than the analogous rhombic interactions due to better orbital overlap. The rhombic structures, however, have more neighbors to engage in donor-acceptor interactions with. For example, each dinuclear metal center has one neighboring metal center, limiting the M–M interactions to orbitals along that direction. Meanwhile, each metal center in the rhombic structures have two neighboring metal centers (excluding the diagonal neighbors) allowing them to use a larger set of orbitals for M–M donor-acceptor interactions.

Although the number of geminal interactions do not increase in the M–L- and L–L categories, there is greater potential in the rhombic structures for long-range donor-acceptor interactions. The dinuclear-tetranuclear preference comes down to how much individual interactions degrade when going from dinuclear to tetranuclear, in relation to how many additional interactions are gained for a tetranuclear over two dinuclear. Mainly, degradation of the $n1_N \rightarrow n^*_M$ favors the dinuclear structures while donation into Rydberg levels favor the rhombic

structures. The tetranuclear-dinuclear energy difference for $n1_N \rightarrow n^*_M$ increase in the order **3** < **2** < **1**. M–L interactions involving Rydberg acceptor levels are weak for structures of **1**, and rhombic **1** do not compensate for the weaker $n1_N \rightarrow n^*_M$. Meanwhile, the interactions in **2** and **3** favoring the rhombic structures are stronger than **1**, which may explain their preference to be rhombic (Table S16). For many impactful interactions studied, dinuclear **2** showed smaller degradation than **1** and **3** when going from dinuclear to rhombic, e.g. $n^{sd}_M \rightarrow n^*_M$ (Table S17).

Table S15: The sum of donor-acceptor stabilization energy $E^{(2)}$ (kcal/mol) of M–M and L → M with Rydberg acceptors for dinuclear and rhombic **1**–**3** in NLS-0. The $E^{(2)}$ for dinuclear interactions are multiplied by two.

	M–M	L → M	$n_N \rightarrow RY_M$ [V + R]	$n_N \rightarrow RY_M$ [G]
Rhombic **1**	4.38	25.8	< 0.01	18.2
Dinuclear **1**	5.28	19.5	< 0.01	11.2
Rhombic **2**	330	632	237	380
Dinuclear **2**	99.1	629	154	457
Rhombic **3**	506	1308	381	810
Dinuclear **3**	314	1309	305	930

Table S16: The difference $\Delta E^{(2)}$ (Rhombic – 2*Dinuclear) in $E^{(2)}$ (kcal/mol) for geminal, vicinal and remote donor-acceptor interactions of **1**–**3** involving Rydberg acceptors.

	M–RY_M	L → RY_M	$n_N \rightarrow RY_M$ [V + R]	$n_N \rightarrow RY_M$ [G]
**1**	-0.90	6.30	0	6.94
**2**	231	3.20	82.3	-77.0
**3**	192	-0.49	76.2	-120

Table S17: Second order perturbation theory analysis of individual M–M, M → L, and L → M donor-acceptor interactions of dinuclear and rhombic **1**–**3** in NLS-0. $E^{(2)}$ is the stabilization energy for the interaction (kcal/mol), ΔE_{ji} is the donor-acceptor orbital energy difference (Ha), and F_{ji} is the donor-acceptor orbital overlap integral (au).

	$n^{sd}_M \rightarrow n^*_M$ [Geminal] (non-diagonal)			$n1_N \rightarrow n^*_M$ [Geminal]		
	$E^{(2)}$	ΔE_{ji}	ΔF_{ji}	$E^{(2)}$	ΔE_{ji}	ΔF_{ji}
Rhombic **1**	2.56	0.470	0.031	68.7	0.596	0.181
Dinuclear **1**	4.16	0.450	0.039	87.0	0.580	0.200
Rhombic **2**	7.93	0.639	0.063	75.1	0.711	0.206
Dinuclear **2**	7.16	0.580	0.058	87.5	0.640	0.212
Rhombic **3**	7.78	0.431	0.052	176	0.519	0.270
Dinuclear **3**	14.7	0.460	0.073	183	0.540	0.281
	$n_M \rightarrow \sigma^*_{NN}$ [Geminal]			$n1_N \rightarrow RY1_N$ [Geminal]		
Rhombic **1**	1.18	1.02	0.031	0.125	1.56	0.013

Dinuclear **1**	1.38	1.01	0.033	0.080	1.53	0.010
Rhombic **2**	1.10	1.08	0.031	4.21	2.08	0.084
Dinuclear **2**	1.00	1.08	0.029	3.73	1.82	0.074
Rhombic **3**	2.10	1.05	0.042	11.4	2.93	0.164
Dinuclear **3**	2.10	1.05	0.042	11.4	2.54	0.152
	$n^{sd}_M \rightarrow \pi^*_{NN}$ [Geminal]			$n1_N \rightarrow RY3_N$ [Geminal]		
Rhombic **1**	3.69	0.390	0.034	N/A	N/A	N/A
Dinuclear **1**	4.45	0.380	0.037	0.050	1.79	0.009
Rhombic **2**	2.79	0.450	0.032	3.68	2.59	0.087
Dinuclear **2**	3.12	0.440	0.033	4.56	2.51	0.095
Rhombic **3**	5.92	0.410	0.044	10.9	4.21	0.192
Dinuclear **3**	6.31	0.410	0.045	11.4	3.08	0.167
	$n^{sd}_M \rightarrow RY1_N$ [Geminal]			$n^{sd}_M \rightarrow RY3_N$ [Geminal]		
Rhombic **1**	1.46	1.43	0.041	1.31	1.66	0.041
Dinuclear **1**	1.45	1.40	0.040	0.88	1.67	0.034
Rhombic **2**	12.3	2.03	0.141	11.1	2.52	0.149
Dinuclear **2**	5.94	1.76	0.091	4.97	2.45	0.098
Rhombic **3**	13.7	2.84	0.176	14.3	4.12	0.215
Dinuclear **3**	12.9	2.46	0.159	9.29	3.00	0.149

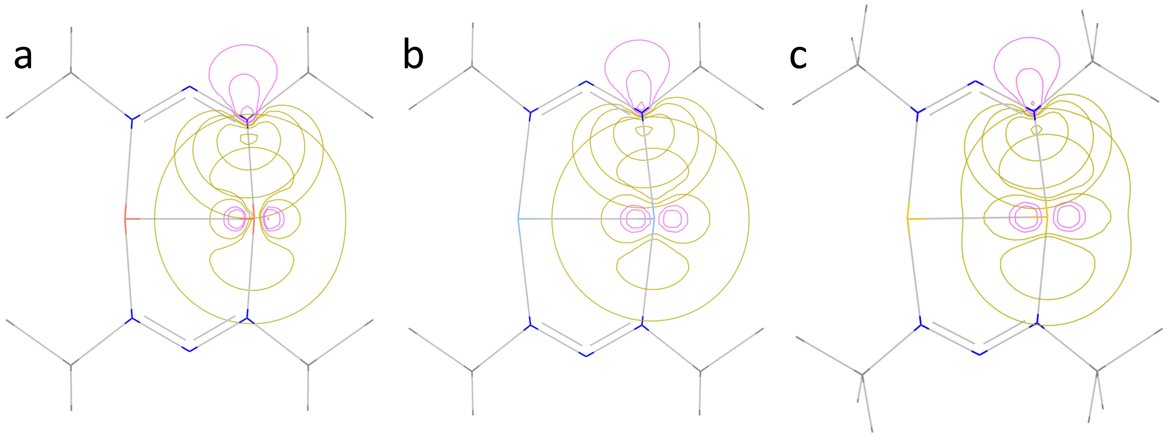

Figure S31: Contour plots of the $n1_N \rightarrow n^*_M$ donor-acceptor interaction of dinuclear a) **1**, b) **2**, and c) **3** in NLS-0.

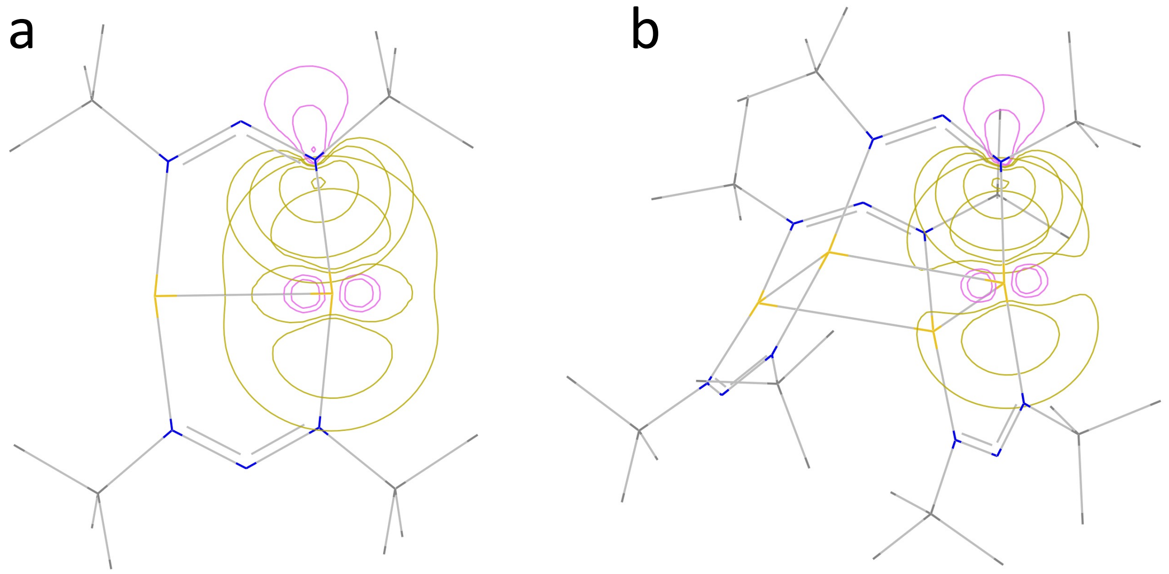

Figure S32: Contour plots of $n1_N \rightarrow n^*_M$ donor-acceptor interaction for a) dinuclear and b) rhombic **3** in NLS-0.

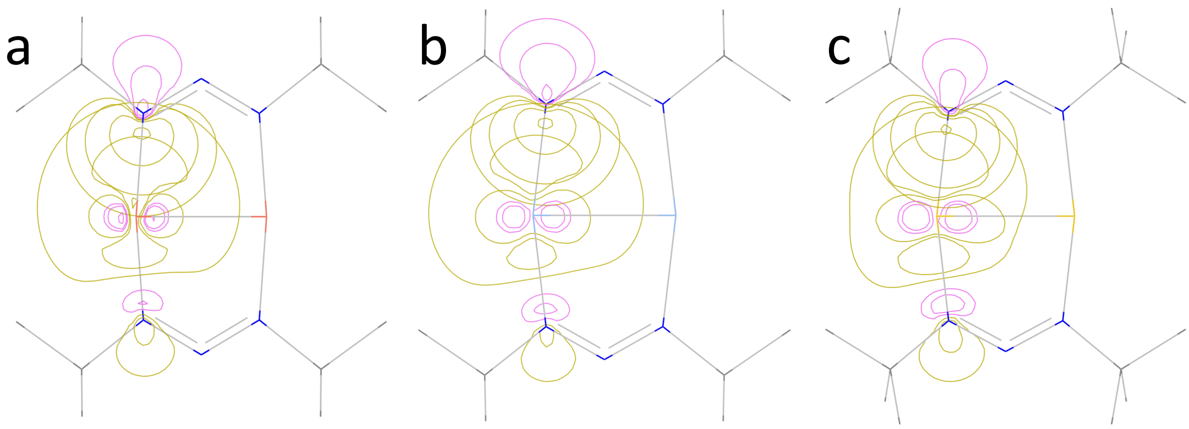

Figure S33: Contour plots of $n1_N \rightarrow \sigma^*_{MN}$ donor-acceptor interaction for dinuclear a) **1**, b) **2**, and c) **3** in NLS-2. This interaction is associated with the 3c/4e bonds and provide a stabilization of 77, 83, and 152 kcal/mol for **1**, **2**, and **3**, respectively.

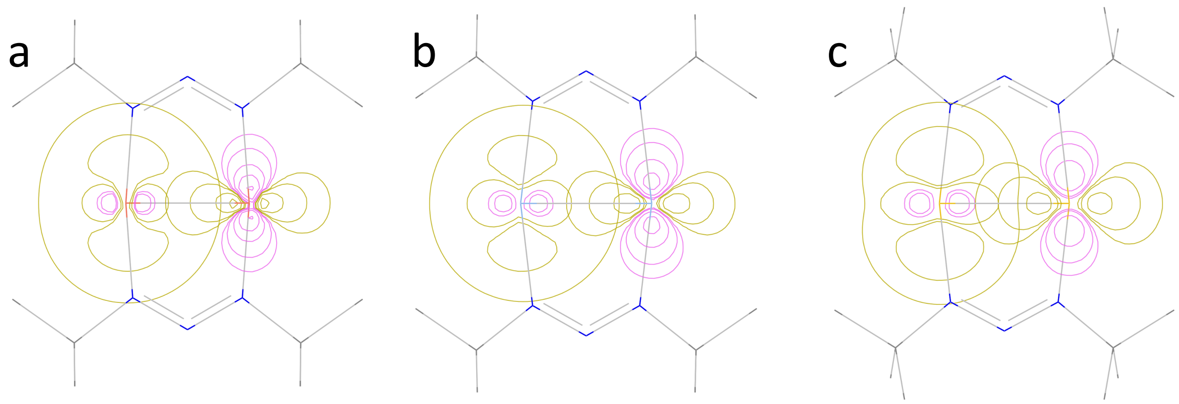

Figure S34: Contour plots of $n^{sd}_M \rightarrow n^*_M$ donor-acceptor interaction for dinuclear a) **1**, b) **2**, and c) **3** in NLS-0.

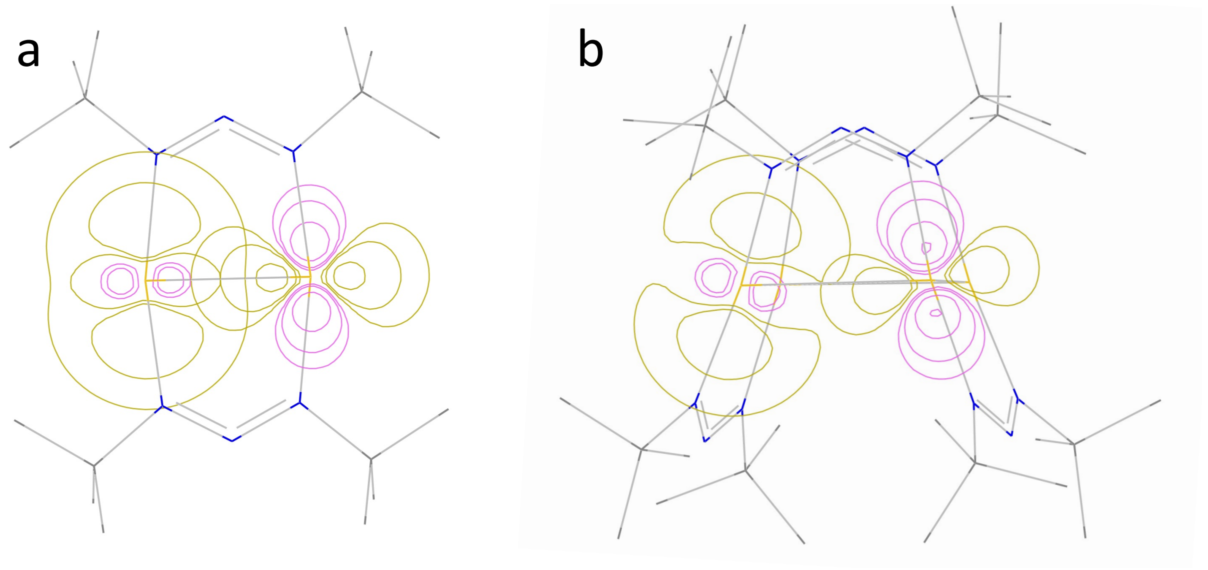

Figure S35: Contour plots of n^{sd}_M → n^*_M donor-acceptor interaction for dinuclear (a) and rhombic (b) **3** in NLS-0.

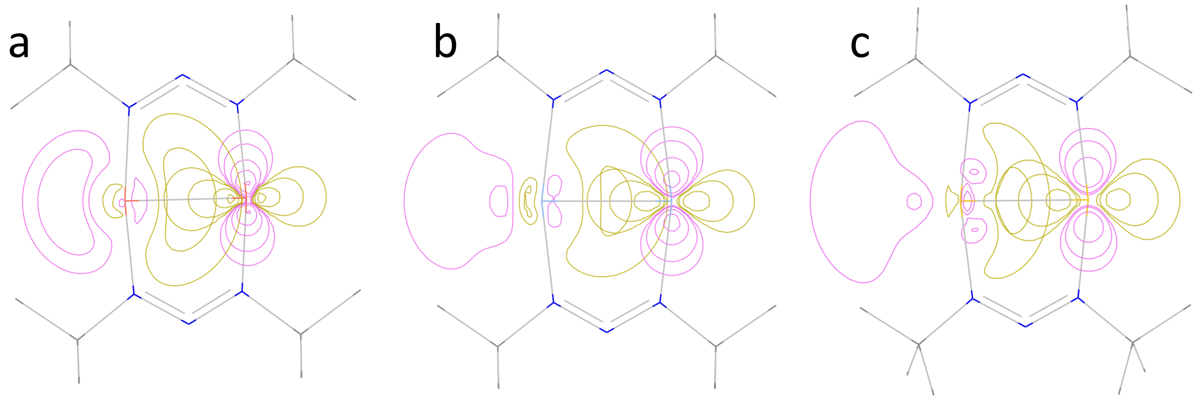

Figure S36: Contour plots of n^{sd}_M → $RY3_M$ donor-acceptor interaction for dinuclear a) **1**, b) **2**, and c) **3** in NLS-0. The acceptor orbital (left metal center) for **3** has a significantly difference in shape compared to **1** and **2**.

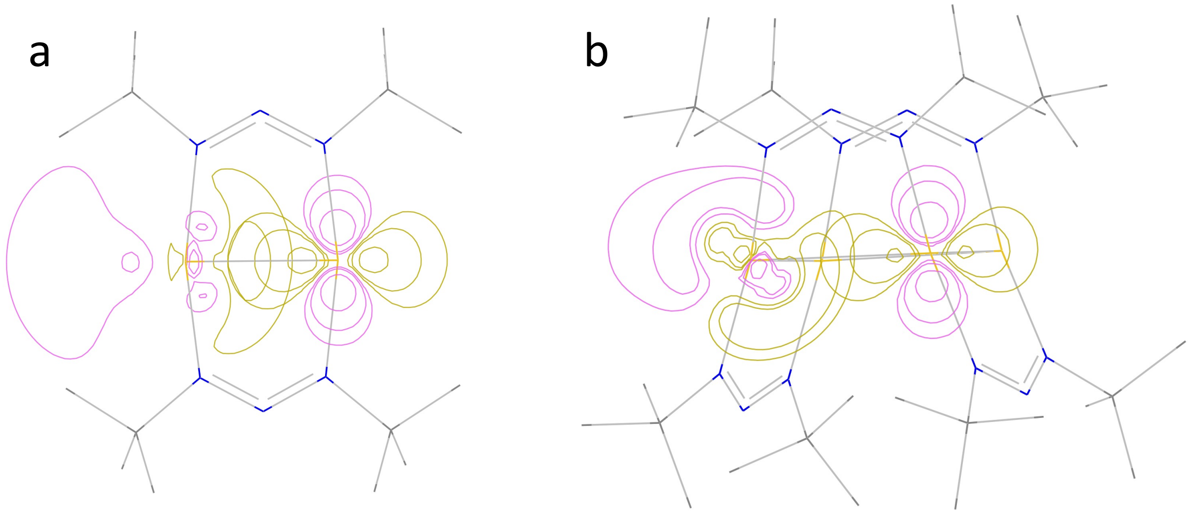

Figure S37: The $n^{sd}_{M} \rightarrow RY3_{M}$ interaction for a) dinuclear and b) rhombic **3** in NLS-0.

Cartesian coordinates for optimized geometries of **1–3**

Table S18: Cartesian coordinates for the optimized geometry of dinuclear **1**.

Center Number	Element	Coordinates (Å) X	Y	Z
1	Cu	0.000000	-1.236880	0.000018
2	Cu	0.000000	1.236880	-0.000018
3	N	-2.537476	0.000000	0.000095
4	N	-1.898086	-1.099244	-0.000610
5	N	-1.898086	1.099244	0.000751
6	C	-2.803230	-2.270349	-0.001334
7	C	-2.803230	2.270349	0.001545
8	N	2.537476	0.000000	-0.000095
9	N	1.898086	1.099244	-0.000784
10	N	1.898086	-1.099244	0.000642
11	C	2.803230	2.270349	-0.001611
12	C	2.803230	-2.270349	0.001401
13	C	1.927574	3.510415	-0.003221
14	H	1.291039	3.536613	-0.890532
15	H	1.290376	3.538494	0.883556
16	H	2.543721	4.410529	-0.003930
17	C	3.672791	2.259975	-1.251598
18	H	4.307593	3.147476	-1.282065
19	H	4.311412	1.377456	-1.265195
20	H	3.051981	2.248658	-2.149232
21	C	3.671743	2.262401	1.249123
22	H	4.306647	3.149872	1.278362
23	H	3.050183	2.252950	2.146260
24	H	4.310227	1.379815	1.265006

25	C	-3.671744	2.262364	-1.249188
26	H	-4.306648	3.149834	-1.278453
27	H	-3.050185	2.252886	-2.146326
28	H	-4.310228	1.379778	-1.265045
29	C	-3.672790	2.260012	1.251532
30	H	-4.307592	3.147515	1.281973
31	H	-4.311411	1.377494	1.265157
32	H	-3.051979	2.248723	2.149166
33	C	-3.672889	-2.260010	-1.251254
34	H	-4.307693	-3.147513	-1.281646
35	H	-4.311511	-1.377492	-1.264826
36	H	-3.052148	-2.248719	-2.148936
37	C	-3.671646	-2.262366	1.249467
38	H	-3.050016	-2.252889	2.146556
39	H	-4.310128	-1.379779	1.265375
40	H	-4.306548	-3.149836	1.278781
41	C	-1.927574	-3.510415	-0.002977
42	H	-1.291109	-3.536638	-0.890336
43	H	-1.290307	-3.538469	0.883751
44	H	-2.543722	-4.410529	-0.003613
45	C	1.927574	-3.510415	0.003081
46	H	1.291109	-3.536611	0.890442
47	H	1.290307	-3.538496	-0.883645
48	H	2.543721	-4.410529	0.003744
49	C	3.672890	-2.259973	1.251319
50	H	4.307694	-3.147475	1.281737
51	H	4.311512	-1.377455	1.264865
52	H	3.052150	-2.248655	2.149002
53	C	3.671645	-2.262404	-1.249402
54	H	3.050014	-2.252954	-2.146490
55	H	4.310127	-1.379817	-1.265337
56	H	4.306547	-3.149874	-1.278689
57	C	-1.927574	3.510415	0.003116
58	H	-1.291039	3.536640	0.890426
59	H	-1.290377	3.538467	-0.883662
60	H	-2.543721	4.410529	0.003799

Table S19: Cartesian coordinates for the geometry of dinuclear **1** optimized using the SMD continuum solvation model.

Center		Coordinates (Å)		
Number	Element	X	Y	Z
1	Cu	-0.000001	1.230591	0.000026
2	Cu	0.000001	-1.230602	-0.000026

3	N	2.543851	0.000000	0.000079
4	N	1.900765	1.090949	-0.125015
5	N	1.900761	-1.090951	0.125126
6	C	2.798093	2.258846	-0.276521
7	C	2.798084	-2.258842	0.276700
8	N	-2.543851	-0.000004	-0.000079
9	N	-1.900759	-1.090950	-0.125161
10	N	-1.900767	1.090942	0.125050
11	C	-2.798081	-2.258837	-0.276782
12	C	-2.798097	2.258832	0.276602
13	C	-1.914425	-3.476721	-0.481655
14	H	-1.291861	-3.360586	-1.372117
15	H	-1.263390	-3.637030	0.381100
16	H	-2.524499	-4.372181	-0.611261
17	C	-3.699416	-2.080041	-1.490468
18	H	-4.315726	-2.968613	-1.642072
19	H	-4.360154	-1.223193	-1.360776
20	H	-3.102855	-1.918684	-2.390802
21	C	-3.633022	-2.441570	0.982992
22	H	-4.258447	-3.333310	0.904230
23	H	-2.988622	-2.550795	1.857933
24	H	-4.282006	-1.580390	1.143191
25	C	3.632980	-2.441558	-0.983106
26	H	4.258409	-3.333298	-0.904379
27	H	2.988548	-2.550771	-1.858025
28	H	4.281957	-1.580374	-1.143317
29	C	3.699463	-2.080069	1.490358
30	H	4.315771	-2.968648	1.641927
31	H	4.360205	-1.223225	1.360654
32	H	3.102935	-1.918720	2.390714
33	C	3.699611	2.080055	-1.490072
34	H	4.315921	2.968641	-1.641594
35	H	4.360353	1.223226	-1.360273
36	H	3.103187	1.918674	-2.390492
37	C	3.632845	2.441599	0.983375
38	H	2.988313	2.550828	1.858219
39	H	4.281810	1.580424	1.143680
40	H	4.258276	3.333342	0.904699
41	C	1.914450	3.476716	-0.481536
42	H	1.292016	3.360561	-1.372087
43	H	1.263287	3.637024	0.381122
44	H	2.524530	4.372183	-0.611062
45	C	-1.914454	3.476704	0.481603

46	H	-1.291989	3.360542	1.372131
47	H	-1.263322	3.637029	-0.381076
48	H	-2.524535	4.372165	0.611164
49	C	-3.699570	2.080016	1.490183
50	H	-4.315885	2.968592	1.641738
51	H	-4.360306	1.223181	1.360396
52	H	-3.103112	1.918629	2.390580
53	C	-3.632895	2.441600	-0.983261
54	H	-2.988395	2.550842	-1.858127
55	H	-4.281865	1.580426	-1.143554
56	H	-4.258324	3.333340	-0.904550
57	C	1.914432	-3.476727	0.481587
58	H	1.291899	-3.360602	1.372072
59	H	1.263366	-3.637021	-0.381147
60	H	2.524507	-4.372190	0.611159

Table S20: Cartesian coordinates for the optimized geometry of dinuclear **2**.

Center		Coordinates (Å)		
Number	Element	X	Y	Z
1	Ag	-0.000011	1.351716	0.000072
2	Ag	-0.000024	-1.351712	0.000111
3	N	-2.729726	0.000016	-0.000078
4	N	-2.105657	1.104951	0.000125
5	N	-2.105667	-1.104925	-0.000204
6	C	-3.025264	2.265148	0.000187
7	C	-3.025285	-2.265115	-0.000399
8	N	2.729692	-0.000012	0.000123
9	N	2.105622	-1.104946	0.000312
10	N	2.105632	1.104929	-0.000082
11	C	3.025230	-2.265144	0.000478
12	C	3.025251	2.265119	-0.000242
13	C	2.166933	-3.518189	0.000942
14	H	1.531700	-3.556318	0.889563
15	H	1.531444	-3.556813	-0.887477
16	H	2.795347	-4.409761	0.001093
17	C	3.894519	-2.246091	1.250729
18	H	4.539744	-3.126086	1.281851
19	H	4.522598	-1.356064	1.264303
20	H	3.273356	-2.241357	2.148230
21	C	3.894114	-2.246717	-1.250064
22	H	4.539363	-3.126702	-1.280947
23	H	3.272662	-2.242463	-2.147368
24	H	4.522154	-1.356670	-1.264296

25	C	-3.894209	-2.246679	1.250116
26	H	-4.539466	-3.126660	1.280979
27	H	-3.272785	-2.242430	2.147439
28	H	-4.522243	-1.356628	1.264326
29	C	-3.894535	-2.246055	-1.250678
30	H	-4.539767	-3.126044	-1.281819
31	H	-4.522605	-1.356022	-1.264273
32	H	-3.273344	-2.241328	-2.148159
33	C	-3.894659	2.246125	1.250365
34	H	-4.539886	3.126121	1.281412
35	H	-4.522741	1.356099	1.263907
36	H	-3.273573	2.241411	2.147919
37	C	-3.894042	2.246692	-1.250428
38	H	-3.272514	2.242419	-2.147679
39	H	-4.522080	1.356645	-1.264693
40	H	-4.539290	3.126676	-1.281386
41	C	-2.166968	3.518194	0.000696
42	H	-1.531809	3.556342	0.889369
43	H	-1.531405	3.556798	-0.887671
44	H	-2.795381	4.409765	0.000775
45	C	2.166965	3.518172	-0.000768
46	H	1.531762	3.556282	-0.889411
47	H	1.531447	3.556825	0.887629
48	H	2.795386	4.409738	-0.000922
49	C	3.894586	2.246028	-1.250460
50	H	4.539821	3.126016	-1.281580
51	H	4.522657	1.355995	-1.263991
52	H	3.273456	2.241279	-2.147984
53	C	3.894089	2.246715	1.250333
54	H	3.272604	2.242487	2.147614
55	H	4.522122	1.356664	1.264609
56	H	4.539344	3.126696	1.281219
57	C	-2.167000	-3.518167	-0.000836
58	H	-1.531735	-3.556299	-0.889434
59	H	-1.531543	-3.556799	0.887606
60	H	-2.795421	-4.409733	-0.001011

Table S21: Cartesian coordinates for the geometry of dinuclear **2** optimized using the SMD continuum solvation model.

Center		Coordinates (Å)		
Number	Element	X	Y	Z
1	Ag	-0.000011	1.354618	0.000256
2	Ag	-0.000024	-1.354615	-0.000222

3	N	-2.738299	0.000016	-0.000036
4	N	-2.113080	1.104805	0.000253
5	N	-2.113091	-1.104780	-0.000289
6	C	-3.031092	2.267394	0.000203
7	C	-3.031114	-2.267360	-0.000292
8	N	2.738264	-0.000011	0.000047
9	N	2.113045	-1.104801	0.000062
10	N	2.113056	1.104784	0.000020
11	C	3.031058	-2.267390	-0.000537
12	C	3.031080	2.267364	0.000638
13	C	2.170920	-3.518482	0.001929
14	H	1.537040	-3.556739	0.891773
15	H	1.534024	-3.558218	-0.885709
16	H	2.797573	-4.411853	0.001562
17	C	3.901698	-2.251347	1.248261
18	H	4.536410	-3.139249	1.284892
19	H	4.543618	-1.370646	1.260245
20	H	3.282373	-2.235005	2.147615
21	C	3.897204	-2.253037	-1.252478
22	H	4.532289	-3.140639	-1.289997
23	H	3.274646	-2.238452	-2.149631
24	H	4.538564	-1.371966	-1.268169
25	C	-3.898545	-2.252792	1.250758
26	H	-4.533520	-3.140490	1.287836
27	H	-3.276909	-2.237893	2.148543
28	H	-4.540070	-1.371832	1.265578
29	C	-3.900472	-2.251516	-1.249986
30	H	-4.535311	-3.139311	-1.287056
31	H	-4.542214	-1.370692	-1.262841
32	H	-3.280224	-2.235500	-2.148710
33	C	-3.900556	2.251537	1.249823
34	H	-4.535384	3.139341	1.286857
35	H	-4.542313	1.370723	1.262607
36	H	-3.280384	2.235493	2.148599
37	C	-3.898417	2.252857	-1.250920
38	H	-3.276705	2.237973	-2.148654
39	H	-4.539944	1.371899	-1.265812
40	H	-4.533385	3.140558	-1.288035
41	C	-2.170952	3.518487	0.001634
42	H	-1.536312	3.556965	0.890931
43	H	-1.534814	3.558002	-0.886553
44	H	-2.797605	4.411858	0.001607
45	C	2.170954	3.518465	-0.001867

46	H	1.537104	3.556720	-0.891733
47	H	1.534028	3.558215	0.885748
48	H	2.797616	4.411830	-0.001486
49	C	3.901761	2.251303	-1.248132
50	H	4.536480	3.139201	-1.284750
51	H	4.543676	1.370598	-1.260086
52	H	3.282465	2.234957	-2.147506
53	C	3.897184	2.253013	1.252608
54	H	3.274597	2.238443	2.149740
55	H	4.538533	1.371933	1.268327
56	H	4.532279	3.140607	1.290139
57	C	-2.170986	-3.518460	-0.001625
58	H	-1.536275	-3.556966	-0.890870
59	H	-1.534920	-3.557960	0.886614
60	H	-2.797647	-4.411826	-0.001629

Table S22: Cartesian coordinates for the optimized geometry of dinuclear **3**.

Center		Coordinates (Å)		
Number	Element	X	Y	Z
1	Au	0.000002	-1.349046	-0.000021
2	Au	-0.000002	1.349045	0.000017
3	N	2.647173	0.000002	-0.000101
4	N	2.045083	-1.117867	0.045891
5	N	2.045077	1.117870	-0.046051
6	C	2.995858	-2.259763	0.033438
7	C	2.995850	2.259769	-0.033664
8	N	-2.647173	-0.000002	0.000102
9	N	-2.045080	1.117866	0.046081
10	N	-2.045080	-1.117869	-0.045924
11	C	-2.995855	2.259762	0.033731
12	C	-2.995853	-2.259768	-0.033499
13	C	-2.212453	3.524840	0.335377
14	H	-1.727860	3.460953	1.311268
15	H	-1.444755	3.703539	-0.420355
16	H	-2.886044	4.382936	0.342883
17	C	-4.069662	2.072593	1.096799
18	H	-4.722289	2.946752	1.122385
19	H	-4.678091	1.194204	0.888917
20	H	-3.615663	1.953055	2.081934
21	C	-3.626377	2.362832	-1.348426
22	H	-4.301369	3.219059	-1.399344
23	H	-2.855656	2.485004	-2.111509

24	H	-4.194250	1.460164	-1.575095
25	C	3.626357	2.362805	1.348502
26	H	4.301347	3.219033	1.399449
27	H	2.855629	2.484955	2.111582
28	H	4.194232	1.460134	1.575153
29	C	4.069666	2.072631	-1.096726
30	H	4.722291	2.946792	-1.122284
31	H	4.678096	1.194238	-0.888861
32	H	3.615677	1.953117	-2.081869
33	C	4.069739	-2.072627	1.096437
34	H	4.722370	-2.946785	1.121949
35	H	4.678152	-1.194230	0.888541
36	H	3.615808	-1.953122	2.081608
37	C	3.626283	-2.362789	-1.348766
38	H	2.855509	-2.484937	-2.111800
39	H	4.194141	-1.460114	-1.575446
40	H	4.301272	-3.219014	-1.399758
41	C	2.212478	-3.524850	0.335098
42	H	1.727953	-3.460996	1.311025
43	H	1.444726	-3.703526	-0.420585
44	H	2.886069	-4.382947	0.342530
45	C	-2.212470	-3.524844	-0.335203
46	H	-1.727952	-3.460958	-1.311131
47	H	-1.444713	-3.703540	0.420470
48	H	-2.886059	-4.382942	-0.342656
49	C	-4.069741	-2.072604	-1.096485
50	H	-4.722369	-2.946763	-1.122018
51	H	-4.678156	-1.194214	-0.888557
52	H	-3.615818	-1.953067	-2.081655
53	C	-3.626267	-2.362837	1.348707
54	H	-2.855486	-2.485006	2.111731
55	H	-4.194126	-1.460171	1.575418
56	H	-4.301253	-3.219066	1.399678
57	C	2.212446	3.524852	-0.335284
58	H	1.727861	3.460988	-1.311181
59	H	1.444741	3.703530	0.420446
60	H	2.886034	4.382950	-0.342764

Table S23: Cartesian coordinates for the geometry of dinuclear **3** optimized using the SMD continuum solvation model.

Center		Coordinates (Å)		
Number	Element	X	Y	Z
1	Au	0.014301	1.348028	-0.071754

2	Au	-0.014324	-1.348034	-0.070943
3	N	-2.654258	0.036047	-0.047211
4	N	-2.035886	1.149857	-0.091192
5	N	-2.059283	-1.082277	-0.047055
6	C	-2.964511	2.306985	-0.026501
7	C	-2.948514	-2.265983	0.003095
8	N	2.654238	-0.036038	-0.047938
9	N	2.035861	-1.149870	-0.091180
10	N	2.059266	1.082290	-0.048402
11	C	2.964447	-2.306990	-0.025879
12	C	2.948517	2.266005	0.001216
13	C	2.186590	-3.551671	-0.413918
14	H	1.360509	-3.734029	0.277398
15	H	1.778401	-3.460372	-1.422480
16	H	2.843504	-4.422736	-0.388832
17	C	3.477369	-2.445845	1.400706
18	H	4.131151	-3.315427	1.494205
19	H	4.042099	-1.559026	1.692196
20	H	2.644862	-2.565430	2.097296
21	C	4.125033	-2.126503	-0.993965
22	H	4.745207	-3.024827	-0.998962
23	H	3.756584	-1.959595	-2.008140
24	H	4.750559	-1.281000	-0.712821
25	C	-2.662980	-2.999878	1.306321
26	H	-3.255013	-3.914833	1.368270
27	H	-1.607154	-3.271649	1.373851
28	H	-2.906804	-2.370393	2.164148
29	C	-4.420183	-1.899325	-0.065847
30	H	-5.009425	-2.817883	-0.043574
31	H	-4.723250	-1.278817	0.776855
32	H	-4.656478	-1.363701	-0.985331
33	C	-3.478066	2.446141	1.399826
34	H	-4.131900	3.315737	1.492839
35	H	-4.042918	1.559386	1.691261
36	H	-2.645868	2.565895	2.096756
37	C	-4.124658	2.126247	-0.995067
38	H	-3.755744	1.959232	-2.009056
39	H	-4.750195	1.280705	-0.714056
40	H	-4.744946	3.024490	-1.000470
41	C	-2.186511	3.551602	-0.414450
42	H	-1.360693	3.734079	0.277151
43	H	-1.777946	3.460145	-1.422846
44	H	-2.843443	4.422666	-0.389763

45	C	2.591636	3.154991	-1.181917
46	H	2.773337	2.634806	-2.124206
47	H	1.537740	3.441545	-1.149594
48	H	3.191845	4.066468	-1.167869
49	C	4.420140	1.899375	-0.068854
50	H	5.009373	2.817956	-0.047291
51	H	4.723945	1.279075	0.773731
52	H	4.655666	1.363543	-0.988419
53	C	2.663902	2.999788	1.304710
54	H	1.608111	3.271502	1.373022
55	H	2.908365	2.370228	2.162302
56	H	3.255947	3.914760	1.366325
57	C	-2.592485	-3.154886	-1.180361
58	H	-2.774891	-2.634638	-2.122479
59	H	-1.538554	-3.441398	-1.148823
60	H	-3.192657	-4.066382	-1.165941

Table S24: Cartesian coordinates for the optimized geometry of tetranuclear **1** in the rhombic conformation.

Center		Coordinates (Å)		
Number	Element	X	Y	Z
1	Cu	0.007636	0.014517	-1.448116
2	Cu	-0.026137	0.030366	1.445146
3	Cu	2.346687	-0.028367	0.029227
4	Cu	-2.363056	-0.034014	-0.022622
5	N	1.780701	2.289807	-1.538963
6	N	2.590511	1.681926	-0.776233
7	N	0.651378	1.777716	-1.806753
8	C	3.896673	2.371245	-0.633207
9	C	-0.116998	2.647988	-2.734275
10	N	-1.783460	2.315890	1.499520
11	N	-0.668399	1.796640	1.796107
12	N	-2.600490	1.690417	0.752292
13	C	0.130675	2.689251	2.674669
14	C	-3.884032	2.429995	0.630619
15	N	-1.692046	-2.321204	-1.590912
16	N	-2.521337	-1.762673	-0.811261
17	N	-0.596809	-1.747455	-1.871813
18	N	2.517517	-1.741981	0.846427
19	N	1.686136	-2.295407	1.627492
20	N	0.588780	-1.719716	1.897431
21	C	-0.235970	-2.543558	2.817980

22	C	-3.799039	-2.511361	-0.711783
23	C	0.225639	-2.587887	-2.779258
24	C	3.798016	-2.482578	0.732009
25	C	-4.377139	-2.274073	0.674807
26	H	-3.747885	-2.730825	1.439024
27	H	-4.461369	-1.208313	0.899109
28	H	-5.374567	-2.710040	0.747025
29	C	-3.616505	-4.009524	-0.912224
30	H	-4.567939	-4.510421	-0.723873
31	H	-3.298049	-4.245126	-1.925350
32	H	-2.875551	-4.411942	-0.221228
33	C	-4.747402	-1.975190	-1.778565
34	H	-5.717664	-2.472247	-1.719667
35	H	-4.903132	-0.902524	-1.661495
36	H	-4.329384	-2.150667	-2.770977
37	C	-4.589254	2.432429	1.981436
38	H	-5.563769	2.918719	1.906012
39	H	-4.740511	1.411003	2.336036
40	H	-3.992065	2.965684	2.721008
41	C	-4.751018	1.710047	-0.386264
42	H	-4.248945	1.632553	-1.352530
43	H	-5.007666	0.706606	-0.044606
44	H	-5.683713	2.256353	-0.533705
45	C	-3.652115	3.859419	0.160068
46	H	-4.605755	4.385020	0.084348
47	H	-3.015322	4.399454	0.858791
48	H	-3.178004	3.871298	-0.822015
49	C	-0.692368	3.144463	3.872803
50	H	-0.069715	3.732663	4.549454
51	H	-1.534973	3.757665	3.557983
52	H	-1.078608	2.284174	4.422663
53	C	0.603431	3.892701	1.872196
54	H	1.165266	3.567828	0.995900
55	H	-0.251466	4.478142	1.532774
56	H	1.241169	4.536185	2.481876
57	C	1.324672	1.893524	3.172378
58	H	1.005465	1.044167	3.780202
59	H	1.919767	1.514746	2.338477
60	H	1.969029	2.525592	3.785458
61	C	4.937446	1.574967	-1.411744
62	H	5.916308	2.053163	-1.344422
63	H	5.030736	0.563261	-1.014172
64	H	4.656663	1.505547	-2.463639

65	C	4.268505	2.389680	0.842513
66	H	4.224983	1.387398	1.273898
67	H	5.284270	2.766752	0.972046
68	H	3.589820	3.030072	1.405674
69	C	3.868759	3.801271	-1.149624
70	H	4.834999	4.265536	-0.943806
71	H	3.687104	3.841975	-2.222110
72	H	3.093176	4.385429	-0.654291
73	C	0.561684	-2.922788	4.058934
74	H	-0.073309	-3.468996	4.758979
75	H	1.410232	-3.553042	3.798250
76	H	0.936850	-2.029275	4.561576
77	C	-1.433917	-1.710042	3.239150
78	H	-1.122073	-0.821753	3.792825
79	H	-2.014285	-1.386491	2.371647
80	H	-2.090509	-2.296424	3.883924
81	C	-0.705499	-3.792470	2.086942
82	H	-1.244622	-3.513415	1.181213
83	H	0.149184	-4.406361	1.801830
84	H	-1.364375	-4.388720	2.721639
85	C	1.439007	-1.773356	-3.191075
86	H	1.143394	-0.884980	-3.753390
87	H	2.011874	-1.452126	-2.318514
88	H	2.096595	-2.371213	-3.824101
89	C	-0.565835	-2.959000	-4.026785
90	H	0.064190	-3.525012	-4.715518
91	H	-1.431670	-3.567564	-3.770765
92	H	-0.914662	-2.061139	-4.540563
93	C	0.664499	-3.843386	-2.038889
94	H	1.188422	-3.575494	-1.120555
95	H	-0.204185	-4.445809	-1.772491
96	H	1.327722	-4.447767	-2.661272
97	C	4.220605	-2.488246	-0.729641
98	H	3.541034	-3.093069	-1.329396
99	H	4.229527	-1.478096	-1.143716
100	H	5.226170	-2.899868	-0.829783
101	C	4.842627	-1.745580	1.562647
102	H	5.804899	-2.258646	1.514645
103	H	4.985066	-0.728188	1.194575
104	H	4.529930	-1.691433	2.606411
105	C	3.692050	-3.920380	1.216408
106	H	4.637983	-4.427409	1.017260
107	H	3.489260	-3.975070	2.284216

108	H	2.898057	-4.454721	0.694422
109	C	-1.476777	2.008352	-2.948866
110	H	-1.385442	1.023272	-3.413673
111	H	-1.998074	1.894082	-1.996686
112	H	-2.086925	2.632819	-3.603470
113	C	0.611650	2.747713	-4.068237
114	H	1.589738	3.209442	-3.935293
115	H	0.754119	1.756768	-4.503654
116	H	0.036365	3.351805	-4.772459
117	C	-0.296124	4.032118	-2.126998
118	H	-0.918804	4.652911	-2.774235
119	H	-0.774315	3.958200	-1.149885
120	H	0.668105	4.523168	-2.001326

Table S25: Cartesian coordinates for the geometry of tetranuclear **1** in the rhombic conformation optimized using the SMD continuum solvation model.

Center		Coordinates (Å)		
Number	Element	X	Y	Z
1	Cu	0.007942	0.020920	1.435066
2	Cu	0.017215	0.028904	-1.443221
3	Cu	-2.319445	-0.032727	-0.011133
4	Cu	2.344065	-0.033475	0.004376
5	N	-1.749978	2.297841	1.571334
6	N	-2.568179	1.679838	0.824474
7	N	-0.609060	1.794925	1.812644
8	C	-3.884574	2.353752	0.706296
9	C	0.181257	2.663711	2.722089
10	N	1.759431	2.322497	-1.537809
11	N	0.637109	1.804916	-1.814163
12	N	2.584335	1.693128	-0.800985
13	C	-0.182899	2.692454	-2.676641
14	C	3.872621	2.427457	-0.696162
15	N	1.673311	-2.328924	1.590908
16	N	2.519335	-1.754879	0.840391
17	N	0.564244	-1.763967	1.837675
18	N	-2.519397	-1.735744	-0.877224
19	N	-1.672238	-2.308994	-1.626798
20	N	-0.557429	-1.748494	-1.860669
21	C	0.293411	-2.582660	-2.746749
22	C	3.810307	-2.484193	0.777075
23	C	-0.287809	-2.607684	2.712890
24	C	-3.816310	-2.451762	-0.798757
25	C	4.398199	-2.274645	-0.608593

26	H	3.804318	-2.791298	-1.363604
27	H	4.425718	-1.214845	-0.870416
28	H	5.418600	-2.659527	-0.652989
29	C	3.658355	-3.979305	1.016870
30	H	4.621101	-4.464533	0.841988
31	H	3.345628	-4.200388	2.035678
32	H	2.928480	-4.416760	0.334603
33	C	4.734770	-1.899144	1.838650
34	H	5.712475	-2.385458	1.814296
35	H	4.881551	-0.829139	1.686633
36	H	4.304128	-2.043515	2.831478
37	C	4.560500	2.432438	-2.055550
38	H	5.543198	2.904500	-1.989184
39	H	4.694593	1.412518	-2.422601
40	H	3.964549	2.980220	-2.786267
41	C	4.749742	1.699962	0.305838
42	H	4.258379	1.618912	1.277541
43	H	4.999962	0.697903	-0.044094
44	H	5.686105	2.242969	0.446671
45	C	3.656571	3.856119	-0.216355
46	H	4.615154	4.374630	-0.146806
47	H	3.018516	4.409413	-0.904103
48	H	3.192881	3.868427	0.771148
49	C	0.603645	3.137406	-3.901952
50	H	-0.042785	3.705514	-4.574373
51	H	1.445747	3.769355	-3.622630
52	H	0.988905	2.273461	-4.447687
53	C	-0.634599	3.902001	-1.871346
54	H	-1.169746	3.581903	-0.976163
55	H	0.225949	4.496211	-1.561680
56	H	-1.293210	4.538936	-2.466259
57	C	-1.391758	1.891395	-3.126535
58	H	-1.096152	1.052074	-3.760063
59	H	-1.932546	1.494577	-2.263888
60	H	-2.076261	2.521464	-3.697228
61	C	-4.906595	1.527373	1.477245
62	H	-5.891148	1.998136	1.439112
63	H	-4.999553	0.525613	1.054799
64	H	-4.610284	1.430484	2.523431
65	C	-4.263859	2.393324	-0.766017
66	H	-4.180927	1.402333	-1.216886
67	H	-5.293615	2.735347	-0.886990
68	H	-3.610166	3.069780	-1.317432

69	C	-3.879303	3.773460	1.249715
70	H	-4.855754	4.223577	1.058275
71	H	-3.694979	3.800045	2.322849
72	H	-3.119926	4.384559	0.761217
73	C	-0.459454	-2.965634	-4.013964
74	H	0.201916	-3.506137	-4.694620
75	H	-1.312541	-3.603735	-3.786768
76	H	-0.824672	-2.074545	-4.529229
77	C	1.510138	-1.757032	-3.128765
78	H	1.225413	-0.881327	-3.716533
79	H	2.045423	-1.410755	-2.240063
80	H	2.199120	-2.355006	-3.728222
81	C	0.732083	-3.830253	-1.994655
82	H	1.231532	-3.551431	-1.065774
83	H	-0.131378	-4.448379	-1.745600
84	H	1.418646	-4.427650	-2.599046
85	C	-1.506902	-1.787174	3.096009
86	H	-1.223607	-0.920350	3.697381
87	H	-2.032467	-1.426916	2.207430
88	H	-2.203135	-2.391352	3.680502
89	C	0.462116	-2.996461	3.980150
90	H	-0.197551	-3.548604	4.653066
91	H	1.322044	-3.624876	3.751347
92	H	0.817121	-2.106550	4.504662
93	C	-0.715883	-3.851785	1.948102
94	H	-1.211221	-3.568920	1.017668
95	H	0.153127	-4.462413	1.699865
96	H	-1.401948	-4.458040	2.544030
97	C	-4.244133	-2.503931	0.659212
98	H	-3.582145	-3.150460	1.236014
99	H	-4.221316	-1.509944	1.110099
100	H	-5.262015	-2.889345	0.744494
101	C	-4.838785	-1.658445	-1.603699
102	H	-5.809416	-2.158757	-1.594569
103	H	-4.974055	-0.658665	-1.187645
104	H	-4.513056	-1.555504	-2.640526
105	C	-3.747049	-3.870690	-1.340354
106	H	-4.710178	-4.356659	-1.170381
107	H	-3.538428	-3.890390	-2.409038
108	H	-2.976415	-4.453318	-0.834787
109	C	1.553314	2.033240	2.885917
110	H	1.481961	1.035304	3.327313
111	H	2.049141	1.944765	1.917277

112	H	2.176783	2.646965	3.538829
113	C	-0.502166	2.746873	4.080529
114	H	-1.489249	3.200810	3.988834
115	H	-0.621248	1.751741	4.514595
116	H	0.090629	3.350762	4.771096
117	C	0.333907	4.053937	2.122363
118	H	0.981339	4.670669	2.749746
119	H	0.774431	3.990116	1.126403
120	H	-0.633312	4.549102	2.038417

Table S26: Cartesian coordinates for the optimized geometry of tetranuclear **2** in the rhombic conformation.

Center		Coordinates (Å)		
Number	Element	X	Y	Z
1	Ag	-0.000635	0.023017	1.617904
2	Ag	0.025639	-0.003667	-1.635691
3	Ag	-2.451311	-0.007268	-0.031508
4	Ag	2.470198	-0.023808	0.010963
5	N	-1.846600	2.489958	1.643654
6	N	-2.674284	1.861634	0.924011
7	N	-0.706348	2.003724	1.904036
8	C	-3.987332	2.532241	0.781841
9	C	0.077696	2.913689	2.777385
10	N	1.849175	2.485541	-1.639781
11	N	0.736418	1.973470	-1.949041
12	N	2.681886	1.863039	-0.913714
13	C	-0.078143	2.903176	-2.773401
14	C	3.954352	2.617410	-0.781554
15	N	1.790510	-2.492640	1.695563
16	N	2.624953	-1.911612	0.943731
17	N	0.684189	-1.951063	1.988266
18	N	-2.636190	-1.884188	-0.978467
19	N	-1.796100	-2.496743	-1.697664
20	N	-0.677814	-1.975240	-1.982584
21	C	0.156023	-2.896643	-2.795268
22	C	3.915244	-2.631952	0.830835
23	C	-0.140705	-2.845951	2.839459
24	C	-3.936468	-2.579594	-0.837763
25	C	4.431921	-2.461478	-0.589983
26	H	3.754838	-2.927342	-1.306600
27	H	4.538282	-1.406417	-0.855428
28	H	5.413422	-2.926649	-0.692057
29	C	3.783625	-4.118992	1.125502

30	H	4.743169	-4.604419	0.938470
31	H	3.500391	-4.302192	2.159797
32	H	3.033252	-4.580175	0.482503
33	C	4.892620	-1.999578	1.815417
34	H	5.876948	-2.465571	1.739195
35	H	5.003546	-0.931447	1.619390
36	H	4.529980	-2.121207	2.837149
37	C	4.658486	2.652315	-2.132336
38	H	5.623587	3.156251	-2.051521
39	H	4.828202	1.638954	-2.501216
40	H	4.049882	3.184303	-2.863492
41	C	4.832016	1.891216	0.222653
42	H	4.340354	1.813229	1.194939
43	H	5.082666	0.887005	-0.126481
44	H	5.767866	2.433956	0.362355
45	C	3.700419	4.035117	-0.286374
46	H	4.646588	4.568128	-0.176228
47	H	3.075291	4.583584	-0.989045
48	H	3.200524	4.022018	0.683145
49	C	0.712831	3.377030	-3.985883
50	H	0.085537	4.006142	-4.620105
51	H	1.583595	3.954507	-3.679711
52	H	1.054612	2.525421	-4.577082
53	C	-0.503867	4.090577	-1.920917
54	H	-1.045619	3.749275	-1.037415
55	H	0.370513	4.651624	-1.590630
56	H	-1.149139	4.762156	-2.490985
57	C	-1.308804	2.152463	-3.250125
58	H	-1.034594	1.302924	-3.879772
59	H	-1.897833	1.786609	-2.406081
60	H	-1.945855	2.814002	-3.839046
61	C	-5.023957	1.681108	1.506329
62	H	-6.018854	2.119168	1.408820
63	H	-5.058992	0.671959	1.090023
64	H	-4.780619	1.603311	2.566872
65	C	-4.330712	2.602972	-0.699973
66	H	-4.314752	1.611554	-1.159564
67	H	-5.330815	3.016782	-0.838684
68	H	-3.619068	3.235354	-1.231451
69	C	-3.998124	3.939314	1.357274
70	H	-4.980145	4.382647	1.182857
71	H	-3.804319	3.938119	2.428643
72	H	-3.245728	4.567079	0.880198

73	C	-0.618515	-3.400010	-4.006566
74	H	0.026707	-4.019580	-4.632198
75	H	-1.476563	-3.995266	-3.699380
76	H	-0.977061	-2.561861	-4.607064
77	C	1.373735	-2.127402	-3.276336
78	H	1.084799	-1.290194	-3.915800
79	H	1.953100	-1.739622	-2.435076
80	H	2.025401	-2.782704	-3.856254
81	C	0.600842	-4.065488	-1.927055
82	H	1.132338	-3.700429	-1.046851
83	H	-0.264943	-4.636975	-1.591556
84	H	1.260514	-4.733253	-2.485179
85	C	-1.383956	-2.082241	3.258950
86	H	-1.126164	-1.205421	3.857582
87	H	-1.956616	-1.754904	2.388480
88	H	-2.030167	-2.721218	3.862661
89	C	0.631670	-3.256634	4.086629
90	H	0.001389	-3.867311	4.735818
91	H	1.516193	-3.833631	3.821511
92	H	0.949828	-2.375448	4.646981
93	C	-0.543275	-4.075306	2.036043
94	H	-1.068120	-3.777706	1.126752
95	H	0.340710	-4.645009	1.749088
96	H	-1.197168	-4.723117	2.623573
97	C	-4.289912	-2.645556	0.641679
98	H	-3.573884	-3.263844	1.183459
99	H	-4.292564	-1.651063	1.094701
100	H	-5.284972	-3.073032	0.775028
101	C	-4.984396	-1.754166	-1.576006
102	H	-5.971068	-2.211518	-1.483326
103	H	-5.042731	-0.743142	-1.166788
104	H	-4.734184	-1.678618	-2.635099
105	C	-3.916785	-3.990621	-1.403196
106	H	-4.887302	-4.455610	-1.220934
107	H	-3.729412	-3.991551	-2.475637
108	H	-3.147165	-4.597414	-0.926511
109	C	1.470689	2.329496	2.930221
110	H	1.445541	1.359403	3.433667
111	H	1.941821	2.204410	1.953256
112	H	2.093278	2.996281	3.528834
113	C	-0.587683	3.006869	4.144407
114	H	-1.584491	3.438258	4.053048
115	H	-0.681180	2.015762	4.592932

116	H	0.001077	3.634065	4.816625
117	C	0.184533	4.294875	2.145466
118	H	0.804398	4.946495	2.764327
119	H	0.635939	4.225018	1.154927
120	H	-0.799873	4.749552	2.044086

Table S27: Cartesian coordinates for the geometry of tetranuclear **2** in the rhombic conformation optimized using the SMD continuum solvation model.

Center		Coordinates (Å)		
Number	Element	X	Y	Z
1	Ag	0.027548	0.086669	1.583804
2	Ag	0.031466	-0.063874	-1.611663
3	N	-1.852880	2.532680	1.626382
4	N	-2.683106	1.883498	0.928138
5	N	-0.698901	2.066135	1.866593
6	C	-4.008731	2.532947	0.796570
7	C	0.090200	2.985127	2.725554
8	N	1.861940	2.431307	-1.722683
9	N	0.746478	1.906293	-2.003848
10	N	2.699535	1.832733	-0.982046
11	C	-0.082432	2.805327	-2.848319
12	C	3.976414	2.584330	-0.877905
13	Ag	2.500588	-0.034440	-0.007489
14	N	1.805937	-2.447802	1.751281
15	N	2.650381	-1.893650	0.988997
16	N	0.697631	-1.891785	2.010556
17	Ag	-2.464740	-0.002651	-0.014739
18	N	-2.670676	-1.890453	-0.955166
19	N	-1.835815	-2.529704	-1.656985
20	N	-0.699503	-2.036827	-1.926837
21	C	0.118839	-2.975317	-2.736238
22	C	3.946093	-2.611934	0.929058
23	C	-0.144779	-2.750817	2.882452
24	C	-3.988563	-2.554653	-0.819159
25	C	4.530239	-2.423252	-0.462097
26	H	3.897150	-2.889610	-1.218243
27	H	4.639952	-1.364799	-0.713234
28	H	5.520753	-2.877808	-0.519830
29	C	3.804075	-4.102612	1.197785
30	H	4.770549	-4.586672	1.043231
31	H	3.481359	-4.303822	2.217524
32	H	3.082802	-4.557275	0.517070

33	C	4.873115	-1.989969	1.966779
34	H	5.860330	-2.456235	1.938667
35	H	4.996169	-0.919805	1.786273
36	H	4.459692	-2.118366	2.968933
37	C	4.703680	2.517179	-2.214567
38	H	5.674023	3.014766	-2.154018
39	H	4.868616	1.478641	-2.510154
40	H	4.116214	3.002872	-2.994703
41	C	4.826905	1.921047	0.191312
42	H	4.318646	1.921118	1.158261
43	H	5.068397	0.890139	-0.078037
44	H	5.769407	2.458633	0.307755
45	C	3.732927	4.034887	-0.484463
46	H	4.685884	4.555741	-0.370912
47	H	3.147367	4.555357	-1.240703
48	H	3.198040	4.093898	0.465128
49	C	0.688274	3.239886	-4.087755
50	H	0.045830	3.834246	-4.740802
51	H	1.555133	3.842819	-3.820352
52	H	1.035396	2.369912	-4.649419
53	C	-0.501671	4.018447	-2.030294
54	H	-1.019390	3.703411	-1.122578
55	H	0.371359	4.603851	-1.739841
56	H	-1.168925	4.662426	-2.607574
57	C	-1.317174	2.035686	-3.282211
58	H	-1.050294	1.166431	-3.888399
59	H	-1.893673	1.694010	-2.418812
60	H	-1.966027	2.675277	-3.883108
61	C	-5.020308	1.673812	1.545120
62	H	-6.024972	2.092135	1.457005
63	H	-5.043222	0.658509	1.142347
64	H	-4.762445	1.612312	2.604021
65	C	-4.371304	2.578499	-0.681279
66	H	-4.347598	1.580880	-1.127463
67	H	-5.379192	2.976311	-0.814157
68	H	-3.676472	3.213921	-1.232307
69	C	-4.038416	3.946067	1.354916
70	H	-5.031353	4.368642	1.188335
71	H	-3.834177	3.964117	2.424722
72	H	-3.307091	4.585947	0.860722
73	C	-0.629553	-3.387044	-3.997038
74	H	0.005315	-4.014920	-4.625813
75	H	-1.530388	-3.948277	-3.751420

76	H	-0.919993	-2.507275	-4.575547
77	C	1.396605	-2.258190	-3.132931
78	H	1.186165	-1.388404	-3.760487
79	H	1.948648	-1.925260	-2.250427
80	H	2.044432	-2.929022	-3.700043
81	C	0.465396	-4.198619	-1.899665
82	H	0.975714	-3.896720	-0.983312
83	H	-0.438038	-4.743950	-1.624716
84	H	1.118131	-4.875293	-2.455792
85	C	-1.390789	-1.966915	3.253404
86	H	-1.140937	-1.075595	3.834306
87	H	-1.941771	-1.657221	2.361768
88	H	-2.056218	-2.584033	3.859706
89	C	0.605327	-3.116608	4.156415
90	H	-0.041047	-3.690150	4.824130
91	H	1.486596	-3.717104	3.933610
92	H	0.928166	-2.216240	4.683764
93	C	-0.543808	-4.006982	2.120865
94	H	-1.045887	-3.741077	1.188882
95	H	0.336408	-4.602546	1.876569
96	H	-1.219407	-4.623296	2.718314
97	C	-4.349284	-2.604446	0.658744
98	H	-3.649914	-3.235260	1.209068
99	H	-4.332811	-1.607022	1.105611
100	H	-5.354274	-3.009517	0.791581
101	C	-5.010480	-1.705573	-1.565510
102	H	-6.010025	-2.136195	-1.478209
103	H	-5.045471	-0.691642	-1.160143
104	H	-4.753606	-1.638547	-2.624305
105	C	-4.005249	-3.966970	-1.379915
106	H	-4.991197	-4.402422	-1.205251
107	H	-3.811459	-3.979176	-2.451731
108	H	-3.261106	-4.599119	-0.895093
109	C	1.500760	2.432062	2.821090
110	H	1.517862	1.458549	3.319357
111	H	1.934936	2.321617	1.825485
112	H	2.133375	3.108459	3.398798
113	C	-0.529301	3.041737	4.115521
114	H	-1.537549	3.455121	4.070611
115	H	-0.588683	2.041503	4.550619
116	H	0.069617	3.668126	4.780261
117	C	0.145143	4.377731	2.113382
118	H	0.776301	5.031862	2.718700

119	H	0.560575	4.334581	1.105202
120	H	-0.849213	4.819210	2.056262

Table S28: Cartesian coordinates for the optimized geometry of tetranuclear **3** in the rhombic conformation.

Center		Coordinates (Å)		
Number	Element	X	Y	Z
1	Au	-0.001597	0.026348	1.717902
2	Au	0.020227	-0.006868	-1.734226
3	Au	-2.448140	-0.012450	-0.029865
4	Au	2.461940	-0.013340	0.008572
5	N	-1.796929	2.435676	1.608633
6	N	-2.637324	1.807498	0.904501
7	N	-0.654715	1.966269	1.890926
8	C	-3.950336	2.496281	0.782231
9	C	0.096030	2.899144	2.780023
10	N	1.777520	2.434505	-1.609806
11	N	0.666935	1.931351	-1.942608
12	N	2.627769	1.823249	-0.896155
13	C	-0.128893	2.900892	-2.752297
14	C	3.897420	2.602186	-0.804278
15	N	1.747000	-2.429715	1.660671
16	N	2.593769	-1.845814	0.925988
17	N	0.637331	-1.907992	1.977473
18	N	-2.594246	-1.840201	-0.956982
19	N	-1.737591	-2.452170	-1.656339
20	N	-0.619497	-1.943912	-1.965394
21	C	0.194413	-2.906357	-2.763824
22	C	3.887747	-2.575841	0.835351
23	C	-0.156990	-2.846069	2.823738
24	C	-3.891277	-2.558304	-0.832674
25	C	4.411375	-2.483726	-0.590005
26	H	3.734771	-2.981518	-1.284970
27	H	4.526850	-1.446006	-0.908556
28	H	5.386956	-2.967625	-0.655477
29	C	3.748923	-4.045264	1.204666
30	H	4.708487	-4.537777	1.038829
31	H	3.469984	-4.179264	2.247521
32	H	2.998615	-4.538257	0.586020
33	C	4.863845	-1.900683	1.791719
34	H	5.845420	-2.374806	1.735733
35	H	4.976511	-0.843816	1.546394

36	H	4.501193	-1.975192	2.818037
37	C	4.590091	2.554256	-2.160490
38	H	5.548905	3.074396	-2.119804
39	H	4.770763	1.520196	-2.459654
40	H	3.970684	3.028330	-2.922049
41	C	4.801317	1.970692	0.239600
42	H	4.318286	1.932188	1.217170
43	H	5.088438	0.957809	-0.046249
44	H	5.712818	2.563027	0.333166
45	C	3.620543	4.046399	-0.407021
46	H	4.563116	4.588951	-0.317692
47	H	3.004373	4.547614	-1.150841
48	H	3.108556	4.091999	0.555071
49	C	0.690655	3.384140	-3.942017
50	H	0.082759	4.038059	-4.569648
51	H	1.568247	3.938953	-3.614275
52	H	1.021113	2.538034	-4.547132
53	C	-0.523370	4.074906	-1.867425
54	H	-1.083150	3.724952	-0.999011
55	H	0.363457	4.601739	-1.515282
56	H	-1.144232	4.779542	-2.423984
57	C	-1.379707	2.212274	-3.267612
58	H	-1.131205	1.387798	-3.937902
59	H	-1.984136	1.819539	-2.449144
60	H	-1.983899	2.931154	-3.823699
61	C	-4.987191	1.646911	1.507751
62	H	-5.973128	2.109386	1.437305
63	H	-5.046670	0.649103	1.070041
64	H	-4.726723	1.542531	2.562003
65	C	-4.315694	2.606995	-0.691360
66	H	-4.321493	1.627623	-1.173145
67	H	-5.310911	3.041853	-0.796216
68	H	-3.605475	3.243586	-1.219484
69	C	-3.932351	3.890929	1.387150
70	H	-4.913264	4.344959	1.237056
71	H	-3.722707	3.867535	2.455323
72	H	-3.183847	4.522225	0.908979
73	C	-0.607228	-3.409778	-3.957387
74	H	0.018545	-4.052968	-4.578564
75	H	-1.475171	-3.981464	-3.634241
76	H	-0.949955	-2.572291	-4.567690
77	C	1.438824	-2.202776	-3.275134
78	H	1.182758	-1.387648	-3.954041

79	H	2.031054	-1.793073	-2.455804
80	H	2.057937	-2.916689	-3.821187
81	C	0.599498	-4.068366	-1.868178
82	H	1.150529	-3.702102	-1.000945
83	H	-0.282665	-4.602431	-1.514905
84	H	1.231728	-4.770294	-2.415422
85	C	-1.434196	-2.157473	3.269317
86	H	-1.220207	-1.296363	3.904657
87	H	-2.020923	-1.816712	2.415716
88	H	-2.041366	-2.859535	3.843389
89	C	0.645822	-3.237070	4.057924
90	H	0.043490	-3.873840	4.708254
91	H	1.547405	-3.781165	3.781341
92	H	0.935604	-2.348189	4.621201
93	C	-0.505771	-4.079926	2.002935
94	H	-1.052769	-3.793613	1.103388
95	H	0.399512	-4.606433	1.701300
96	H	-1.124943	-4.763268	2.587218
97	C	-4.255108	-2.677965	0.640405
98	H	-3.535100	-3.303544	1.168207
99	H	-4.277997	-1.699598	1.123666
100	H	-5.243028	-3.129515	0.743720
101	C	-4.946905	-1.731122	-1.557115
102	H	-5.921503	-2.217606	-1.490385
103	H	-5.031354	-0.736783	-1.115597
104	H	-4.686984	-1.616551	-2.610414
105	C	-3.844805	-3.950902	-1.440621
106	H	-4.811089	-4.430974	-1.277270
107	H	-3.653978	-3.918082	-2.511934
108	H	-3.072207	-4.563781	-0.977236
109	C	1.536366	2.430058	2.878289
110	H	1.611131	1.456005	3.365250
111	H	1.982680	2.350019	1.886878
112	H	2.113458	3.146680	3.465139
113	C	-0.543291	2.880262	4.162035
114	H	-1.571550	3.239650	4.111716
115	H	-0.550363	1.865350	4.564071
116	H	0.013553	3.519128	4.850002
117	C	0.078576	4.309548	2.206761
118	H	0.675699	4.970606	2.837169
119	H	0.501458	4.316984	1.201298
120	H	-0.934950	4.703054	2.158305

Table S29: Cartesian coordinates for the geometry of tetranuclear **3** in the rhombic conformation optimized using the SMD continuum solvation model.

Center		Coordinates (Å)		
Number	Element	X	Y	Z
1	Au	-0.013771	0.051748	1.710581
2	Au	0.033608	-0.009710	-1.728205
3	Au	-2.454359	-0.025200	-0.046596
4	Au	2.469886	-0.006164	0.019615
5	N	-1.853073	2.426879	1.617944
6	N	-2.683846	1.777276	0.921013
7	N	-0.693896	1.986356	1.878030
8	C	-4.014979	2.433039	0.804561
9	C	0.056167	2.905337	2.779516
10	N	1.803322	2.423744	-1.639503
11	N	0.681130	1.928088	-1.948562
12	N	2.652422	1.812314	-0.925510
13	C	-0.120455	2.897257	-2.752752
14	C	3.943257	2.558859	-0.866786
15	N	1.748208	-2.398159	1.710601
16	N	2.602164	-1.820287	0.979453
17	N	0.628914	-1.878779	1.998887
18	N	-2.590484	-1.840611	-1.008694
19	N	-1.720834	-2.456867	-1.688065
20	N	-0.593187	-1.950863	-1.967914
21	C	0.244331	-2.913147	-2.742010
22	C	3.903491	-2.543076	0.925135
23	C	-0.173764	-2.806589	2.849928
24	C	-3.896811	-2.548569	-0.917912
25	C	4.468721	-2.442897	-0.482887
26	H	3.809901	-2.929519	-1.203355
27	H	4.603124	-1.403858	-0.789778
28	H	5.442456	-2.933976	-0.524900
29	C	3.764098	-4.014924	1.283280
30	H	4.731676	-4.500588	1.143165
31	H	3.457948	-4.159733	2.317460
32	H	3.037699	-4.512391	0.639386
33	C	4.844365	-1.865874	1.913648
34	H	5.831465	-2.332007	1.888193
35	H	4.958561	-0.806514	1.677565
36	H	4.451027	-1.946305	2.928857
37	C	4.685167	2.324469	-2.176195
38	H	5.659250	2.817927	-2.161853
39	H	4.843671	1.257033	-2.343052

40	H	4.111717	2.718632	-3.016649
41	C	4.776823	2.029281	0.286753
42	H	4.248711	2.121564	1.237326
43	H	5.040901	0.981252	0.137763
44	H	5.704938	2.599465	0.355696
45	C	3.712238	4.049944	-0.658837
46	H	4.674156	4.551969	-0.539754
47	H	3.196730	4.499697	-1.505032
48	H	3.120937	4.232259	0.240133
49	C	0.677950	3.360883	-3.963859
50	H	0.058383	4.001562	-4.594510
51	H	1.560337	3.925543	-3.665873
52	H	1.000938	2.504944	-4.560146
53	C	-0.487281	4.080605	-1.869245
54	H	-1.033052	3.739071	-0.988154
55	H	0.409678	4.603961	-1.536430
56	H	-1.114465	4.787961	-2.415866
57	C	-1.387381	2.215590	-3.235510
58	H	-1.161767	1.386639	-3.909177
59	H	-1.973974	1.829283	-2.400677
60	H	-2.001990	2.935507	-3.779373
61	C	-5.037791	1.521323	1.470391
62	H	-6.035694	1.959526	1.408065
63	H	-5.065168	0.544686	0.983712
64	H	-4.791068	1.370095	2.522931
65	C	-4.356037	2.599536	-0.669355
66	H	-4.327305	1.642358	-1.193714
67	H	-5.360882	3.012487	-0.776273
68	H	-3.654276	3.277858	-1.156530
69	C	-4.054075	3.796660	1.474299
70	H	-5.049336	4.222133	1.331998
71	H	-3.861584	3.729803	2.544599
72	H	-3.324831	4.480040	1.039773
73	C	-0.530179	-3.444932	-3.940561
74	H	0.119531	-4.074605	-4.551679
75	H	-1.386968	-4.039804	-3.628329
76	H	-0.888768	-2.621298	-4.561539
77	C	1.486566	-2.200005	-3.244801
78	H	1.230867	-1.396376	-3.938118
79	H	2.062529	-1.772075	-2.422473
80	H	2.123800	-2.911821	-3.773141
81	C	0.653273	-4.054399	-1.822644
82	H	1.173918	-3.663924	-0.946907

83	H	-0.222957	-4.607480	-1.482819
84	H	1.315856	-4.749279	-2.343153
85	C	-1.476688	-2.131115	3.235969
86	H	-1.302319	-1.251642	3.858537
87	H	-2.039869	-1.820955	2.354725
88	H	-2.092246	-2.830162	3.805427
89	C	0.599610	-3.143529	4.117669
90	H	-0.010670	-3.765872	4.775278
91	H	1.516585	-3.684985	3.887806
92	H	0.863255	-2.231977	4.658258
93	C	-0.479874	-4.069677	2.057971
94	H	-1.000450	-3.817859	1.132447
95	H	0.437968	-4.599359	1.802498
96	H	-1.112413	-4.741581	2.641852
97	C	-4.273558	-2.710810	0.547316
98	H	-3.562010	-3.356696	1.063299
99	H	-4.296377	-1.747396	1.060134
100	H	-5.265057	-3.159908	0.630262
101	C	-4.934788	-1.685359	-1.624250
102	H	-5.916123	-2.162080	-1.585962
103	H	-5.014434	-0.705232	-1.150404
104	H	-4.662876	-1.536239	-2.670842
105	C	-3.865637	-3.919529	-1.572848
106	H	-4.843815	-4.386144	-1.441716
107	H	-3.659818	-3.854227	-2.640563
108	H	-3.114921	-4.567286	-1.120568
109	C	1.538957	2.618667	2.624778
110	H	1.791257	1.609155	2.956544
111	H	1.837604	2.717111	1.581177
112	H	2.116646	3.324687	3.224391
113	C	-0.377283	2.637459	4.214255
114	H	-1.442375	2.841676	4.338214
115	H	-0.193705	1.594902	4.482337
116	H	0.177570	3.271550	4.909026
117	C	-0.196639	4.362584	2.420289
118	H	0.440971	5.001012	3.034671
119	H	0.042779	4.549181	1.372042
120	H	-1.232201	4.649338	2.591986

Table S30: Cartesian coordinates for the optimized geometry of tetranuclear **3** in the buckled square conformation.

Center		Coordinates (Å)		
Number	Element	X	Y	Z

1	Au	-0.886877	-1.877651	-0.254754
2	Au	-1.890155	0.906705	0.276249
3	Au	1.911836	-0.881841	0.371930
4	Au	0.890795	1.840738	-0.378961
5	N	-2.520809	-0.475699	-2.327450
6	N	-0.394467	2.584246	2.243268
7	N	0.368504	-2.403788	2.435327
8	N	-0.471901	-2.748621	1.558024
9	N	-2.824486	0.415275	-1.485087
10	N	0.465138	2.851697	1.357102
11	N	-1.261531	1.674979	2.076408
12	N	1.598732	1.127424	-2.174229
13	N	2.538448	0.294225	-2.320514
14	N	2.901165	-0.478642	-1.383579
15	C	2.309497	3.172761	-3.307892
16	H	2.155150	3.828783	-4.166510
17	H	2.119244	3.746459	-2.399039
18	H	3.352442	2.852228	-3.299640
19	C	-3.358255	2.497075	3.031273
20	H	-3.876197	2.274696	2.096626
21	H	-4.073631	2.404707	3.850538
22	H	-3.008362	3.529884	2.992403
23	N	-1.608373	-1.325379	-2.099971
24	C	-2.183929	1.548136	3.234666
25	N	1.236151	-1.507933	2.211201
26	C	2.622571	0.156321	3.309504
27	H	3.260199	0.372435	4.168221
28	H	3.205423	0.326273	2.402044
29	H	1.784184	0.853962	3.308802
30	C	-1.444916	-2.294603	-3.214777
31	C	-2.674996	0.110756	3.278873
32	H	-3.226992	-0.144829	2.372095
33	H	-1.835059	-0.579192	3.368909
34	H	-3.339967	-0.030083	4.132430
35	C	1.384691	1.964613	-3.384101
36	C	0.943239	5.133412	0.688791
37	H	1.568767	6.013277	0.848833
38	H	1.082296	4.795654	-0.339561
39	H	-0.101397	5.422053	0.814309
40	C	2.124522	-1.277361	3.381196
41	C	1.314825	4.029464	1.671951
42	C	-0.005005	-2.777102	-3.196656
43	H	0.228658	-3.291647	-2.262142
44	H	0.168045	-3.472272	-4.019600

45	H	0.680534	-1.935916	-3.298056
46	C	2.778504	3.650685	1.489715
47	H	3.411874	4.517319	1.685712
48	H	3.060549	2.853700	2.177671
49	H	2.976712	3.309531	0.471869
50	C	-0.065695	2.416063	-3.383913
51	H	-0.267724	3.019274	-4.270441
52	H	-0.736662	1.556451	-3.382118
53	H	-0.288736	3.021023	-2.502787
54	C	-3.995790	1.242792	-1.874901
55	C	1.662624	1.184975	-4.661112
56	H	1.383750	1.795887	-5.521077
57	H	2.714258	0.921504	-4.752369
58	H	1.077700	0.265267	-4.689508
59	C	-1.733722	-1.651902	-4.563899
60	H	-2.779610	-1.369074	-4.661172
61	H	-1.125988	-0.757837	-4.706540
62	H	-1.491300	-2.361267	-5.356793
63	C	1.107538	4.527263	3.093352
64	H	1.780923	5.367978	3.267840
65	H	0.085541	4.864241	3.259275
66	H	1.329291	3.748920	3.823307
67	C	-3.631581	2.715160	-1.739002
68	H	-3.324457	2.955712	-0.719487
69	H	-2.814395	2.975001	-2.411957
70	H	-4.494805	3.334436	-1.987921
71	C	-4.441832	0.975493	-3.303461
72	H	-3.639332	1.173246	-4.013895
73	H	-4.767164	-0.054594	-3.439952
74	H	-5.279490	1.635639	-3.534246
75	C	-1.128702	-4.287326	3.412183
76	H	-1.798615	-5.117282	3.642669
77	H	-0.106416	-4.603269	3.614036
78	H	-1.363629	-3.457634	4.078644
79	C	-0.940333	-5.076154	1.063730
80	H	-1.067117	-4.818819	0.010866
81	H	0.102797	-5.353207	1.223342
82	H	-1.567506	-5.941828	1.283587
83	C	3.298503	-2.244784	3.304879
84	H	3.848966	-2.110384	2.372782
85	H	3.985576	-2.080737	4.137141
86	H	2.942809	-3.275435	3.348873
87	C	1.382443	-1.476920	4.694993
88	H	1.088039	-2.514223	4.838685

89	H	2.032410	-1.183618	5.520968
90	H	0.483478	-0.861053	4.730514
91	C	4.081133	-1.294835	-1.791011
92	C	5.188132	-0.378378	-2.298282
93	H	5.452042	0.358754	-1.537936
94	H	6.078253	-0.964554	-2.532676
95	H	4.876923	0.150617	-3.197576
96	C	4.596916	-2.053405	-0.581429
97	H	4.876650	-1.367218	0.219649
98	H	3.850354	-2.750955	-0.197944
99	H	5.481714	-2.626135	-0.863648
100	C	3.677645	-2.283011	-2.875390
101	H	3.299458	-1.755624	-3.751328
102	H	4.537431	-2.883421	-3.178300
103	H	2.900126	-2.955451	-2.511261
104	C	-1.323334	-3.900364	1.954891
105	C	-2.786221	-3.540701	1.731457
106	H	-3.074761	-2.691838	2.351402
107	H	-2.977218	-3.282356	0.688256
108	H	-3.419801	-4.390183	1.991500
109	C	-1.484813	1.873833	4.546652
110	H	-1.197671	2.921628	4.601885
111	H	-2.160099	1.655361	5.375459
112	H	-0.585635	1.269347	4.668194
113	C	-5.132135	0.902932	-0.917560
114	H	-4.832901	1.085486	0.115877
115	H	-6.009412	1.514925	-1.134141
116	H	-5.409782	-0.148017	-1.011134
117	C	-2.395464	-3.463738	-2.990925
118	H	-2.275835	-4.212096	-3.776517
119	H	-2.199256	-3.941363	-2.029368
120	H	-3.430319	-3.117723	-2.997544

Table S31: Cartesian coordinates for the geometry of tetranuclear **3** in the buckled square conformation optimized using the SMD continuum solvation model.

Center		Coordinates (Å)		
Number	Element	X	Y	Z
1	Au	-0.891124	-1.879602	-0.267621
2	Au	-1.891989	0.912852	0.279301
3	Au	1.903170	-0.887803	0.369258
4	Au	0.898780	1.847711	-0.365849
5	N	-2.530266	-0.460224	-2.329300
6	N	-0.394213	2.592548	2.252832

7	N	0.362624	-2.446194	2.415312
8	N	-0.474977	-2.782745	1.531997
9	N	-2.842946	0.416830	-1.475835
10	N	0.464514	2.865155	1.367427
11	N	-1.258980	1.680881	2.082109
12	N	1.615592	1.140780	-2.164055
13	N	2.555825	0.308117	-2.311825
14	N	2.911254	-0.470880	-1.376215
15	C	2.301618	3.201892	-3.287544
16	H	2.149241	3.853429	-4.150484
17	H	2.091495	3.777849	-2.384471
18	H	3.350373	2.899897	-3.264714
19	C	-3.346424	2.505944	3.058113
20	H	-3.878334	2.296266	2.128152
21	H	-4.054760	2.414876	3.884259
22	H	-2.989931	3.537279	3.022898
23	N	-1.606827	-1.301870	-2.110864
24	C	-2.178620	1.546470	3.242678
25	N	1.225772	-1.542040	2.203187
26	C	2.615160	0.107533	3.316468
27	H	3.249338	0.319945	4.179090
28	H	3.201534	0.283729	2.412375
29	H	1.776611	0.805264	3.316744
30	C	-1.428320	-2.251767	-3.240933
31	C	-2.675989	0.111598	3.269365
32	H	-3.231858	-0.131492	2.361419
33	H	-1.837119	-0.581527	3.347004
34	H	-3.338166	-0.040746	4.123572
35	C	1.395942	1.980955	-3.371632
36	C	1.022623	5.113662	0.652689
37	H	1.641831	5.995365	0.828652
38	H	1.227693	4.748823	-0.355252
39	H	-0.026342	5.412187	0.699556
40	C	2.114097	-1.324975	3.376741
41	C	1.321122	4.037299	1.688605
42	C	0.009997	-2.736536	-3.205056
43	H	0.224003	-3.275881	-2.279720
44	H	0.202801	-3.409566	-4.042382
45	H	0.696891	-1.892278	-3.268574
46	C	2.783914	3.625539	1.596761
47	H	3.426056	4.491081	1.770443
48	H	3.021489	2.866857	2.343139
49	H	3.021846	3.220192	0.611110

50	C	-0.060936	2.409524	-3.371622
51	H	-0.273447	3.016861	-4.253268
52	H	-0.716576	1.538052	-3.378506
53	H	-0.296793	3.002272	-2.485405
54	C	-4.019995	1.243115	-1.853124
55	C	1.688114	1.214290	-4.652773
56	H	1.387743	1.821360	-5.508807
57	H	2.746357	0.981521	-4.756052
58	H	1.128407	0.278724	-4.685491
59	C	-1.690195	-1.583604	-4.582918
60	H	-2.736400	-1.308476	-4.702457
61	H	-1.086270	-0.682227	-4.695547
62	H	-1.424221	-2.274334	-5.385263
63	C	1.050155	4.591666	3.077422
64	H	1.720937	5.435536	3.249702
65	H	0.024403	4.944427	3.180786
66	H	1.232176	3.846055	3.851284
67	C	-3.629797	2.713977	-1.809452
68	H	-3.243244	2.993444	-0.827181
69	H	-2.862454	2.933903	-2.552539
70	H	-4.500710	3.337374	-2.020572
71	C	-4.542414	0.911890	-3.241126
72	H	-3.785651	1.080940	-4.007046
73	H	-4.873567	-0.123684	-3.313793
74	H	-5.395380	1.559417	-3.453138
75	C	-1.071737	-4.412502	3.331303
76	H	-1.736373	-5.252566	3.541885
77	H	-0.043876	-4.748434	3.465577
78	H	-1.270434	-3.627603	4.060924
79	C	-1.018419	-5.068854	0.940006
80	H	-1.206594	-4.757882	-0.089148
81	H	0.029311	-5.364952	1.019299
82	H	-1.641599	-5.939480	1.153185
83	C	3.284159	-2.295581	3.294446
84	H	3.843690	-2.151054	2.368914
85	H	3.967037	-2.147723	4.133788
86	H	2.925967	-3.326628	3.320775
87	C	1.371070	-1.532232	4.688524
88	H	1.086181	-2.572499	4.833932
89	H	2.016901	-1.235290	5.517026
90	H	0.466571	-0.923728	4.726225
91	C	4.095925	-1.285018	-1.776811
92	C	5.210373	-0.365335	-2.260002

93	H	5.454974	0.374866	-1.495383
94	H	6.109139	-0.947335	-2.472756
95	H	4.922283	0.162344	-3.168240
96	C	4.595328	-2.055082	-0.568149
97	H	4.874796	-1.377566	0.240596
98	H	3.840566	-2.750382	-0.196190
99	H	5.478597	-2.633174	-0.845897
100	C	3.708034	-2.265116	-2.873756
101	H	3.343230	-1.736061	-3.754646
102	H	4.572713	-2.863698	-3.167992
103	H	2.926713	-2.942628	-2.526305
104	C	-1.331188	-3.937716	1.911262
105	C	-2.794662	-3.537862	1.783291
106	H	-3.040891	-2.737095	2.481466
107	H	-3.025414	-3.192129	0.773563
108	H	-3.435476	-4.393976	2.002702
109	C	-1.472797	1.849235	4.556256
110	H	-1.194832	2.898512	4.635920
111	H	-2.140119	1.607859	5.385756
112	H	-0.567840	1.249314	4.660590
113	C	-5.112421	0.967333	-0.827790
114	H	-4.767739	1.207749	0.179600
115	H	-5.996841	1.571251	-1.039541
116	H	-5.399487	-0.085732	-0.846012
117	C	-2.386029	-3.421278	-3.058715
118	H	-2.261529	-4.150029	-3.862464
119	H	-2.204996	-3.927618	-2.108728
120	H	-3.420431	-3.072126	-3.068498

References

(1) Collings, A. F.; Mills, R. Temperature-Dependence of Self-Diffusion for Benzene and Carbon Tetrachloride. *Trans. Faraday Soc.* **1970**, *66*, 2761–2766.

(2) Goux, W. J.; Verkruyse, L. A.; Salter, S. J. The Impact of Rayleigh-Benard Convection on NMR Pulsed-Field-Gradient Diffusion Measurements. *J. Magn. Reson.* **1990**, *88*, 609–614.

(3) Brown, I. D.; Dunitz, J. D. The Crystal Structure of Diazoaminobenzene Copper(I). *Acta Crystallogr.* **1961**, *14*, 480–485.

(4) Hartmann, E.; Strähle, J. 1,3-Bis(4-Fluorphenyl)Triazenido-Komplexe von Kupfer Und Silber. Synthese Und Kristallstruktur von [Cu(F–C6H4N3C6H4–F)]4, [Ag(F–C6H4N3C6H4–F)]4 Und [Cu(F–C6H4N3C6H4–F)(OCH3)]4. *Z. Anorg. Allg. Chem.* **1990**, *583*, 31–40.

(5) Beck, J.; Strähle, J. Synthesis and Structure of 1,3-Diphenyltriazenidogold(I), a Tetrameric Molecule with Short Gold-Gold Distances. *Angew. Chem. Int. Ed. Engl.* **1986**, *25*, 95–96.

(6) Pyykkö, P. Relativistic Effects in Structural Chemistry. *Chem. Rev.* **1988**, *88*, 563–594.

(7) Bent, H. A. An Appraisal of Valence-Bond Structures and Hybridization in Compounds of the First-Row Elements. *Chem. Rev.* **1961**, *61*, 275–311.
